# Oral and Poster Abstracts of the 13th ISNS European Regional Meeting

**DOI:** 10.3390/ijns11010021

**Published:** 2025-03-10

**Authors:** Kate Hall, Peter C. J. I. Schielen, Dimitris Platis

**Affiliations:** 1 International Society for Neonatal Screening, Reigerskamp 273, 3607 HP Maarssen, The Netherlands; kate@pdsoft.co.uk; 2 Department of Newborn Screening, Institute of Child Health, 11527 Athens, Greece; dplatis@ich.gr

**Keywords:** newborn screening, ISNS, Europe

## Abstract

This Abstract Book contains abstracts of oral and poster presentations of the 13th ISNS European Regional Meeting in Luxembourg, held from 23 to 26 March 2025.

## 1. Introduction

From virtual to live! The 12th ISNS European Meeting was held virtually in ‘Luxembourg’ in 2021 to counter the impact of COVID-19. The 13th ISNS European Meeting, to our great pleasure, will be held in Luxembourg proper at the European Convention Centre. In this online Abstract Book, abstracts are ordered as follows:

I Invited presentations
O Oral presentations selected from poster abstracts
P Poster presentations
  P01 Biotinidase
  P02–10 Congenital Adrenal Hyperplasia
  P11–15 Cystic Fibrosis
  P16–18 Congenital Hypothyroidism
  P19–20 G6PD Deficiency
  P21–37 General
  P38 Hypermethioninaemia
  P39–41 Information Technology in NBS
  P42–43 Isovaleric Aciduria
  P44–49 Lysosomal Storage Disorders
  P50–63 Methodology
  P64 Methyl Malonic Aciduria
  P65–66 Maple Syrup Urine Disease
  P67 Review
  P68–76 Pilot Studies
  P77–79 Phenylketonuria
  P80–84 Severe Combined Immunodeficiency and Spinal Muscular Atrophy
  P85 Tyrosinaemia
  P86 Miscellaneous
  P87 Adrenoleukodystrophy

For brevity, the affiliations of the authors of abstracts for poster presentations are listed only once.

## 2. Invited Presentations

I01. **Keynote Lecture: Reactomics and Its Future in Newborn Screening**
Raquel Yahyaoui
Newborn Screening Laboratory. Málaga Regional University Hospital, Spain

Newborn screening is a critical public health tool that enables early detection of metabolic and genetic disorders, thereby facilitating timely interventions and significantly improving patient outcomes. Traditional screening methods, while effective, are constrained by limitations in sensitivity, specificity, and the spectrum of detectable conditions. In contrast, reactomics offers a transformative, high-throughput approach that simultaneously analyzes multiple enzymatic reactions in dried blood spot samples, generating a comprehensive metabolic profile. This innovative technique enhances diagnostic accuracy by identifying subtle biochemical alterations that traditional assays might overlook, thus reducing false-positive rates and improving overall screening precision. Unlike conventional targeted biochemical assays, reactomics captures a dynamic snapshot of metabolic activity, paving the way for the discovery of novel disease markers and a more precise differentiation between pathogenic and benign variants. In our study, we will utilize array-based human enzyme panels developed by the Spanish company Reactomix, which are capable of measuring the activity of potentially hundreds of enzymes in a single test. This method, characterized by its low cost, minimal sample volume, and automation-friendly design for dried blood spot samples, will be evaluated as part of the MetabPlus study at the Málaga Regional University Hospital. This project, CPP2022-010108, is funded by the Spanish Ministry of Science and Innovation and by the European Union under the “NextGenerationEU”/PRTR framework and is scheduled for 2025 and 2026 (more information at www.metabplus.com). Furthermore, reactomics integrates seamlessly with other omics technologies, such as genomics and proteomics, to provide a holistic approach to disease characterization. This multi-layered analysis is expected to refine diagnostic algorithms, enhance patient stratification, and open new avenues for personalized treatment strategies. With ongoing advancements in automation and computational analysis, reactomics stands out as a scalable and cost-effective solution for large-scale newborn screening programs, with the potential to revolutionize early disease detection and optimize neonatal healthcare worldwide.

- I02. **The World of Neonatal Screening in Numbers and Europe Compared**
- Peter C. J. I. Schielen
- International Society for Neonatal Screening, Stichtse Vecht, The Netherlands

Data on the current state of neonatal screening around the world remains incomplete with the majority of data collected concentrated in Europe, North and Latin America. The International Society for Neonatal Screening (ISNS) made it its core responsibility to build a comprehensive and complete picture of that current state and significant milestones have been reached, with the help of many colleagues. ISNS started with the development of a vehicle to present global data on its website in May 2023. January 2025 the so-called digital global maps for neonatal screening were released on the ISNS-website. Population with data of the so-called digital maps was done using key publications on the regional and global state of neonatal screening, standardized surveys issued by the ISNS, sent to regional and national contacts, data of manufacturers of NBS analytical tests (who often have complementary data on screening activity), and finally other open source data and grey literature. In this presentation, the now fully functional on-line maps of neonatal screening will be demonstrated, with special reference to data of Europe. The ambition to build a sustainable network of committed contacts who can be approached for updates that can then be incorporated, following checks, into the accumulating data set by an agreed panel of moderators, and especially the challenges to sustain such a network, will also be discussed. We hope that the results of this activity will act as a valuable resource and a stimulus to inspire and inform national health policy makers and others who may wish to help establish and expand well organized and sustainable newborn screening linked to treatment where this is appropriate. This online available database reflects the mission and values that lie at the heart of the ISNS to offer the life changing benefits of newborn screening in the context of carefully planned and evidence based policy. The ISNS cannot do that without the continued support of colleagues and partnering organizations and companies.

I03. **The Spectrum of Approaches to Neonatal Screening Around the World, Europe, and Unmet Needs**
Urh Groselj ^1,2,3^

^1^  Faculty of Medicine, University of Ljubljana, Ljubljana, Slovenia
^2^  Department of Endocrinology, Diabetes, and Metabolic Diseases, University Children’s Hospital, University Medical Centre Ljubljana, Ljubljana, Slovenia
^3^  Center for Rare Diseases, University Children’s Hospital, University Medical Centre Ljubljana, Ljubljana, Slovenia

Newborn screening (NBS) is a cornerstone of public health, enabling the early detection and treatment of congenital disorders to improve long-term outcomes. However, the implementation of NBS varies considerably worldwide due to differences in healthcare infrastructure, economic resources, and policy frameworks. In Europe, NBS programs have evolved since their inception in the mid-1960s, initially screening for conditions like phenylketonuria. The introduction of tandem mass spectrometry in from the early 2000s expanded screening capabilities, allowing the detection of metabolic, endocrine, hematologic, and genetic disorders. Despite these advancements, substantial heterogeneity exists across European countries regarding the scope and methodology of NBS programs. While some nations screen for over 50 conditions, others test for fewer than ten, while several for 0–2 conditions only, reflecting disparities in national policies, funding, and public health priorities. This variability presents critical challenges, particularly in ensuring equitable access to early diagnosis and treatment. The absence of a standardized European screening panel means that a newborn’s diagnostic opportunities depend largely on their country of birth. Efforts such as the Screen4Rare initiative aim to address these discrepancies by fostering collaboration, standardizing screening criteria, and advocating for policy harmonization. Beyond Europe, global disparities in NBS are even more pronounced. Low- and middle-income countries often lack the infrastructure and funding to implement comprehensive screening, leaving many treatable conditions undiagnosed. Innovative solutions and international partnerships are essential to bridging these gaps. To advance NBS globally, future efforts must focus on expanding access, integrating genomic technologies, and promoting international policy alignment. A more standardized, evidence-based approach to neonatal screening will be crucial in reducing disparities and ensuring that all newborns receive the benefits of early disease detection and intervention.

I04. **A Global View of Genomic Sequencing in Newborn Screening: Where Are We Now and Where Are We Going?**
David Bick
Genomics England, Ltd.

More than 800 treatable genetic disorders are known (https://www.rx-genes.com/). Adding conditions singly to current newborn screening cannot keep pace with the growing list of treatable conditions. Therefore, research programs worldwide are evaluating the clinical utility of genome sequencing to screen for treatable conditions. A survey of genomic screening programs underway or planned finds 5 programs in North America, 10 in Europe, 1 in Russia, 3 in the Middle East, 3 in Australia, 9 in China, 1 in Taiwan and 1 in Korea. Private companies have started to offer newborn genome screening on a self-pay basis. Because so little is known about the use of genome screening in the newborn setting a wide range of strategies are being pursued to find the best time to approach parents, choose genes and associated conditions, select variants for reporting, identify an appropriate genomic test and sample type, and optimize reporting and treatment. The strong international interest in the potential of newborn genome screening has given rise to the International Consortium on Newborn Sequencing (https://www.iconseq.org/) organized to exchange learnings from the diverse efforts of the numerous programs. In the next year programs will communicate the results of thousands of screened newborns. Developing ways of sharing the outcome of screen positive cases across programs and assuring access to treatment represent long term challenges. As the price of short read and particularly, long read genome sequencing fall, the availability and accuracy of newborn genome screening will improve.

I05. **Population-Based, First-Tier Genomic Newborn Screening in a Single Maternity Ward in Belgium: Interim Results of BabyDetect Project**
Boemer François
CHU Liege, Avenue de l’Hôpital, 1, 4000 Liege, Belgium

The rapid development of therapies for severe rare genetic conditions underlines the need to incorporate first-tier genetic testing into newborn screening (NBS) programs. A workflow was developed to screen newborns for 165 severe pediatric treatable disorders by deep sequencing of regions of interest in 405 genes. The prospective BabyDetect pilot project was launched in September 2022 in a maternity ward of a large public hospital in the Liege area of Belgium. To date, about 6100 families have been informed of the project, and 5545 consented participate in the trial. We identified 103 cases of disease using deep sequencing; 32 of these were not detected by conventional NBS. G6PD deficiency was the most frequent disorder detected, with 69 positive individuals. Of the remaining 34 cases, 20 were recessive disorders. We identified two false positive case: one case of SCAD, which was not confirmed by orthogonal methods and one in a newborn where two variants were identified in the AGXT gene, which were subsequently shown to be both on the maternal allele. Ten heterozygous variants were identified in genes described as having a dominant mode of inheritance. The BabyDetect pilot project results demonstrate the importance of integrating biochemical and genomic methods in NBS programs. However, clinical, economic, societal, legal, and ethical considerations must be addressed. A recall rate of one in 54 newborns (103 cases/5545) is challenging to manage in a population-based NBS context. To minimize the psychosocial impact of such a recall rate, we recommend that subjects with mild G6PD deficiencies should not be reported. Challenges must be addressed in variant interpretation within a presymptomatic population and in result reporting and diagnostic confirmation of screening results.

I06. **Decoding Uncertainty: Variants of Unknown Significance and the Limitations of Genomics without Biochemistry**
Asbjörg Stray-Pederson ^1^ and Jernej Kovac ^2,3^

^1^  Norwegian National Unit for Newborn Screening, Division of Paediatric and Adolescent Medicine, Oslo University Hospital, 0424 Oslo, Norway
^2^  Faculty of Medicine, University of Ljubljana, Vrazov trg 2, 1000 Ljubljana, Slovenia
^3^  Clinical Institute for Special Laboratory Diagnostics, University Children’s Hospital, Ljubljana University Medical Center, Vrazov trg 1, 1000 Ljubljana, Slovenia

As genomic sequencing technology becomes increasingly accessible, cost-effective, and rapid, the possibility of implementing first-tier genomics in newborn screening is gaining traction. This paradigm shift promises earlier detection of treatable conditions, but it also introduces challenges that cannot be ignored. Unlike conventional biochemical screening, which provides immediate phenotypic context, a genomics-only approach lacks the ability to directly measure metabolic derangements, leading to uncertainties in variant interpretation. One of the most pressing concerns is the classification of variants of uncertain significance (VUS) and the detection of pathogenic variants in regions with low sequencing coverage. Additionally, structural and deep intronic variants, which may not be captured by standard sequencing pipelines, could result in false-negative findings if no biochemical markers guide further investigation. Familial Hypercholesterolemia (FH) serves as a compelling model for exploring these limitations. In Slovenia, preschool children undergo biochemical screening for FH, providing a real-world example of how integrating biochemical markers with genomic data enhances diagnostic accuracy. Without LDL cholesterol measurements, many at-risk children could be overlooked or misclassified based on genomic data alone. Beyond the limitations of detection, a genomics-only approach raises practical and ethical dilemmas in reporting results. The question of whether to disclose VUS findings remains contentious, particularly in the context of newborn screening, where false alarms can induce unnecessary parental anxiety and burden healthcare systems. Automatic classification pipelines, which rely heavily on pre-existing databases and computational predictions, can struggle with edge cases, leading to inconsistencies in variant interpretation. Furthermore, observational error statistics will be discussed to quantify the uncertainty inherent in genomics-only screening models. From a laboratory perspective, sequencing-based newborn screening also presents challenges related to turnaround time, wet-lab processing complexities, and result interpretation workflows These factors could delay timely intervention and overwhelm screening laboratories with unresolved cases, posing a significant concern, especially for disorders that require urgent treatment. Through practical case examples, this lecture will demonstrate how integrating biochemical and genomic data in a structured testing algorithm improves diagnostic reliability and clinical decision-making. Ultimately, while genomics holds immense potential for newborn screening, its implementation without biochemical corroboration introduces significant risks that must be carefully navigated.

I07. **Untargeted Metabolomics: Great Potential for the Discovery of New Biomarkers for Inclusion in Newborn Screening**
Leo A.J. Kluijtmans
Translational Metabolic Laboratory, department of Human Genetics, Radboud University Medical Center Nijmegen, The Netherlands

Newborn screening (NBS) is a vital public health program aimed at the early detection of treatable (metabolic) disorders. Current NBS methodologies rely largely on targeted analysis of predefined metabolites using tandem mass spectrometry (MS/MS). Untargeted metabolomics, using high-resolution mass spectrometry combined with advanced data analysis, is an analytical approach for comprehensive profiling of metabolites without prior selection. This technique enables the discovery of novel disease biomarkers that can subsequently be incorporated into targeted screening assays, such as those in NBS programs. This lecture will explore the potential of untargeted metabolomics in advancing NBS by facilitating the identification of new disease biomarkers. Several examples of novel biomarkers identified through untargeted metabolomics will be presented, including those for phenylketonuria (PKU), classical homocystinuria (CBS deficiency) and pyridoxine-dependent epilepsy (PDE; ALDH7A1 deficiency). These cases will illustrate how untargeted metabolomics holds great potential for expanding and improving newborn screening programs by enabling the discovery of novel biomarkers for inherited metabolic disorders. By integrating these newly identified biomarkers into the NBS programs, we can enhance diagnostic accuracy, expand the NBS programs with new treatable diseases, improve early disease detection, and ultimately optimize neonatal health outcomes.

I08. **Practical Lessons in Integrating Genetics into the Daily Workflow in a National Neonatal Screening Unit Setting**
Janne Strand, Emma Lundman, Mari Ytre-Arne, William Tourniaire, Silje Hogner, Ruth Halsne, Alexander Rowe, Cassandra Trier and Asbjørg Stray-Pedersen
Department of Newborn screening, Division of Paediatric and Adolescent Medicine, Oslo University Hospital, Norway

In 2012, the Norwegian newborn screening program expanded from two to 23 disorders, and the legislation governing the screening program was altered to allow for genetic analysis of the screening disorders. In subsequent years, the screening laboratory has continuously developed genetic methods to improve and expand newborn screening (currently 39 disorders), contributing to an increase in positive predictive value from 26% in 2012 to 95% in 2024. In addition to first-tier DNA-based methods for SCID, SMA and sickle-cell anemia, second-tier genetic analysis is triggered by a fixed set of cutoff values for each disorder. DNA is extracted from one to two 3 mm punches from the original blood spot filter cards, followed by downstream analysis using either Sanger sequencing, NGS gene panels or filtered whole genome sequencing—depending on the condition. Turnaround time from extraction to sequencing results is one to three days. The urgency and severity of a disorder determines whether or not the biochemical results alone trigger immediate reporting, or whether to report based on the genetic findings. Genetic results from the most urgent samples still provide valuable support for follow-up. Genetic information can be of great value but can also be hard to interpret. Variants of uncertain significance and mild variants can confuse as much as support decision making and the sheer volume of variants can appear overwhelming. Nonetheless, the combination of biochemical and genetic data has been a very valuable asset to the Norwegian NBS, and our continuously developing knowledge and experience of genetic interpretation helps develop high precision screening that will identify the children in need of follow-up, while leaving the families of healthy children alone.

I09. **Management of Duchenne muscular dystrophy: how newborn screening could be game-changing**
Laurent Servais
University of Oxford, England, UK

Duchenne Muscular Dystrophy is a devastating condition that affects 1/5000 boys. It leads to loss of ambulation before the age of 15 and the current life expectancy is about 30 years. After decades of research some drugs have shown minor benefit in children older than 5 years. These drugs can be mutation dependants such as oligonucleotides that could be used in about 30% of DMD boys or mutation independants such as vamorolone or Givinostat. In addition, the recent FDA approval of gene therapy despite negative phase III results has questioned the optimal time window for gene therapy and trials are ongoing to infuse patients as young as 6 months. In this lecture, I will discuss how these recent developments could modify our vision of NBS of Duchenne Muscular Dystrophy—including on the method used for screening.

I10. **Bloodspot Screening for Duchenne Muscular Dystrophy, the Past and the Future**
Stuart J. Moat
Wales Newborn Screening Laboratory, University Hospital of Wales, Cardiff, UK

Duchenne muscular dystrophy (DMD) is a progressive, lethal X-linked neuromuscular disorder and is one of the ten most severe and common paediatric genetic diseases. DMD affects an estimated 1 in every 5000 male births, and the mean age of diagnosis is 5 years of age. The development of bloodspot creatine kinase (CK) enzyme assays in the 1970’s paved the way for newborn boys to be screened for DMD. Despite the fact that no curative treatment for DMD existed, several screening programmes were piloted/implemented worldwide during the last 40 years, with the aim to reduce diagnostic delay, enable informed reproductive choice and allow early intervention with steroids and physiotherapy. In the last few years there has been a renewed interest in implementing newborn screening for DMD as advances in molecular therapeutics have led to the development of drugs that can decelerate the deterioration in muscle function. This presentation will highlight the successes, difficulties and lessons learned from past programmes to guide future newborn blood spot screening programmes for DMD.

I11. **Two Years of Spinal Muscular Atrophy (SMA) Screening in the Netherlands and the Treatment Options**
W Ludo van der Pol ^1^, Els Voorhoeve ^2^, Inge Cuppen ^1^, Fay-Lynn Asselman ^1^, Miriam de Pagter ^3^, Gert Weijman ^2^, Henk Engel ^4^ and Sandra Imholz ^2^

^1^  Department of Neurology, Brain Center Rudolf Magnus, UMC, Utrecht, The Netherlands
^2^  National Institute for Public Health and the Environment, Bilthoven, The Netherlands
^3^  Genetics, UMC, Utrecht, The Netherlands
^4^  Department of Clinical Chemistry, Isala Hospital, Zwolle, The Netherlands

Spinal muscular atrophy (SMA) is a severe neuromuscular disorder caused by homozygous loss of function of the *survival motor neuron 1 (SMN1)* gene and characterized by progressive weakness. Its natural history is one of infantile death for cases with early onset (i.e., SMA type 1) and severe disability for chronic forms (type 2 and 3). Three genetic treatments have been introduced recently, i.e., nusinersen, onasemnogene abeparvovec (OA) and risdiplam. Early treatment gives the best results and newborn screening (NBS) programs for SMA are therefore implemented in a growing number of countries. Our objective is to describe our first two-and-half years’ experience of NBS for SMA. Data was collected from June 2022–January 2025 by the national SMA Center that provides SMA genetic treatment for all patients in our country (population 18 million). NBS uses multiplex qPCR to detect homozygous deletion of *SMN1*. After referral, we use a diagnostic test to confirm the result on a new sample and to determine *SMN2 copy number*, an important predictor of disease severity. We identified 43 newborns with SMA (0.0096% of live births). Median referral age was 9 days (IQR 7–10), followed by visit to outpatient clinic 1 day (IQR 1–3) later. Another 3 days (IQR 2–3) later diagnostic test results were known. Two infants had 1 *SMN2* copy, 17 had 2 *SMN2* copies, 18 had 3 *SMN2* copies and 6 had 4 *SMN2* copies. Baseline motor function was determined with CHOP-INTEND (maximum score 64). Median score was 40 (IQR 31–51) in infants with 2 *SMN2* copies and 54 (IQR 52–55) in infants with 3 or 4 *SMN2* copies. Median age at start of treatment was initially 25.5 days (22.5–31.5) but decreased to 10.5 days (IQR 9–15.5) from April 2024 due to a new reimbursement arrangement. Seven infants, two with 1 *SMN2* copy and five with 2 *SMN2* copies, weren’t treated because of poor clinical condition. Additionally, 2 infants with 2 *SMN2* copies died despite treatment with OA and risdiplam. In total 9 out of 43 (20.1%) infants died (expected mortality based on natural history 19/43 (44.2%). A successfully implemented newborn screening program for SMA resulted in the identification of 43 infants. We succeeded in reducing treatment delays and NBS reduced early mortality. Continued follow-up is needed to learn about the long-term effects of early treatment.

I12. **Registries Are Key to Precision Medicine and Improvement of Screening Programmes**
Stefan Kölker, Florian Gleich, Kathrin Mengler, Svenja Scharre, Julia Stengel, Anna Reischl-Hajiabadi, Alina Klein, Lucy Henze, Elena Schnabel-Besson, Kathrin Jeltsch, Nikolas Boy, Georg F. Hoffmann, Friederike Hörster, Jürgen G. Okun, Thomas Opladen, Sven F. Garbade, and Ulrike Mütze on behalf of the Unified Registry for Inherited Metabolic Disorders (U-IMD) Consortium, the European Registry and Consortium for Intoxication-type Metabolic Diseases (E-IMD), the European Reference Network for Hereditary Metabolic Disorders (MetabERN), and the longitudinal observational NBS study in Southwest Germany (NBS 2025)
Heidelberg University, Medical Faculty, Centre for Pediatric and Adolescent Medicine, Department of Pediatrics I, Division of Pediatric Neurology and Metabolic Medicine, Heidelberg, Germany

Having the potential to pool data, to achieve sufficient sample size, to overcome the knowledge gap on rare diseases and to foster epidemiological and clinical research, patient registries are recognised as key instruments to precision medicine for individuals with rare diseases. In line with this, evidence is increasing that registry-based longitudinal observational studies are powerful tools to guide screening strategy for rare diseases. Newborn screening (NBS) programmes aim to promote health through shortening the time to diagnosis and specific treatment. Although first NBS programmes have been introduced in the 1960s and since then have been gradually extended, formal evidence of the long-term clinical benefits in large cohorts and cost-effectiveness of current NBS programmes is still scarce. Registry-based observational studies could fill this gap. They confirmed important benefits of NBS programmes but at the same time they also unraveled a significant number of limitations. These limitations include suboptimal process quality, an incompletely understood natural history and unexpected phenotypic diversity, unreliable early and precise prediction of individual disease severity, uncertainty about case definition, risk stratification, and indication to treat, resulting in a diagnostic and treatment dilemma in individuals with ambiguous screening and confirmatory test results, as well as unreliable therapeutic efficacy and quality for some target diseases included in current NBS programmes. Considering the enormous beneficial potential of data-driven optimisation of NBS programmes and standing at the edge of utilising high-throughput molecular genetical technologies for NBS programmes, it seems overdue to build an international collaborative framework that enables standardised collection and exchange of key performance indicators and outcome data in a protected environment, integrating the perspectives of patients, families, and the society, and, finally, guides evidence-based optimisation of NBS programmes.

I13. **Studies of Long-Term Outcome for CH and CAH in Sweden—Examples of Using Unique, Existing National Registers**
Anna Nordenström ^1^, Rolf Zetterström ^1^ and Anna Gunnerbeck ^2^

^1^  Centre for Inherited Metabolic Diseases, Karolinska University Hospital Solna, Stockholm, Sweden
^2^  Dept Women’s and Children’s Health and Department of Molecular Medicine and Surgery, Karolinska Institute.

Neonatal screening is performed at one National laboratory in Sweden. The screening for congenital hypothyroidism (CH) started in 1980 and for congenital adrenal hyperplasia (CAH) in 1986. Sweden has unique national registries for diagnoses, drug prescription, mortality, cause of mortality, civil status, multi-generation and education and school performance. Patients identified with CH in the screening and in the national diagnosis registry were compared. To our surprise almost as many children were diagnosed with CH later during the first year of life as were identified through the screening. We could show that the effect of lowering the screening cut-off level for CH led to more children being detected by the screening. Simultaneously fewer children were diagnosed with CH during the first year of life and the total incidence was stable over the years. The birth characteristics differed between those diagnosed through screening and not. Individuals diagnosed with CH through neonatal screening performed slightly worse at school than the general population. The difference was larger when comparing with siblings. Children with CH diagnosis and a normal screening result, and thus diagnosed later, presented the lowest results on grade point sum and national tests. A national registry for CAH was created at the laboratory including information about severity of disease comprising more than 600 patients born before and after screening, and in >80% with known CYP21A2 genotype. This has enabled large epidemiological registry studies. We saw that CAH results in increased mortality, with adrenal crises and infectious diseases as main causes of death. We could also show an increased cardiovascular morbidity. School performance was negatively affected in patients with the most severe forms of CAH, especially for the females.

I14. **Dried Bloodspot Storage, Biobanking—European Inventory and Legal Issues**
Mette Nyegaard
Department of Congenital Disorder, Statens Serum Institute, Copenhagen, Denmark

Newborn screening through newborn dried blood blood spots is one of the most successful public health programs prevent disability, disease, and death in children. The newborn dried blood spots also hold immense potential for biomedical research serving as a single repository of specimens from infants across all geographic and demographic segments of a population. In Denmark, more than 40 research projects utilizing blood spots from in total 250,000 neonates been approved by research ethics committees since 2012. The projects have provided unique results and new insights; however it has also put pressure on this scarce resource, and a recent media interest, extending even into national television, has now fueled an active discussion in Denmark on the legal and ethical basis of biobanking of dried blood spots and the use of dried blood spots in research. To gain an overview of the current state of biobanking and/or storage of dried blood spot cards in Europe, the International Society of Neonatal Screening (ISNS) sent out a questionnaire with questions regarding biobank size, storage time, storage conditions, policies for using material for research, and guidelines that determine what secondary research is approved, etc. This presentation will provide an overview of responses from over 20 screening centers, highlighting significant differences in governance across countries. It is the hope that this overview will result in discussions on how to ensure the integrity of PKU biobanks, balancing ethics and the potential that these collections holds for research.

I15. **Experiences Screening Older, Previously Unscreened, Children in the Swedish DBS Newborn Screening Program**
Lene Sörensen ^1,2^ and Rolf H Zetterström ^1,2^

^1^  Centre for Inherited Metabolic Diseases, Karolinska University Hospital Solna, Stockholm, Sweden
^2^  Department of Molecular Medicine and Surgery, Karolinska Institutet, Stockholm, Sweden

Sweden has had a national newborn screening program using dried blood spots (DBS) since screening for PKU was introduced 1965. With the introduction of screening for spinal muscle atrophy (SMA) in 2023, 26 diseases are now included in the program. In Sweden, older, not previously screened, children are offered screening through the national DBS screening program using a slightly modified test panel. Most commonly, these children are immigrants or international adoptions. The recommendation from the National Board of Health and Welfare is that all children up to eight years of age should be offered screening within this program. The PKU laboratory accepts DBS screening samples from all children, i.e., any individual up to 18 years of age. We have shown previously that the incidence of screening diseases among older, previously unscreened, children in Sweden is approximately twice that of the incidence in the newborn population (poster presentation, ISNS Luxembourg virtual meeting, 2021). It was however unclear if this was due to a sampling bias, as we did not have data regarding screening coverage in relation to the national recommendation. Screening coverage of the Swedish newborn population is greater than 99% with no measurable non-participation. In this talk, we present updated incidence data for the years 2010–2023, as well as data on screening coverage of the two age groups 0–7 years and 8–17 years, i.e., children within or older than the national recommendation. We also discuss challenges we have encountered, and experiences gained, offering screening to older children. Between 1 January 2010 and 31 December 2023, roughly 48,000 older children, defined as older than 30 days of age at the time of sampling, were screened through the Swedish DBS screening program. During the same period more than 1.6 million newborns were screened.

I16. **Mutation Analysis in Congenital Hypothyroidism—Clear Links and Cautionary Tales**
Alexander D. Rowe ^1^, Rolf H. Zetterström ^2^, Julie Hellem Aaby ^1^, Silje Hogner ^1^, Janne Maren Strand ^1^, Asbjörg Stray Pedersen ^1^, Mikael Oscarson ^2^ and Anna Wedell ^2^

^1^  Department of Newborn Screening, Division of Paediatric and Adolescent Medicine, Oslo University Hospital, Norway
^2^  Centre for inherited metabolic diseases, Karolinska University Hospital and Department of Molecular Medicine and Surgery, Karolinska Institutet, Stockholm, Sweden

In the Swedish newborn screening for congenital hypothyroidism (CH), the TSH cut off value for referral is 15 mU/L whole blood resulting in approximately fifty children per year being identified with today’s birthrates. In 2015 whole genome sequencing (WGS) was introduced at Karolinska University Hospital as a clinical diagnostic test primarily focusing on inborn errors of metabolism. Already from the start a panel for identifying genetic causes of CH was also set up, a panel at present consisting of 17 genes (https://www.karolinska.se/4a74f9/globalassets/global/2-funktioner/funktion-kul/cmms/ch-v1.pdf). From the start of diagnostic WGS until the 24th of February this year thirty-two children have been analyzed. In twelve cases a genetic cause was found. The most prevalent affected gene was TSHR, but homozygous pathogenic variants in TPO, SLC26A7 and TG have also been identified. For many years the Norwegian approach was to accept a high(er) false positive rate, and set a TSH cutoff at 10 mU/L whole blood to also catch and treat cases of CH with a TSH value between 10 and 15 mU/L. Many of the other disorders screened for in Norway rely on rapid second-tier genomics, including WGS, to confirm or refute a first tier finding, resulting in an overall extremely high positive predictive value. However, in the case of CH-screening, the link between TSH values, CH and genetics is less consistent and genomics cannot be used to improve the precision of CH screening. Retrospectively WGS sequencing all referred CH samples from a 5-year period (~300) on a bioinformatically filtered panel of genes like the ones used at Karolinska University Hospital, has given us a detailed overview of the variants found across a range of TSH levels. As expected, many CH cases could not be resolved using genetics and the most common findings were in the same set of genes identified in Sweden. In conclusion WGS has been used successfully for several CH patients in which a genetic background was considered likely. An overview of genetic findings when sequencing samples from a broader CH population will also be demonstrated and discussed.

I17. **Use of AI and Automated Bloodspot Punch Machines to Evaluate Bloodspot Quality**
Nick Flynn
Biochemical Genetics Unit, Cambridge University Hospitals NHS Foundation Trust, Cambridge, UK

Dried blood spot (DBS) size and quality affect newborn screening (NBS) test results and can contribute to a false negative screen. To ensure reliable testing, newborn screening laboratories routinely reject poor quality DBS specimens. However, visual assessment of DBS quality is subjective and can vary between and within laboratories. Automated image analysis offers an objective approach to measure DBS size and assess quality. Several methods have been described using images obtained from standalone equipment, smartphone cameras or DBS punchers, enabling convenient and reproduceable DBS size and/or quality assessment. This talk will present the development and validation of a computer vision (CV) algorithm to measure DBS diameter and identify incorrectly applied blood in images from a DBS puncher. The algorithm was used to monitor historical trends in DBS quality on >175,000 DBS specimens, and to correlate DBS diameter to analyte concentrations on >130,000 DBS specimens, confirming the results of experiments performed on contrived DBS specimens. Image analysis of DBS specimens could be used as a decision support tool in newborn screening laboratories to aid assessment of DBS quality. CV could also be used to provide an objective comparison of DBS quality between different areas or over time to support efforts to improve DBS quality improvements. The ability of computer vision algorithms to precisely measure DBS size highlights the importance of precisely defining the measurand for DBS diameter. Different definitions can have a small but easily avoidable impact on measurement, impacting the proportion of samples not meeting DBS quality acceptance guidelines. An agreed definition would facilitate harmonisation of assessments between images obtained by sample collectors, for example on smartphones, and those obtained by laboratory equipment.

I18. **IFCC/ISNS Global Task Force**
James R. Bonham
Sheffield Childrens (NHS) FT, Sheffield, UK

It is more than sixty years since Robert Guthrie described a dried blood spot method which could be used to screen for PKU and other disorders shortly after birth. Since that time the number of babies tested and the number of conditions included, have markedly increased and as a result, more than 500,000 babies have benefitted from the life changing intervention offered by newborn screening. In 2025 it is estimated that more than 45 m babies around the world will receive some form of biochemical newborn screening and while a significant achievement, it leaves 95 m babies without this opportunity—almost all of these are in low and middle income countries. A joint initiative between IFCC and ISNS formed in 2021 and, working alongside others, has sought to address this need and to support the introduction of appropriate and well organised newborn screening in LIMC settings and to help with the expansion of existing NBS programmes where this is needed. Even within Europe the need to develop and expand very limited screening could bring significant benefit to many. While international support is important, planning and delivery need to be firmly rooted in the country to ensure sustainability and ownership. It is also clear that to be effective newborn screening needs to be delivered as part of a coordinated public health programme, designed to link sample collection and transport to testing and results to effective treatment. In this talk we will look at some of the key factors, such as committed advocacy, patient and governmental support and legislation that can support the introduction of effective and well-organised newborn screening.

I19. **Our Journey from Individual Efforts to Nationwide Support: Implementing Newborn Screening for SMA and Other Disorders in Serbia**
Miloš Brkušanin, Nemanja Garai and Dušanka Savić-Pavićević
Centre for Human Molecular Genetics, Faculty of Biology, University of Belgrade, 11,000 Belgrade, Serbia

Biochemical screenings in Serbia began in the 1980s with phenylketonuria and congenital hypothyroidism, later expanding to include cystic fibrosis in 2021. These screenings are regionally organized, with the autonomous province of Vojvodina conducting these tests at a single clinical center, and a separate center in Belgrade serving the rest of the country. A transformative shift occurred in 2021 with the launch of Serbia’s first genetic newborn screening initiative for Spinal Muscular Atrophy (SMA), centralized at the Center for Human Molecular Genetics, an institution with expertise in SMA research and diagnostics since 1997. Despite being part of the Faculty of Biology rather than the healthcare system, this institution was designated by the Serbian government as the sole facility with the expertise and capacity to perform nationwide SMA screening. The implementation of SMA NBS was driven by the availability of innovative SMA treatments in Serbia and the proven benefits of early intervention. The process involved assay validation, collaboration with patient organizations and healthcare professionals, a feasibility study, and negotiations with public health and government officials. The feasibility study, which detected three clinically silent SMA cases, led to the official integration of SMA NBS into Serbia’s national healthcare program on 15 September 2023. Since its launch, 89,854 newborns have been screened, identifying 10 SMA-positive infants, all of whom received timely therapeutic interventions. Notably, no false-negative results have been observed. This initiative underscores the vital collaboration between academia, patient advocacy groups, and industry in advancing healthcare innovations. The successful implementation of SMA NBS demonstrates its transformative potential in improving outcomes for affected infants and their families. Looking forward, the success of SMA NBS lays the foundation for expanding genetic NBS in Serbia using advanced and targeted sequencing technologies, marking the dawn of a new era in neonatal care.

I20. **Newborn Screening in Georgia, Problems and Future Perspectives**
Nazi Tabatadze ^1,2^

1. Department of Pediatrics, MediClubGeorgia Medical Center, Tbilisi, Georgia
2. Tbilisi State Medical University, Tbilisi, Georgia

Georgia is a transcontinental country located at the crossroads of Eastern Europe and Western Asia, covering an area of 69,700 square kilometers and with a population of approximately 3.7 million people. The country faces significant challenges in the healthcare sector, particularly in relation to inherited metabolic disorders. Despite the implementation of a state newborn screening (NBS) program in 2003, comprehensive data on the prevalence of inborn errors of metabolism is still lacking. The NBS program, managed by the LEPL “National Health Agency of Georgia”, initially screened all newborns for three genetic conditions: Phenylketonuria (PKU), Cystic Fibrosis (CF), and Congenital Hypothyroidism (CH). Screening was performed by a private laboratory using the fluorometric method. However, the program faced several limitations, including the lack of standardized protocols, local guidelines, and the occurrence of false-negative results, particularly for PKU. In September 2024, the program was transferred to a new private laboratory, and its scope was expanded to include additional disorders such as Congenital Adrenal Hyperplasia (CAH), Galactosemia, and Biotinidase deficiency, with ELISA now used for analysis. Despite these advancements, significant gaps remain, including the absence of population-based data on cut-off values, disease prevalence, and false-positive rates. Notably, the highest false-positive rates have been observed for biotinidase activity and 17-hydroxyprogesterone. These challenges highlight the need for improved technology, trained staff in a lab setting, better public awareness and better coordination among the various components of the state and screening program. Strengthening Georgia’s national NBS program and addressing its gaps is crucial for improving the program’s effectiveness in the early detection and intervention of inherited metabolic disorders.

I21. **Newborn Screening in Romania**
Dorica Dan
president RONARD—Romanian National Alliance for Rare Diseases, coordinator NoRo Centre, vice-president EURORDIS, ePAG chair ERN Ithaca, co-chair WHO TAG Disability & Health, National Council for Rare Diseases, secretary

Romania is implementing a national program for new-born screening since 2009 and screens only for 3 diseases: phenylketonuria (PKU) congenital hypothyroidism (CH) and CF Cystic Fibrosis. The main obstacles include limited financial resources, lack of political will, and insufficient human resources. METABO.MS pilot project, coordinated by the University of Medicine and Pharmacy “Iuliu Hațieganu” in Cluj-Napoca in partnership with Romanian National Alliance for Rare Diseases, Medfuture, and Reykjavik University has shown promising results in expanding neonatal screening in Romania. The project tested approximately 10,000 new-borns from 11 counties, including Bihor, Bistrița-Năsăud, Caraș-Severin, Cluj, Harghita, Maramureș, Mureș, Sălaj, Satu Mare, Sibiu, and Timiș. The project tested approximately 10,000 new-borns from 11 counties (aprox ¼ of the country), established the first laboratory in Romania dedicated to mass spectrometry-based neonatal screening and initiated a new electronic registry for NBS. It also included training activities for laboratory staff to ensure the sustainability and expansion of this advanced testing technique. Another pilot screening project for Spinal Muscular Atrophy (SMA) in Romania has shown promising results. Several multi stakeholders’ meetings have been organized to advocate for upscaling NBS in Romania, including participation from current national coordination centre for NBS, policy makers, experts, patient organizations, METABO ERN (Maurizio Scarpa) and ISNS (Jim Bonham). Testing for 32 metabolic diseases continues through paid testing from parents. A common position paper on NBS in Romania of all the stakeholders have been prepared and will be launched on 19th March. A new Centre of Expertise for metabolic congenital anomalies in Bucharest is under evaluation process. Funding for enlargement of NBS are included in the Operational Program for Health and National Strategy for Health 2023–2030.

I22. **Neonatal Screening in Bulgaria—On Ongoing Challenge**
Iva Stoeva, Iskra Modeva, Violeta Kozhuharova, Zoya Todorova, Neliya Kozhuharova and Tsvetoslav Petrov
University Pediatric Hospital Prof. Ivan Mitev, Neonatal Screening and Functional Endocrine Diagnostics, Sofia, Bulgaria

Neonatal Screening (NS) started in 1978 with a pilot study for PKU at Maternity Hospital Sofia, followed by galactosemia (discontinued 1993). The existing PKU logistic was used to implement in 1993 primary congenital hypothyroidism (NTSH Delfia, Bulgarian-Swiss project) at the University Pediatric Hospital in Sofia as the second screening center covering all NB for CH and later CAH, incl. confirmation, treatment start, follow up, education (screened NB for CH 1954418, CAH 883886 till 31.12.24). Improvement of filter paper quality, NB participation, pre-, analytical; post-analytical indicators became a constant necessity to reach the goals of an effective preventive public health program, regardless of profound health care challenges. Addition of a more frequent disease to the existing NS lead to recognizing NS as a priority among neonatologists. Besides prevention of mental disabilities due to CH, the NTSH program was adapted and used as a tool to monitor iodine sufficiency (declared 2007, ICCIDD). Molecular genetic studies started in 1997 as different primary CH forms and raising incidence (1993 1:3600 vs. 2024 1: 1480) were evident. In 2010 (pilot 2003/04) the NS for classical CAH was implemented (National RD Plan), followed by introduction of the CYP21A2 analysis and molecular characterization of nearly all newborns picked up by NS. The 21 OHD incidence showed annual fluctuations, average 1: 13,810 for 2010–2024. The screening center is involved in the development of national screening-, diagnosis-, treatment guidelines based on ESPE; ISNS consensus statements; own experience. Future activities for 2025/26 are connected with the IRT/PAP screening for CF (IRT Pilot 2014), steroid profiling as 2nd CAH tier, program evaluation.

I23. **Two Years of Expanded Neonatal Screening in Ukraine: Success Despite the Challenges**
Natalia Olkhovych ^1^, Natalia Samonenko ^1^, Halina Makuch ^2^, Mykola Veropotvelian ^3^, Olena Grechanina ^4^ and Natalia Gorovenko ^1^

^1^  Kyiv Regional Center of Neonatal Screening, Expert Center of Neonatal Screening, National Children’s Hospital Okhmatdyt, Kyiv, Ukraine
^2^  Lviv Regional Center of Neonatal Screening, Lviv, Ukraine
^3^  Kryvyi Rih Regional Center of Neonatal Screening, Kryvyi Rih, Ukraine
^4^  Kharkiv Regional Center of Neonatal Screening, Kharkiv, Ukraine
^5^  National Scientific Centre, Institute of Genetic and Regenerative Medicine, Kyiv, Ukraine

Newborn screening (NBS) is a public health program for early detection and management of certain inherited diseases. Despite the challenges posed by the ongoing war, Ukraine set up an expanded NBS in 2022 that includes 21 congenital disorders (CD). Today NBS in Ukraine covers all unoccupied regions of Ukraine. DBS, LC-MS/MS, RT-PCR, IFA, GC-MS, NGS, Sanger sequencing, MLPA. The new system of expanded NBS in Ukraine is conducted by 4 screening centers (SC). It was implemented in two steps: pilot project started in Lviv and Kyiv SC (October 2022) and then Kharkiv and Kryvyi Rih SC joined (April 2023). Today the system is nationwide, OPT IN, free access, and funded by the Ukrainian central body of the healthcare system (National Health Service of Ukraine). Between October 2022 and October 2024, a total of 354 696 newborns were screened with a consent rate of 98%. The general screened population (excl. sick and preterm newborns) had their first blood sampling at the median age of 2 days (IQR 2.0–2.9) and confirmation at the median of 42 days (IQR 29–57). The median time between birth and the first result (turn-around time) was 9 days (IQR 6.7–13). Clinical support and management of first-tier positive results are provided by a regional network of NBS coordinators. The diagnosis is confirmed centrally by an expert screening center. A total of 359 newborns with CD were confirmed. The total incidence rate of screened CD in Ukraine is 1 in 988. False-negative results were reported for two congenital hypothyroidism cases. All babies with confirmed diagnoses have specialized clinical support with multidisciplinary teams in appropriate Reference centers. All babies who need therapy receive it free of charge. The implementation of Expanded NBS in Ukraine highlights improving orphan patients’ medical care by early diagnosis and treatment.

I24. **Challenges of Newborn Screening Expansion in Lithuania**
Jurgita Songailiene ^1^, Elena Urbstaite-Zove ^1^, Marija Smirnova ^1^ and Algirdas Utkus ^2^

^1^  Laboratory of Newborn screening and Diagnostics of IEM, Centre for Medical Genetics, Vilnius University Hospital Santaros Klinikos, Vilnius, Lithuania
^2^  Centre for Medical Genetics, Vilnius University Hospital Santaros Klinikos, Vilnius, Lithuania

Lithuania, located in northeastern Europe, with a population of approximately 2.89 million. In 2024, the country recorded a birth rate of 9.659 births per 1000 people, reflecting a 1.81% decline from the previous year. In 2025, Lithuania will celebrate the 50th anniversary of its NBS program, which commenced in 1975 with the screening for phenylketonuria (PKU). The program has evolved significantly over the decades, incorporating additional conditions including congenital hypothyroidism (CH) in 1993, and galactosemia (GAL) and adrenogenital syndrome (AGS) in 2015. In 2023, eight rare disorders were added to the NBS panel, bringing the total to 12 diseases currently screened: PKU, CH, GAL, AGS, CUD, MCADD, LCHADD, MSUD, MMA, GA1, CF, SMA. The annual coverage of NBS in Lithuania ranges between 99.3% and 99.7%. Although participation in the NBS is not compulsory, parents or guardians who wish to opt out of the NBS must sign the refusal form. The annual refusal rate varies between 0.3% and 0.5%. Since 1975, when the NBS was launched, a total of 1,894,509 newborns have been tested and 452 diagnoses have been confirmed. NBS is free of charge and is reimbursed by the Compulsory Health Insurance Fund. Although the Personal Health Services Assessment Committee is responsible for taking decisions on the extension of the NBS panel, the inclusion of specific diseases reflects a complex interplay of factors. These include scientific evidence on the prevalence, morbidity, mortality and effectiveness of early intervention for the diseases, as well as financial considerations in the healthcare budget. Furthermore, community-driven initiatives emerged from advocacy groups, parent organizations, and public health entities, highlighting the critical role of social engagement in the NBS decision-making process. The inclusion of six metabolic disorders (MS/MS) in the NBS panel, primarily grounded in scientific rationale, extended over a decade. In contrast, the inclusion of cystic fibrosis and spinal muscular atrophy was achieved in a significantly shorter timeframe of one to two years, expedited by intensive advocacy efforts characterized by mass media campaigns that enhanced public awareness and underscored the urgency of these conditions. This case exemplifies the dynamic interplay between scientific evidence and community advocacy in shaping public health initiatives.

I25. **National and Pilot Screening Programmes in Estonia**
Katrin Õunap ^1,2^, Karit Reinson ^1,2^, Kai Muru ^1,2^, Elerin Albin ^1^ and Tiina Kahre ^1,2^

^1^  Genetic and Personalized Medicine Clinic, Tartu University Hospital, Tartu, Estonia
^2^  Department of Genetic and Personalized Medicine, Institute of Clinical Medicine, University of Tartu, Tartu, Estonia

Estonia belongs to the Baltic countries, and its population is approximately 1.3 million. The first pilot study for newborn screening was started in 1993 to diagnose phenylketonuria. In 1996, the newborn screening for congenital hypothyroidism was added to the newborn screening. At the same time, the Ministry of Social Affairs established a newborn screening laboratory at the Tartu University Hospital (TUH) and this program became a public service. During the first ten years, we reached the coverage of all newborns included in the screening over 99%. A second pilot study was initiated in 2014 on a research basis. Amino acids and acylcarnitine analysis from dried blood spots were performed on a Xevo TQD Triple Quadrupole Mass Spectrometer. Six aminocidurias, four organic acidurias, vitamin B12 deficiency and eight fatty acid beta-oxidation defects were selected for the study protocol. During the extended newborn screening program (41,453 newborns 2014–2016), the diagnosed disease prevalence was 1:1884. The Estonian Health Insurance Fund added the amino acids and acylcarnitine analysis to the public screening program in 2015. A third pilot screening program for classical galactosemia was initiated in 2015 but was approved as a public screening program only five years later. Our team has, during the last few years, also initiated the pilot screening for spinal muscular atrophy (in 2022, approved in 2024), for cystic fibrosis (in 2023, not approved yet) and planned to initiate for congenital immune deficiency (in 2025). The main challenge is that all pilot projects have been funded by research projects, charity foundations (Children’s Fund at TUH), and the Genetic and Personalized Medicine Clinic at TUH. The Estonian government does not have a development plan for newborn screening and has not provided financial support to develop new newborn pilot screening programs.

**Funding**: PRG471, PRG2020

I26. **Neonatal screening in the Republic of Kazakhstan: Current State and Prospects For Further Development**
Damilya Salimbayeva and Gulfairuz Urazbayeva
Scientific Center of Obstetrics, Gynecology and Perinatology, Almaty, Kazakhstan

Newborn screening (NBS) for congenital hypothyroidism and phenylketonuria was established in 2007 year in Kazakhstan. It is the national program that covers all the regions of Kazakhstan and fully financed by the government. The algorithm of neonatal screening in Kazakhstan includes international recommendation (ISNS) and use immunofluorescence method with standard kits, uniform certified equipment (in some large regions with high birth rates for Kazakhstan the automated equipment for NBS), reagents in all regional screening departments, uniform NBS algorithm of ISNS. Also regular monitoring of efficiency of NBS such as coverage, timing of diagnosis, initiation of treatment and external quality assurance program NBS (CDC), mandatory annual trainings and certification all regional NBS specialists. Thus, a coordinated and comprehensive NBS system has been organized in Kazakhstan, including training, screening, follow-up, diagnosis, treatment and management, and program evaluation. Blood for NBS is taken from all newborns between 24 and 72 h after birth in maternity hospitals, then bloodsports transport to regional screening departments and results of NBS should be ready after 3 days. Newborn with a positive NBS results should be invite to a regional screening department for knowledge and diagnostic procedures and early treatment. The treatment should be initiated before 21 days of life. This algorithm of NBS is more useful and successful for Kazakhstan considering legal issues and national health policy. Prospects are the expansion of nosologies for mass NBS—congenital dysfunction of the adrenal cortex, biotinidase deficiency, cystic fibrosis, spinal muscular atrophy, primary immunodeficiencies, as well as the introduction of new methods for neonatal screening—tandem mass spectrometry, molecular genetic methods. But for this, it is necessary to conduct pilot studies taking into account the characteristics of the population of Kazakhstan and the capabilities of the healthcare system.

I27. **ELSI Implications of Genomic NBS for Each Step of the Programme**
Věra Frankova ^1,2^

^1^  Institute for Medical Humanities, Charles University-First Faculty of Medicine, Prague
^2^  Department of Pediatrics and Inherited Metabolic Disorders, Charles University-First Faculty of Medicine and General University Hospital in Prague

The integration of genome sequencing into the newborn screening (NBS) pro-gramme is anticipated to impose considerable demands on the entire healthcare sys-tem and generate a range of ethical, legal and social implications (ELSI). These issues can be evaluated in the overall context of the benefits and harms associated with ge-nome-based NBS. However, these ELSI considerations are also relevant to each phase of the NBS programme and expected to pose significant challenges. The primary con-cerns pertain to the selection of appropriate diseases for screening in newborns as well as the long-term storage and subsequent reuse of individual genomic data, both for diagnostic purposes and research. In the pre-analytical phase, it is crucial to ensure that parents are adequately informed and provide informed consent for both tradi-tional and genome-based NBS. It is unclear whether the long-term storage of genomic data and their further diagnostic and/or secondary use will be performed in an opt-in or opt-out mode. In the subsequent analytical phase, it is important to establish clear criteria for negative and positive findings to avoid the unnecessary medicalisation of children and their families. In the post-analytic phase, it is essential to ensure multi-disciplinary care and psychosocial support for families of children who are identified as positive cases and to guarantee equal access to treatment. Secure data storage is paramount for safeguarding the privacy of children and their families, with particular emphasis on the vulnerability of minors. Prior to the integration of genomic sequenc-ing into the NBS program, it is critical to engage all relevant stakeholders in a com-prehensive discourse and address these ELSI aspects to maintain public trust in NBS programmes.

This work has been supported by MŠMT project BBMRI_CZ LM2023033

I28. **Psychosocial Communication between Doctors, Dietitians, and Patients/Parents: Initial Contact, Challenges, and Troubleshooting in the Context of the Greek National Newborn Screening Program**
  Eleana Petropoulou
Institute of Child Health, Department of Newborn Screening, Thivon & Papadiamantopoulou street, Athens Greece

Psychosocial communication between healthcare professionals, such as doctors, dietitians, and their patients or parents, plays a crucial role in ensuring effective care and positive health outcomes. The initial contact between a doctor or dietitian and a patient or their family often sets the tone for the therapeutic relationship, influencing trust, engagement, and adherence to treatment plans. Effective communication is essential in addressing not only the medical or nutritional needs but also the emotional, psychological, and social aspects of care. Challenges arise from varying levels of health literacy, cultural differences, emotional distress, and diverse expectations. Misunderstandings or lack of clear communication can lead to non-compliance, frustration, and stress. Parents of a newborn need continuing support and help as these first days of a <working diagnosis> are the most difficult. To troubleshoot these challenges, professionals must develop skills in active listening, empathy, and tailored explanations. Establishing rapport, setting realistic expectations, and providing support for emotional and practical concerns can strengthen the doctor-patient and dietitian-patient/parent relationship. Addressing psychosocial barriers early on, along with continuous reassessment, can improve patient satisfaction, foster collaboration, and ultimately enhance care effectiveness.

I would like to acknowledge the clinical team of the Greek Newborn Screening Program: Dr. Anastasia Skouma (Metabolic Paediatrician), Dr. Triantafyllia Sdogou (Metabolic Paediatrician), Mr. Kostas Iakovou (Metabolic Consultant).

I29. **Cyber Attack on Irish Health Service ICT System 2021 and Impact on Newborn Screening Service**
Loretta O’Grady
Newborn Bloodspot Screening Laboratory, Department of Paediatric Laboratory Medicine, Children’s Health Ireland, Temple Street, Dublin

In the early hours of Friday 14 May 2021, the National Health Network in Ireland was subjected to a serious malware attack through the criminal infiltration of their IT systems using Conti ransomware impacting critical IT infrastructure. NBS LIMS like all systems linked to that one major national site were shut down. Critical Incident process was invoked, which began a sequence of events leading to the decision to switch off all Health IT systems and disconnect the National Healthcare Network from the internet in order to attempt to contain and assess the impact of the attack and removed the Attackers access to the health service environment. This immediately resulted in healthcare professionals losing access to all Health provided IT systems—including patient information systems, clinical care systems and laboratory systems. Normal communication channels were immediately lost; email, networked phone lines etc. The aim of the Attacker was to disrupt health services and IT systems, steal data, and demand a ransom for the non-publication of stolen data and provision of a tool to restore access to systems. The Attacker posted a message on an internet chat room on the dark web, with a link to several samples of data reportedly stolen. The Incident had a far greater and more protracted impact than initially expected, with recovery efforts continuing for over four months.

I30. **Safeguarding Newborn Screening Services against Cyber Attacks: A Case Study in Resilience**
Rachelle Garstone
South East Thames Newborn Screening Laboratory (Synnovis)

On 3 June 2024, Synnovis suffered a ransomware attack, resulting in the loss of access to key IT systems, including OMNILab, the Laboratory Information Management System (LIMS) used for newborn screening (NBS) at the South East Thames Newborn Screening Laboratory. Without access to this critical system, the laboratory had to rapidly develop and implement alternative workflows to ensure the continuation of newborn screening services. To mitigate the impact of the outage, a manual reporting process was established using Microsoft Excel, accessible via the hospital Trust’s IT infrastructure. This interim workflow supported essential functions, including specimen tracking, result and internal quality control (IQC) review, demographic verification, retest management, referral coordination, and outcome reporting to the Newborn Blood Spot Failsafe Solution (NBSFS) and Child Health Information Systems (CHIS), thus enabling the laboratory to continue reviewing and reporting NBS results. Access to the LIMS was restored 135 days later. The response required close collaboration between the NBS laboratory, Synnovis IT, and WatersCorp (the OMNILab provider) to restore a backup of the LIMS and repatriate data generated during the period of downtime. Importantly, the laboratory performed a retrospective review which was significant in assessing the impact of IT disruptions and for strengthening future resilience, including improvements to future contingency planning. As healthcare systems become increasingly digitised, laboratories are vulnerable to cybersecurity threats with the potential to disrupt operations and compromise patient safety. This case study summarises the key adaptations made to workflows and reporting processes in response to such challenges, and the lessons learned. Our experience reinforces the need for robust preparedness strategies to protect essential newborn screening services and ensure uninterrupted care in an increasingly digital healthcare landscape.

**Acknowledgments**: Rachel Carling, Director Newborn Screening and the Newborn Screening Laboratory Team.

I31. **The Newborn Screening Laboratory and Cyber Attacks: Response to a Customer’s Loss of Access to OMNI-Lab: Support and Solutions**
Ian Hutton
Waters Corporation

In June 2024, a Newborn Bloodspot Screening customer experienced a disruption to their entire IT infrastructure, including OMNI-Lab, with no clear timeline for restoration. As a result, the customer had to implement alternative methods to maintain service continuity. In the initial weeks, critical decisions had to be made regarding the hosting of OMNI-Lab: the appropriate version of OMNI-Lab to use, access to both users and the Waters support team, the process for importing sample data once access was restored, and whether database encryption should be implemented. Throughout this period, the Waters Support team participated in daily meetings, providing expert guidance and essential information to assist the customer. Ultimately, OMNI-Lab access was successfully reinstated, and sample data collected during the downtime was efficiently imported. The customer expressed high satisfaction with the level of support provided by Waters.

I32. **The Newborn Screening Laboratory and Cyber Attacks: Era after the Walls**
Tommi Elo
Revvity

Cybercrime has become the largest illicit business in the world, offering immense rewards with minimal risk of prosecution. Newborn screening programs are vulnerable unless they proactively protect themselves against these new threats. Traditional security measures, such as relying on physical or virtual firewalls, are no longer sufficient. As digital systems expand, vulnerabilities increase, making it challenging to maintain a secure perimeter. The latest cyber threats require each system to protect itself, with access based on user roles and strong identification rather than network location. This approach ensures that only authorized users can access critical systems and sensitive data. Modern problems require modern solutions. Advanced information systems are built from the ground up with security in mind, protecting data through six key pillars: access management, encryption, data loss prevention, automated updates, backup and disaster recovery, and certification and testing. Together, these pillars significantly enhance the cyber resilience of information systems. Furthermore, as these systems are no longer dependent on physical perimeters, they enable the benefits of cloud computing, such as accessibility, scalability, and low cost of ownership. However, technology alone is insufficient. Human factors often weaken security, necessitating proactive organizational measures. These include educating staff on cybersecurity best practices, implementing strong policies, conducting regular audits, investing in robust platforms, and staying informed about emerging threats. Even small steps can make a significant difference, as seen in other industries where improved security focus has shifted criminal attention elsewhere. In conclusion, cybersecurity is a shared responsibility requiring constant vigilance. By prioritizing cybersecurity, we can protect the integrity and confidentiality of data in screening laboratories, ensuring the credibility of newborn screening in the future.

I33. **Newborn screening for Remethylation Disorders: the State of the Art**
Giancarlo la Marca ^1,2^

^1^  Newborn Screening, Clinical Biochemistry and Clinical Pharmacy Lab, Meyer Children’s Hospital IRCCS, Florence, Italy
^2^  Department of Experimental and Clinical Biomedical Sciences, University of Florence

Tandem mass spectrometry (MS/MS) has become a leading technology used in clinical chemistry and it has shown to be particularly sensitive and specific when used in newborn screening (NBS) tests. The success of tandem mass spectrometry is due to important advances in hardware, software and clinical applications during the last 30 years. MS/MS permits a very rapid measurement of many metabolites in different biological specimens by using filter paper spots or directly on biological fluids. Its use in NBS give the chance to identify possible treatable metabolic disorders even when asymptomatic and the benefits gained by this type of screening is now recognized worldwide (1). Total homocysteine (Hcy) is the elective primary biomarker for detecting remethylation disorders (RD) (2) but only recently it has been introduced as NBS first-tier test (3). There are several commonly used biomarkers for RD detection: propionylcarnitine (C3), Methionine (Met); C3/C2 and C3/Met, Met/Phe ratios. The sensitivity of C3/C2 as primary marker has been reported as superior to Met and Met/Phe for RD-early onset forms (4). On the contrary, the sensitivity of C3 and C3/C2 for mild/late-onset forms remains unknown (2). Moreover, neonatal metabolic disturbances due to maternal vitamin B12 deficiency may also be detected (2,5). The literature confirmed that the specificity of those biomarkers for detecting RD was generally low, although the introduction of free methylmalonic acid (MMA) and Hcy as second tier biomarkers strongly increased the overall positive predictive value (PPV) (6,7). Heptadecanoyl-carnitine (C17) may have an even higher predictive value for perturbations of MMA metabolism including combined RD and may thus be a promising first tier analyte (5). We present combined multiple- tier strategies as working practice to achieve the most benefit out of newborn screening programs for RD.

(1) PMID: 25952022

(2) PMID: 27905001

(3) PMID: 36920064

(4) PMID: 21325949

(5) PMID: 26368264

(6) PMID: 17510301

(7) PMID: 20807894

I34. **The Dutch Laboratory Experience of Recent NBS Programme Expansions—Sharing Key Insights and Knowledge Gained**
Rose Maase ^1^, Marelle Bouva ^1^, Wouter Visser ^1^, Els Voorhoeve ^1^, Marie-Louise ^2^ Heijnen and Eugenie Dekkers ^2^

^1^  Department Public health genomics & Screening (PGS), Centre for Health Protection (GZB), Dutch National Institute for Public Health and the Environment (RIVM)
^2^  Centre for Population Studies (CvB), Dutch National Institute for Public Health and the Environment (RIVM)

The Dutch Newborn Screening (NBS) was established in 1974 with screening for PKU and in the last 50 years it has expanded to include 27 conditions. The most recent significant, phased expansion was initiated following advice from the Dutch Health Council in 2015: between 2019 and 2023 eight conditions were added (PA, MMA, CPT1, GALK, SCID, MPS I, SMA, and ALD). Each expansion was unique, both in terms of preparation and the outcomes of the screening in practice. Specific examples from these recent NBS-programme expansions illustrate what went well—where the involvement of multidisciplinary expertise, planning and good consideration paid off—and the (unforeseen) challenges that were faced, which provided learnings for subsequent programme expansions. Specifically, insights will be shared in screening algorithm design, target disease definition, integration of new conditions in an existing (complex) NBS-programme, implementation process, uniformity of follow-up, post implementation monitoring and processes in both the short and long term and screening optimalisations.

I35. **Experiences from the First Prospective Newborn Screening for Metachromatic Leukodystrophy (MLD) in Germany**
Lucia Laugwitz and Samuel Gröschel
Department for Neuropediatrics, University of Tübingen, Germany

The first pilot study on newborn screening (NBS) for MLD in Tübingen, Germany, demonstrated the feasibility and effectiveness of early detection and intervention. Conducted between October 2021 and July 2023, the study screened 109,259 newborns using a three-tiered strategy. The first tier involved sulfatide screening, followed by aryl sulphatase A enzyme activity measurement and genetic sequencing in cases with elevated sulfatide levels. Three newborns were identified as screen positives and MLD was confirmed subsequently in all three cases. Based on genotype and enzyme activity, two infants were predicted to have early-onset MLD and received autologous hematopoietic stem-cell gene therapy, Atidarsagene autotemcel (arsa-cel), at 12 months. The third, with predicted late-onset MLD, remained under medical surveillance for potential future allogeneic hematopoietic stem-cell transplantation (HSCT). At follow-up, all three children showed normal developmental progress. This pilot study highlights the potential of high-throughput NBS for MLD, enabling presymptomatic diagnosis and treatment. It establishes a foundation for integrating MLD screening into routine newborn programs, which could significantly improve long-term outcomes by preventing irreversible neurodegeneration before symptom onset.

I36. **Newborn Screening for Adrenoleukodystrophy and The Impact of Variants of Uncertain Significance**
Kelly Miettunen ^1^, Marc Engelen ^2^, Troy Lund ^3^ and Stephan Kemp ^4^

^1^  ALD Connect (https://aldconnect.org/)
^2^  Department of Pediatric Neurology, Emma Children’s Hospital, Amsterdam UMC, Amsterdam Leukodystrophy Center, Amsterdam Neuroscience, University of Amsterdam, Amsterdam, The Netherlands
^3^  Department of Pediatrics, Blood and Marrow Transplant Program, University of Minnesota Medical School, Minneapolis, MN, USA
^4^  Laboratory Genetic Metabolic Diseases, Department of Laboratory Medicine, Amsterdam UMC, Amsterdam Gastroenterology Endocrinology Metabolism, University of Amsterdam, Amsterdam, The Netherlands

Males with X-linked adrenoleukodystrophy (ALD) are at high risk for developing adrenal insufficiency and/or progressive leukodystrophy (cerebral ALD) at an early age. Pathogenic variants in *ABCD1* result in elevated levels of very long-chain fatty acids (VLCFA), including C26:0-lysophosphatidylcholine (C26:0-LPC). Newborn screening for ALD has been implemented in most US states, Taiwan, and the Netherlands, with pilot programs in several other countries. This screening enables prospective monitoring and timely therapeutic intervention, potentially preventing irreversible damage and saving lives. However, current screening protocols face challenges. The C26:0-LPC cutoff is set to avoid false negatives, but this approach identifies many newborns with levels above the cutoff yet below the range seen in clinically affected patients—a scenario referred to as the “Grey Zone”. Many of these cases involve *ABCD1* variants not previously reported in known patients (see *ABCD1* Variant Registry: https://adrenoleukodystrophy.info), classified as variants of unknown significance (VUS). In fact, the likelihood of identifying a VUS in *ABCD1* is three times higher in ALD newborn screening compared to clinical testing. This situation raises important questions about the current screening approach. It’s unclear whether all identified boys will develop symptoms, yet most undergo rigorous follow-up as recommended by guidelines. The high rate of Grey Zone results and VUS identification suggests that changes to current protocols may be warranted to focus on identifying only those who will truly benefit from early diagnosis and follow-up. Recent observations show a correlation between C26:0-LPC levels and adrenal failure and leukodystrophy. To this end, the non-profit organization ALD Connect has initiated the Grey Zone project, which aims to improve understanding of VUS identified in *ABCD1* and determine their potential pathogenicity. This may allow for increasing C26:0-LPC cutoff levels in screening protocols to avoid identifying non-patients or those with mitigated disease courses who are not the target of newborn screening.

I37. **Adrenoleukodystrophy—A Family Story behind the Need for Screening**
Karen Harrison
Alex, The Leukodystrophy Charity

Karen will share a deeply personal and poignant journey—the story of a mother who faced the heart-wrenching reality of a rare disease diagnosis for her two sons. This narrative is not just hers, but it echoes the experiences of countless families around the world grappling with rare diseases. The journey began with a quest for answers. After numerous consultations and tests, the devastating diagnosis arrived. The impact was profound, altering the lives her family in ways that words can scarcely capture. The knowledge that their condition was rare and due to one son having symptoms, he had missed the opportunity for treatment, her other son had a chance. As she learned more about adrenoleukodystrophy, it became clear that if newborn screening had been available, her older son would be alive today. This simple yet powerful tool could have changed her sons’ fate. Yet, despite the passage of 22 years, little has changed in the UK. A reliable test exists, yet families continue to receive the same grim prognosis following diagnosis, their hopes shattered and futures uncertain. This story is a reminder that the fight for better healthcare and early diagnosis is far from over. It is a plea for action, not just for one family, but for the millions affected by treatable rare diseases. The pain and struggles of these families, from the UK to every corner of the globe, must not be in vain. Let us rally together to advocate for better screening, improved treatments, and, ultimately, hope for a brighter future.

I38. **Screening for MPS1: What Is the Real Life Result?**
Hanka Dekker
Director, VKS, Association for Children with Metabolic Disease, Zwolle, Netherlands

In the Netherlands, we screen newborn babies for 27 diseases of which 19 are serious but treatable metabolic diseases. Since 2021, the lysosomal storage disease MPS1 has been added to the screening programme. The Netherlands is one of the few countries in Europe that conducts this screening. This is important because early detection changes lives. We bring you the story of baby Yann. When he was born, everything seemed normal. The heel prick test showed something abnormal. MPS1 was diagnosed. This is a serious metabolic disease. Without treatment, children with the severe form develop physical and neurological problems. This form, if left untreated, leads to death at a young age. The film will show what his treatment was like and how his parents feel about the heel prick test in hindsight. Yann is not cured; he will need lifelong monitoring. But thanks to just a few drops of blood, he has been given a chance to live fully … and that is priceless say his parents. Up until this year six children with the severe form of MPS1 were found pre-symptomatically via newborn screening in the Netherlands. All were treated with allogeneic hematopoietic stem cell transplantation (allo-HSCT).

I39. **Screen4Rare, the Involvement of ISNS**
James R. Bonham
Sheffield Childrens (NHS) FT, Sheffield, UK

Where it is offered, newborn screening is popular among both parents and professionals and uptake is typically > 99%. Despite this universal popularity, we see a marked variation in the number of conditions tested across Europe ranging from 2–48 disorders. The European Commission, keen to maintain national autonomy in the area of Public Health, is reluctant to mandate or issue guidance in this important policy area. In order to make progress, Screen4Rare, launched by the International Patient Organisation for Primary Immunodeficiencies (IPOPI), the International Society for Neonatal Screening (ISNS), and the European Society for Immunodeficiencies (ESID) has sought to work with MEPs via an MEP Alliance and to support policy makers, particularly in Europe to work towards ensuring that all babies can have equitable access to newborn screening. Crucial to this are the involvement of the relevant European Reference Networks (ERNs) who care for the patients with disorders commonly diagnosed by newborn screening. Screen4Rare seeks to influence policy and practice through discussion and the provision of unbiased scientific information, evidence, and comparative data to help ensure that good decisions are made on behalf of the populations served. The potential development of gNBS expanding the range of potential target disorders to several hundred highlights the importance of this work. Three key areas for the development of evidence and information include:

- Workstream 1 Developing a blueprint for screening
- Workstream 2 Case definitions
- Workstream 3 Data-driven guidance of a screening strategy for rare diseases

The goal is the development of appropriate, well-organised and equitable newborn screening to help identify well-defined treatable conditions where it is clear that their early asymptomatic detection and treatment during childhood results in significantly improved outcome.

I40. **Workstream 1: A blueprint for Screening in Europe—Identifying Good and Best Screening Practices**
Peter C. J. I. Schielen
International Society for Neonatal Screening, Stichtse Vecht, the Netherlands.

Screen 4 Rare is a multi-stakeholder initiative launched in 2020 by the International Patient Organisation for Primary Immune Deficiencies (IPOPI), the International Society of Neonatal screening (ISNS) and the European Society for Immunodeficiencies (ESID). Early on in its existence, European Reference networks like Metabern ERN-RITA and ERN-NRD have joined to make Screen4Rare a strong collaborative network aimed at exchanging knowledge and best practices on neonatal screening (NBS) for rare diseases. Screen4Rare’s ultimate objective is to ensure all babies born in the EU can have equal access to NBS which can be a life-saving tool for inborn errors of metabolism, inborn immune deficiencies, haemoglobinopathies and other neonatal disorders. Major achievements of Screen4Rare are the organization of meetings before members of the European Parliament where Screen4Rare’s ‘Call to Action’ was presented, namely to (1) find common ground for generally accepted overarching guidelines, (2) build a platform for stakeholders and (3) cooperate to position the EU as the central point for data collection and information on NBS practices. The call to action is endorsed meanwhile endorsed by many members of parliament. Early in 2022 the action points were developed into tangible aims, introducing three workstreams, namely to (1) develop a blueprint for best practice to guide neonatal screening in Europe, (2) develop agreed case definitions and confirmatory testing for European screening and (3) support the development of interoperable disease registries for screened conditions within Europe. In this presentation the aims of Workstream 1 are laid out, more specifically to

1. Recognize NBS as a system and define the core elements of that system;
2. Identify where standards may be helpful within these core elements and measure indicators to compare to these standards;
3. Picture the current situation of all indicators based on vitally important information gathered by colleagues in Europe;
4. Understand the differences, explain and decide on improvement, and measure again;
5. Build a repository of good and best practices, policies, etc.;
6. Provide a platform for learning and exchange of knowledge, including pilot scheme data.

Additionally, this presentation will address recent developments on some of items 1–6.

I41. **Workstream 2: Who Is the patient? The Importance of Accurate Diagnosis and Outcome Comparisons in Newborn Screening**
Annet M. Bosch
Pediatrician for Metabolic Diseases, Amsterdam University Medical Centers, Amsterdam, The Netherlands

The implementation of extended newborn screening programs over the past few decades has resulted in a significantly different phenotypic spectrum for many inherited metabolic diseases. Previously, clinical presentations demonstrated the “classical” phenotype of each disease. However, at this time many milder cases are being detected, some of which do not exhibit a phenotype. In these cases, it remains uncertain whether a phenotype will develop over the course of the individual’s lifetime at all. To avoid the burden of a diagnosis that may never lead to a clinical phenotype for children and their families, it is crucial to establish clear case definitions, preferably before introducing a disease into a screening program. Establishing shared case definitions in an international context is essential for two reasons. Firstly, to prevent uncertainties and anxieties in patients and parents that arise from differences in diagnostic and treatment strategies between countries. Secondly, for a valid evaluation of long-term outcomes of diseases included in newborn screening programs, international cooperation is vital. Strict shared case definitions help to ensure that only a defined range of “true positive” phenotypes is included. Examples of case definitions currently being resolved include those for galactosemia and biotinidase deficiency.

I42. **Workstream 3: Data-Driven Guidance of a Screening Strategy for Rare Diseases**
Ulrike Mütze, Florian Gleich, Thomas Opladen, Sven F. Garbade, and Stefan Kölker on behalf of the Unified Registry for Inherited Metabolic Disorders (U-IMD) Consortium, the European Reference Network for Hereditary Metabolic Disorders (MetabERN)
Heidelberg University, Medical Faculty of Heidelberg, Department of Pediatrics I, Division of Pediatric Neurology and Metabolic Medicine, Germany

Newborn screening (NBS) is a highly effective measure of preventive medicine and NBS panels continuously grew since the implementation of NBS for phenylketonuria about 60 years ago. However, despite the screening criteria of Wilson and Jungner and strict national evaluation processes, several aspects regarding the benefit of an NBS remains incompletely known when introducing a new target disease: The incompletely understood natural history, the unreliable prediction of individual health problems and disease courses, the uncertainty about case definitions and confirmatory testing protocols, and an ambiguity concerning indication to treat and the health impact of therapies, and thus, a knowledge gap on the long-term benefit of screened individuals, their families, national health systems, and societies. NBS should, therefore, be aligned by long-term observational studies/patient registries that serve as a key tool to evaluate benefits and harms of NBS and allow to optimize NBS programs in an iterative cycle utilizing real-world evidence. Regional and national registries have been implemented, however, cohorts are often limited in size, therefore European registries were needed. U-IMD the official registry of the European reference Network for Metabolic Disorders (MetabERN) and in 2023 a new NBS model was developed and allows a structured and collaborative evaluation of NBS programs in a realistic manner. This NBS module could be used for a collaborative inter-ERN/transnational evaluation of future NBS programs which will further expand and include a growing number of diseases from various subdisciplines.

I43. **The EU Advisory Committee on Newborn Screening under the Umbrella of the ERNs**
Andrea Bordugo and Maurizio Scarpa
Regional Coordinating Centre, University Hospital ASUFC Udine Italy

Neonatal or Newborn Screening (NBS) is a population based program aimed at the pre-symptomatic detection, shortly after birth, of serious and treatable conditions an so permitting appropriate therapy to prevent long term disability. In recent years with the introduction of tandem mass spectrometry 40–50 conditions can be tested and Next Generation Sequencing (NG) could dramatically increase such a number, with all pro and cons. In spite of the consensus concerning the value of NBS, there are large differences among countries in Europe in different aspects regarding number of diseases tested, time of sampling, parents’ informed consent and referral strategies. In 2017, supported by the Cross Border Directive, 24 European Reference Networks (ERNs) were established in all 27 EU members to promote equity of access to diagnosis, management and treatment for rare diseases in EU. Recently a newly formed collaboration between the European Reference Networks (Including MetabERN for Inherited Metabolic Disorders and RITA for immunodeficiencies, autoinflammatory and autoimmune conditions), the international Society for Neonatal Screening (ISNS), the International Patient Organisation for Primary Immunodeficiencies (IPOPI) and the European Society for Immunodeficiency (ESID), all operating inside the Screen 4 Rare Project is keeping the challenge to stimulate equity in neonatal screening in Europe. The Screen for Rare project has recently issued a Call for Action to recruit support from Health Policy makers and European members of parliament (MEP’s). The aim, together with ERNs, is to develop agreed case definitions and confirmatory testing for European screening, support the development of interoperable disease registries for screened conditions within Europe and develop a blueprint of best practices to guide neonatal screening in Europe. ERNs, as a model of collaboration across EU countries in recent years, could have a crucial role in the future, helping to implement an Action Plan on Rare Diseases and develop an EU-Level NBS Expert Advisory Committee to support and harmonize newborn screening practices across Europe.

I44. **Future Perspective, Next Steps, What Needs to Be Done Next, Forward Looking to 2028—From a Patient Perspective**
Leire Solis
IPOPI, the International Patient Organisation for Primary Immunodeficiencies

A small group of primary immunodeficiencies, the so called SCIDs (severe combined immunodeficiencies), are paediatric emergencies in which the babies are born with no immune system at all. They are asymptomatic at birth, but without early detection and diagnosis and the subsequent effective intervention, the condition is associated with almost 100% mortality. This is the reason why IPOPI is strongly advocating for NBS of treatable diseases, as this is somehow the missing piece in the EU legislation for rare diseases. IPOPI acknowledges and strongly believe that NBS is a comprehensive process that requires a prepared healthcare system to ensure that babies identified through the screening and their families are not left alone but fully taken care throughout the process. The implementation of NBS of SCID and of treatable rare diseases in general is a story of success. There is room for improvement, and this can be done from learning best practices from other countries. In addition, the advent of genomics has the potential to impact how NBS is performed in the near future. Whilst this could potentially lead to a growing number of disparities it also has the potential of bringing many benefits if well implemented. Maintaining public confidence in NBS is of utmost importance and raising awareness about the real-life impact of genomics in medicine will be key. Our vision for 2028 is supporting:

1. Increase awareness and educate countries on the importance of NBS
2. Enhance the support of those European reference Networks for rare diseases with an existing interest in diagnosis through NBS
3. Continue developing trusted sources of information and provide access to advice while respecting national autonomy
4. Work towards the development of good practices that can be used by other countries

In conclusion, collective efforts in advancing NBS for treatable rare diseases are essential for saving lives and improving the quality of life for countless families. IPOPI will continue working to ensure that every baby has the best possible start in life, and so that best practice becomes common practice.

I45. **Life Changing Drugs for Some People with CF; Implications for Newborn Bloodspot Screening**
Kevin W. Southern
University of Liverpool, Institute in the Park, Alder Hey Children’s Hospital, Eaton Road, L12 2AP, Liverpool

The outlook for people with CF has improved steadily over the past 50 years with therapies that address the sequelae of the molecular defect, most notably malnutrition and respiratory disease. Over the past 15 years, new therapies have emerged that directly correct the molecular defect. These CFTR modulator therapies have had a dramatic impact on people with CF, improving clinical outcomes and well-being. In this talk, I will review the development of these agents and the expanding CFTR modulator programme. Many people with CF can now access and benefit from these therapies. They are becoming available for younger age groups (down to a few months for some agents) and the importance of early recognition through NBS has been thrown into even sharper perspective. Some countries in Europe, e.g., Sweden and Finland, still do not consider NBS for cystic fibrosis to be justified. In view of the emerging evidence base, this argument has been weak for some time and can no longer be justified. The emergence of CFTR modulator therapy provides a compelling argument for global NBS for CF, even in countries with relatively low CF incidence, for example sub-Saharan Africa and Southeast Asia. This needs to be balanced against access for these high-cost therapies and the likelihood of increased CFSPID recognition in these populations. Finally, it is important to reflect on the challenge of NBS for CF, when an infant has been exposed to CFTR modulator therapy in utero and postnatally through breast milk, with the possibility of false negative NBS tests. NBS programmes and adult CF services need to be aware of this problem and put strategies in place both for the monitoring of these infants and the exclusion of a CF diagnosis.

I46. **Neonatal Screening for CF in Europe: Different Challenges Require Different Algorithms—Is PAP the Solution to All?**
Olaf Sommerburg
Division of Paediatric Pulmonology & Allergology and Cystic Fibrosis Centre, Department of Paediatrics III, University of Heidelberg, Im Neuenheimer Feld 430, D-69120 Heidelberg, Germany

Genetically diverse populations and different healthcare systems in Europe have led to the development of different algorithms for newborn screening for cystic fibrosis (CF-NBS). Immunoreactive trypsinogen (IRT) is always used as the primary screening marker, but the type of second and third test varies widely. Over the last 20 years, pancreatitis-associated protein (PAP) has emerged as an alternative biochemical marker, raising the question of whether PAP could be a universal solution for the diverse populations in Europe. The advantage of using PAP instead of a genetic approach in CF NBS programmes is that it detects fewer healthy carriers and newborns with a positive CF screening result who cannot be definitively diagnosed with CF (CFSPID). Several European countries have therefore included PAP in their screening algorithms, albeit with different combinations of IRT, PAP, DNA analysis and safety net strategies. However, the typical problems of some PAP-based algorithms, such as lower sensitivity and a relatively low positive predictive value compared to protocols with DNA analysis, have not yet been resolved in all screening programs. Hybrid protocols that combine IRT, PAP, and DNA analysis in different ways appear to provide an optimal balance. For example, the Netherlands uses an IRT/PAP/DNA/EGA approach that has high sensitivity while minimizing the detection of CF carriers. On the other hand, Austria uses a sophisticated IRT/PAP/IRT protocol with multiple cut-offs and an IRT-dependent safety net, which also seems to achieve sufficient sensitivity. It can therefore be concluded that PAP analysis can be a valuable component of CF-NBS, especially when extensive DNA analysis in ethnically diverse populations is not possible for various reasons. However, it is unlikely that PAP can serve as a universal single solution at a time when early knowledge of disease-causing *CFTR* variants is playing an increasingly important role in the management of CF.

I47. **Impact of Increasing Ethnic Diversity on the UK CF Screening Algorithm**
Toby Greenfield
Blood Sciences Department, Portsmouth Hospitals Trust

Newborn screening for CF was rolled out across the whole of the UK in 2007. The UK protocol consists of an initial IRT which, if elevated, is followed up by a DNA panel comprised of the 4 commonest mutations known to cause CF in the UK. If indicated this can be followed up with more extensive DNA testing and a repeat IRT. On commencing screening for CF, it became apparent that the IRT assay displayed marked lot to lot variability. Differences were found between laboratories and also between the different analyser platforms, namely the AutoDelfia and GSP, both provided by Revvity. As of January 2025 the cut-off used for the AutoDelfia labs is 65 ug/L and for the GSP it is 58 ug/L. The 99.5th centile is used as the IRT cut-off and in 2007 this was set at 70 ug/L. Since then, this cut-off has been regularly adjusted and has ranged from 55–70 ug/L. In 2020 a specific IRT data group was established to monitor IRT cut-offs based upon quarterly data collected. Where possible, ethnic data was collected alongside the IRT data. It was felt that ethnicity was potentially a major factor impacting IRT variation and CF outcome data hinted that the positive predictive value of CF screening varied between ethnicities. Ethnicity data was collected for 4 years, and it was found that the IRT 99.5th centile was very different for different ethnicities. Assessment of 10 years’ worth of data from 5 UK labs indicated that CF outcomes was influenced by ethnicity. Exactly how best to apply these findings to CF screening is complex, it may be simplest to make clinicians aware of this data so families can be counselled appropriately.

Acknowledgements: Catherine Collingwood, Maya Desai, Lesley Tetlow, Jim Bonham, Laura Wainwright and all the UK Labs that have provided data to assist with this work.

I48. **Challenges of Providing Genetic Testing for CF in a Small Country**
Marizela Kulisic ^1^, Patricia Borde ^2^, Abdelkader Heddar ^1^, Clément Kebbabi ^2^, Anna-Maria Charatsi ^3,4^, Isabel de la Fuente Garcia ^3^, Thierry Streng ^1^, Frank Goetzinger ^2^, Valery Leduc ^1^, Héléna Marchaix ^1^ and Daniel Stieber ^1^

^1^  National Center of Genetics, LNS, Laboratoire national de santé, Luxembourg
^2^  Department of Medical Biology, LNS, Laboratoire national de santé, Luxembourg
^3^  Paediatric Department, Kannerklinik, Centre Hospitalier de Luxembourg
^4^  Pneumology Department, Centre Hospitalier de Luxembourg

Newborn screening (NBS) for cystic fibrosis (CF) in Luxembourg was introduced in 1 January 2018. The Luxembourgish protocol for CF NBS relies on Immunoreactive Trypsinogen (IRT) on day 3 of life (D3) as the primary test (Autodelfia Neonatal IRT kit, Revvity). DNA analysis of *CFTR* gene variants is performed in case of high IRT (>60 ng/mL). The Yourgene^®^ Cystic Fibrosis Base (IVDR) panel which includes 50 pathogenic variants and 4 polymorphisms of the *CFTR* gene is used for genetic testing. A safety net based on IRT on day 21 was introduced in June 2022. Patients screened positive are referred to the Paediatric National CF center where sweat test is performed. Genetic counselling is provided to all patients with pathologic genetic tests. IRT was above the threshold level in 1.16% of live births. CF was diagnosed in 14 infants so far. All patients had at least one *CFTR* variant identified by the genetic panel. Identification of the second variant with sequencing of the whole *CFTR* gene was required in 4 cases (4382delA, E664X, I502T, S489L). No missed cases have been identified to date. All CF patients were seen by a CF specialist within the first month of life. Since the population in Luxembourg is multi-ethnic, selection of the appropriate genetic test for neonatal screening was challenging. The decision making process was presented on European Cystic Fibrosis Society meeting in 2018 (DOI:10.1016/S1569-1993(18)30310-2). The Luxemburgish protocol was changed twice until now, in June 2019 when Luminex TAG CFTR 71 v2 panel (70 *CFTR* variants) was replaced by Yourgene (ex- Elucigen CF-EU2v1) panel (50 CFTR variants), and in June 2022 when cases with no *CFTR* variants and IRT-D3 < 100 ng/mL were considered being screened negative. On this way, 44.3% less negative cases were referred for the sweat test and the positive predicted value (PPV) was increased to 26%.

I49. **Swiss CF Register: Improving Performance through Careful Evaluation of Outcomes**
Dr. Jürg Barben
Coordinator, ECFS Neonatal Screening Working Group; President Task Force Neonatal CF Screening; Swiss Working Group for Cystic Fibrosis (SWGCF); Head, Division of Paediatric Pulmonology & CF Centre; Children’s Hospital of Eastern Switzerland, Claudiusstr. 6, 9006 St. Gallen, Switzerland

The main goal of NBS programmes for CF is to detect infants with a treatable disease early to initiate treatment, prevent symptoms, and decrease mortality. While CF-NBS programmes have benefits for children with CF, it is important to be aware of possible harmful effects, e.g., false positives, unclear diagnosis (CRMS/CFSPID). The performance of a CF-NBS should be monitored and reported annually using the ECFS defined key outcome parameters to achieve, as a minimum, the ECFS standards (sensitivity ≥ 95%, PPV ≥ 30%) to ensure high sensitivity while unnecessary testing, stress, and overtreatment of healthy children are kept minimal. Since most children with CFSPID remain healthy throughout their childhood and adolescence, the Swiss programme tries to minimize the detection of children with CFSPID, because no early treatment can be offered to them (risk of over-medicalisation). In Switzerland, CF-NBS was introduced in 2011 based on an IRT–DNA algorithm with a second IRT (IRT-2) as safety net if none of the 18 most common CFTR variants in Switzerland were found and IRT-1 was elevated. All data of the Swiss CF-NBS has been collected prospectively by an independent institute since its introduction, providing a unique opportunity for a long-term evaluation. We used this data to analyse the long-term performance of the Swiss CF-NBS (covering 959,006 IRT-1 analyses and 282 CF-children), estimate the optimal cut-offs for IRT-1 and IRT-2, and investigate simulated (hypothetical) scenarios for algorithm optimisation with different cut-offs for IRT-1, IRT-2, and safety net. The Swiss CF-NBS showed excellent sensitivity (96%) but moderate PPV (25%). Optimal IRT-1 and IRT-2 cut-offs were identified at 2.7 (>P99) and 5.9 (>P99.8) z-scores, respectively. Analysis of simulated algorithms showed that removing the safety net from the current algorithm could increase PPV to 30% and eliminate >200 s heel-prick tests per year, while keeping sensitivity at 95%.

## 3. Oral Presentations

### 3.1. Country Report

O01. **Country Report—Czech Republic (CZ)**
Felix Votava ^1^, Petr Chrastina ^2^, Viktor Kožich ^2^, Karolina Pešková ^2^, Tomáš Adam ^3^, David Friedecký ^3^, Eva Hlídková ^3^, Hana Vinohradská ^4^, Monika Hedelová ^1^, Andrea Holubová ^5^, Milan Macek Jr ^5^, Veronika Skalická ^6^, Renata Gaillyová ^7^ and Iveta Valášková ^7^

^1^  Department of Pediatrics, 3rd Faculty of Medicine, Charles University and University Hospital Kralovske Vinohrady Prague, Czech Republic
^2^  Department of Pediatrics and Inherited Metabolic Disorders, 1st Faculty of Medicine, Charles University and General University Hospital Prague, Czech Republic
^3^  Laboratory for Inherited Metabolic Disorders, Faculty of Medicine and Dentistry Palacky University and University Hospital Olomouc, Czech Republic
^4^  Department of Clinical Biochemistry, Faculty of Medicine Masaryk University and University Hospital Brno, Czech Republic.
^5^  Department of Biology and Medical Genetics, 2nd Faculty of Medicine, Charles University and Motol University Hospital Prague, Czech Republic
^6^  Department of Pediatrics, 2nd Faculty of Medicine, Charles University and Motol University Hospital Prague, Czech Republic
^7^  Department of Medical Genetics, Faculty of Medicine Masaryk University and University Hospital Brno, Czech Republic

CZ has 10.5 million inhabitants, birth rate dropped to 85,000 in 2024. A universal insurance-reimbursed programme is governed by the National Screening Center of the Ministry of Health. Blood spots are collected at the age of 48–72 h (99.3% within this timeframe) using Whatman 903 paper. After a median transport time of 3 days DBS are analysed in 6 labs (immunoassay+MS/MS+DNA for Bohemian and Moravian part of CZ, respectively). Twenty primary conditions were determined by the Ministry of Health and include CH, CAH, CF, PKU/HPA, MSUD, GA I, IVA, ARG, CIT, CBS, MTHFR, MCADD, LCHADD, VLCADD, CPTD I and II, CACTD, BTD and SMA+SCID (since I/2024). TSH is used for CH: AutoDelfia^®^, cutoff: ≥15 mIU/L blood. Additional collections: a/after reaching 1500 g in very low birth weight or b/maternal therapy with thyreostatics or high iodine drugs in the last trimester or c/newborn therapy with dopamine, high iodine drugs or blood derivates prior to regular sampling. Incidence 1:2870, specificity 0.9998, PPV 0.6421. Phe and Tyr are used for PKU: MS/MS API, cutoff: Phe ≥ 120 μmol/L and Phe/Tyr ≥ 2,0. Additional collections: newborn therapy with aminoacids, glucose, lipids solutions or blood derivates prior to regular sampling. Incidence 1:4939, specificity 0.9997, PPV 0.4970.

O02. **Country report—Switzerland**
Susanna Sluka
Newborn Screening Switzerland, University Children’s Hospital Zurich, Zurich, Switzerland

With a population of 9 mio, Switzerland has about 80’000 newborns per year. Newborn screening is performed in one central lab situated in Zürich for all newborns in Switzerland and Liechtenstein. Mandatory private health insurance is covering the costs for the newborn screenings. The newborn screening program is organized nationally and is overseen by the Federal Office of Public Health (FOPH). Inclusion of a new disease has to be authorized by the FOPH following a formal application. Applications are usually initiated by a representative of a pediatric specialist organization together with the national newborn screening laboratory. Dried blood spot samples are collected in hospitals or at home by midwifes after informed consent of the parents. Samples are collected on PerkinElmer 226 filter paper 72–96 h after birth. Dried blood samples are sent by the national postal service and usually arrive at the lab within 1–2 days. Currently 11 diseases are screened: PKU, galactosemia, MCADD, GA-1, MSUD, biotinidase deficiency, cystic fibrosis, CH, CAH, SCID and SMA. Only abnormal screening results are reported to specialized pediatricians and samples are stored for 10 years following newborn screening for quality assurance purposes.

O03. **Country report—Finland**
Riikka Kurkijärvi
Finnish National Newborn Screening Centre (Saske), Tyks Laboratories Genomics, Turku University Hospital

This year, Finland celebrates the 10th anniversary of DBS NBS performed in the National Screening Centre (Saske) at Turku University Hospital. 430,000 newborns were screened for CAH and 20 metabolic diseases between 2015 and 2024, and 190,000 for SCID between 2019 and 2024. SCID-screening has been nationwide since 2022, except for Åland where it started in 2024. The overall incidence of these diseases was 1:4900. PKU remains exceptionally rare, with a total incidence of 1:40,000, including mild HPA (mostly) and BH4-deficiencies. The birth rate in Finland has decreased by 20% over the last decade. In 2023, it was the lowest in statistical history (43,383). The coverage of DBS screening in 2023–2024 (99.5%) was higher than in previous years (~99%). Screening results were also reported at earlier ages of children (median 6 days). Allowing sampling from 36 h of age may have had an impact. In 2023–2024, 35% of samples were taken at the age of 36–47 h. The nationwide cord blood screening for CH, introduced in 1980, is still ongoing in local laboratories. The incidence has been constant over the years, at 1:2783 between 1994 and 2017. In the last two years, a nationwide DBS screening for CH has been performed for comparison. As expected, the FPR of DBS screening is significantly lower (<0.1% vs. ~0.8%). The almost 100% coverage and very early initiation of treatment argue in favor of cord blood screening. The Ministry of Social Affairs and Health has recently asked the Council of Health Choices to evaluate SMA screening this year. The new EonisQ SCID-SMA method (Revvity) will replace the old Enlite method for SCID in April and hopefully will be introduced for SMA later.

O04. **Country report—Austria**
Maximilian Zeyda and Vassiliki Konstantopoulou
Austrian Newborn Screening, Department of Pediatrics and Adolescent Medicine, Comprehensive Center for Pediatrics, Medical University of Vienna, Vienna, Austria

Austria with its population of 9.1 million and around 80,000 births a year has a centralised newborn screening programme located at the Medical University of Vienna. The programme was initiated in the late 1960th and after introduction of mass spectrometry in 2002 and the latest expansion by screening for SMA and primary immunodeficiencies in 2021, 30 conditions are included in the screening, which are detected out of 7 primary punches taken from the DBS and 3 s tier tests. As one of few countries, we also officially screen for vitamin B12 deficiency. Participation is not mandatory but free of charge and the acceptance is very high (participation of >99.5%). The regular sampling age is 36–72 h and samples in most cases are sent via regular post. In case of urgently positive results, designated specialists of regional centers are contacted by the lab who contact and call in the families. Normal results are not reported. The focus of our work is not only continuous improvement of the screening and adaptions to the international development but also good collaboration with specialized centers all over Austria for optimal patient care.

### 3.2. Orals Selected from Sumitted Abstracts

O05. **Routinely Application of a High-Throughput Targeted Metabolomics 2nd Tier Test in Newborn Screening**
Michela Perrone Donnorso, Michela Cassanello, Luisella Alberti, Maria Cristina Schiaffino, Andrea Mascagni, Paola Vannini, Elvira Sondo, Cristina Cereda and Mohamad Maghnie
Buzzi Children’s Hospital, Functional Genomics and Rare diseases Newborn Screening Laboratory, Milan, Italy, IRCCS, Istituto Giannina Gaslini, Pediatric Clinic, Genova, Italy, IRCCS Giannina Gaslni, LABSIEM Pediadric Clinic—DINOGMI UNIGE, Genova, Italy, IRCCS Giannina Gaslni, LABSIEM Pediadric Clinic—DINOGMI UNIGE, Genova, Italy

Available 2ndtier tests (2TT) by liquid chromatography tandem mass spectrometry (LC-MS/MS) are commonly employed in Newborn Screening (NBS) on the presumptive positive dried blood samples (DBS) to reduce parental stress and economic burden due to unnecessary recall and expedite the diagnosis. Boosting 2TT specificity, speed and sensitivity, is challenged to improve performance metrics in NBS. We present highly-multiplexed 2TT LC-MS/MS non-derivatized assay for the analysis of 60 relevant metabolites among acylcarnitine, acylglycine, organic acids and amino acids derivatives for the simultaneous evaluation a broad panel of conditions integrated in most NBS panels. Analytes were extracted from one punched 3.2 mm DBS in a 25’ one-step procedure and analyzed within 11’ LC run in multiple reaction (MRM) acquisition mode and both positive/negative ionization. Quantitation was performed by stable isotope dilution technique. Analytical and clinical validation were assessed with certificated (CDC, ERNDIM) and home-made DBS material, healthy newborn and proficiency program. The analysis of 8500 samples out of 372,439 screens during a six years routinely 2TT application in two different labs, showed 100% sensitivity, a decrease of the recall rate from 2 to 0.5% and PPV improvement from 2.8% to 44%, considering together the disorders subjected to this assay. Reduction in turn-around time was also achieved. Our strategy is effective in streamlining and harmonizing 2TT workflow for both low and high throughputs in lab, promoting a better allocation of lab resources, and comparable results between labs. Flexibility by means of analyte addition/deletion and preliminary tests on other biological matrices allows method tailoring upon needs.

O06. **CLIR Status Update: Utilization, Regulation and Opportunities**
Patricia Hall, Dimitar Gavrilov, Devin Oglesbee, Matthew Schultz, Silvia Tortorelli and Dietrich Matern
Mayo Clinic, Laboratory Medicine and Pathology, Rochester, United States

Collaborative Laboratory Integrated Reports (CLIR) is a suite of post-analytical and data visualization tools designed to improve the performance of newborn screening (NBS), primarily for tandem mass spectrometry based assays, but with application to all biochemical assays included in NBS. CLIR is hosted by Mayo Clinic and access is available to any users willing to contribute data for the applications. As of 1 January 2025, there are almost 400 active users for NBS focused CLIR applications representing 77 screening programs. In 2024, over 1 million NBS profiles were analyzed using CLIR post-analytical tools. Exact counts for reference data vary by analyte, as not all programs have identical analyte profiles, however for most common amino acids and acylcarnitines there are between 3 and 5 million data points, and most profiles have 4 relevant covariates (sex, age at collection, birth weight and gestational age). CLIR’s database has 57,154 profiles utilized to build post-analytical tools, including cases and false positive profiles. This includes 3702 amino acid disorders, 3768 fatty acid oxidation disorders, 2351 organic acidemias, 614 lysosomal storage disorders and 88 peroxisomal disorders. Post-analytical tools are available for most disorders commonly screened for, although newly screened conditions can present challenges, and continued improvement. CLIR exists in an evolving regulatory environment, due to varying use cases and international usage. An application is being prepared for submission of CLIR’s NBS applications under the Software as a Medical Device to the United States Food and Drug Administration. It is expected that future international parallel regulatory applications may be needed as well.

O07. **A novel Neonatal Screening Modality: In-Depth Non-Targeted Proteome Analysis of Dried Blood Spots**
Daisuke Nakajima, Masaki Ishikawa, Ryo Konno, Yusuke Kawashima and Osamu Ohara
Kazusa DNA Research Institute, Department of Applied Genomics, Kisarazu, Japan

Here, we developed omics technologies to complement genome information obtained by genome sequencing. As an extension to this approach, we reported that an in-depth proteomic analysis of dried blood spots (DBS) can be used to detect patients suffering from inborn errors of immunity. However, the previously reported method was manual with several steps, hampering high throughput for implementation in real-world neonatal screening (NS). Therefore, we developed a simple protein extraction method for DBS that potentially meets the throughput required for NS and optimizes non-targeted proteomic analysis in combination with liquid chromatography-coupled mass spectrometry in data-independent acquisition mode (DIA-LC-MS/MS). The developed pipeline was termed non-targeted analysis of non-specific DBS-absorbed proteins (NANDA). We achieved the following: (1) automated extraction of proteins from 96 DBS punches (3.2-mm) in parallel; (2) identification of more than 5000 proteins using DIA LC-MS/MS; and (3) improvement of DIA-LC-MS/MS throughput to 40 samples/day with minimal compromise in protein coverage depth. These results imply that this pipeline can open new avenues for NS treatment using non-targeted quantitative proteome profiling, which is an unexplored modality in NS. We plan to conduct a feasibility study to evaluate the specificity of proteome-based NS in Japan. References1. J Proteome Res. 2020 Jul 2;19(7):2821–2827. doi: 10.1021/acs.jproteome.0c00271.2. J Clin Immunol. 2024 Oct 25;45(1):33. doi: 10.1007/s10875-024-01821-7.3. Methods Mol Biol. 2022;2420:39–52. doi: 10.1007/978-1-0716-1936-0_4.4. Proteomics. 2021 Dec;21(23–24):e2100019. doi: 10.1002/pmic.202100019.5. bioRxiv 2024 Dec 27, doi: 10.1101/2024.12.25.630353

O08. **Pilot Newborn Screening for Vitamin B12 Deficiency in Czechia: Benefit for Newborns and Their Mothers**
Truong An Nguyen, Richard Plavka, Jiri Zach, Viktor Kozich, Jan Janota, Katarina Ticha, Zbynek Stranak, Tomas Honzik, Samuel Stanovsky, Josef Bartl, Kristyna Nelicova, Petr Chrastina, Jakub Krijt, Jitka Sokolova and Klara Berkova
General University Hospital in Prague, and Charles University-First Faculty of Medicine, Department of Gynecology, Obstetrics, and Neonatology, Praha 2, Czech Republic, Thomayer University Hospital, Neonatal Unit, Praha 4, Czech Republic, General University Hospital in Prague, and Charles University-First Faculty of Medicine, Department of Pediatrics and Inherited Metabolic Disorders, Praha 2, Czech Republic, Motol University Hospital, Neonatal Unit, Praha 5, Czech Republic, Institute for the Care of Mother and Child, Department of Neonatology, Praha, Czech Republic

Background: Several European countries reported high birth prevalence of neonatal vitamin B12 (vB12) deficiency. We present data from a pilot project on neonatal vB12 deficiency in the Prague area. Methods: All dried blood spots (DBS) with elevated primary markers (C3 3.8 μmol/L or C3/C2 ratio 0.3 or methionine 7 μmol/L or C3/methionine ratio 0.5; determined by MS/MS) were subjected to LC-MS/MS second-tier testing for MMA (cut-off 2.5 μmol/L) and tHcy (cut-off 12 μmol/L). We recalled positive newborn cases together with their mothers and analysed total and active vB12, plasma tHcy and MMA, and maternal markers of chronic gastritis and selected autoantibodies. The newborn-mother pairs were offred a 3-day modified Cobasorb test using »10-times RDA of cyanocobalamin. Results: Acceptance of the pilot study was 85%. By the end of December 2024 we analysed 29,738 DBS with 4.1% ofsecond-tier tests. Of 8 newborns, 6 had neonatal vB12 deficiency, 1 was a false positive case, and 1 newborn was confirmed with biallelic mutations in the ACSF3 gene. The incidence of neonatal vB12 deficiency was 1:4956 (95% CI 1:2753-1:24,792). All newborns absorbed vB12, confirming the maternal origin of vB12 deficiency. Maternal vB12 deficiency was caused by a vegan diet (2×), unrecognized chronic (2×) or autoimmune gastritis (1×); one mother was lost for follow-up. Conclusion: Preliminary data demonstrate a high incidence of neonatal B12 deficiency in Prague area. Newborn screening for vB12 deficiency is beneficial for neonates and may also benefit their mothers. Supported by theMinistry of Health of the Czech Republic (NU22-07-00126 andDRO VFN64165) and Charles University (COOPERATIO Metabolic Disorders).

O09. **Evaluation of Neonatal Screening Programs for Tyrosinemia Type 1 Worldwide**
Allysa Kuypers ^1^, C. Austin Pickens ^2^, The ISNS representatives, Francjan J. van Spronsen ^1^, M. Rebecca Heiner-Fokkema ^3^, Kontantinos Petritis ^2^, Marelle J. Bouva ^4^, J. Gerard Loeber ^5^, Anita Boelen ^6^ and Eugenie Dekkers ^7^

^1^  University Medical Center Groningen, Beatrix Children’s Hospital, University of Groningen, Metabolic diseases, Groningen, Netherlands
^2^  National Center for Environmental Health, Centers for Disease Control and Prevention, Newborn Screening and Molecular Biology Branch, Division of Laboratory Sciences, Atlanta, United States
^3^  University Medical Center Groningen, University of Groningen, Laboratory of Metabolic Diseases, Department of Laboratory Medicine, Groningen, Netherlands
^4^  National Institute for Public Health and the Environment (RIVM), Centre for Health Protection, Bilthoven, Netherlands
^5^  International Society for Neonatal Screening (ISNS), International Society for Neonatal Screening (ISNS), Maarssen, Netherlands
^6^  Amsterdam Gastroenterology, Endocrinology &Metabolism, Amsterdam UMC, University of Amsterdam, Endocrine Laboratory, Department of Clinical Chemistry, Amsterdam, Netherlands
^7^  National Institute for Public Health and the Environment (RIVM), Centre for Population Research, Bilthoven, Netherlands

In The Netherlands, newborn screening (NBS) for tyrosinemia type 1 (TT1) uses dried blood spot (DBS) succinylacetone (SUAC) as a biomarker. However, high false-positive (FP) rates and a false-negative (FN) case show that the Dutch TT1 NBS protocol is suboptimal. In search of optimization options, we evaluated the protocols used by other NBS programs and their performance. We distributed an online survey to NBS program representatives worldwide (N = 41). Questions focused on the organization and performance of the programs and on changes since implementation. Thirty-three representatives completed the survey. TT1 incidence ranged from 1/13,636 to 1/750,000. Most NBS samples are taken between 36 and 72 h after birth. Most used biomarkers were DBS SUAC (78.9%), DBS Tyrosine (Tyr; 5.3%), or DBS Tyr with second tier SUAC (15.8%). The pooled median cut-off for SUAC was 1.50 µmol/L (range 0.3–7.0 µmol/L). The median cut-off from programs using laboratory-developed tests was significantly higher (2.63 µmol/L) than the medians from programs using commercial kits (range 1.0–1.7 µmol/L). The pooled median cut-off for Tyr was 216 µmol/L (range 120–600 µmol/L). Overall positive predictive values were 27.3% for SUAC, 1.2% for Tyr solely, and 90.1% for Tyr + SUAC. One FN result was reported for TT1 NBS using SUAC, while three FN results were reported for TT1 NBS using Tyr. The NBS programs for TT1 vary worldwide in terms of analytical methods, biochemical markers, and cut-off values. There is room for improvement through method standardization, cut-off adaptation, and integration of new biomarkers. Further enhancement is likely to be achieved by the application of post-analytical tools

O10. **Egypt’s Newborn Screening Journey: 25 Years of Progress and Innovation**
Mohammed Hassany
Ministry of Health and Population, Arab Republic of Egypt

Over the past 25 years, Egypt has made significant progress in newborn screening, transforming child health outcomes through the early detection of genetic and metabolic disorders. With a population exceeding 100 million in 2025, the high prevalence of consanguineous marriages, which leads to various genetic disorders, poses a critical public health challenge. The Egyptian Newborn Screening Program (ENSP) began in 1999, starting with screenings for congenital hypothyroidism (CH), a crucial step in preventing intellectual disabilities and developmental delays. The program expanded in 2015 to include phenylketonuria (PKU), addressing inherited metabolic disorders that can lead to severe neurological impairments if left untreated. A landmark advancement occurred in 2021 with the launch of the Presidential Initiative for Early Detection of 19 Genetic Diseases, which introduced expanded newborn screening for neonates in neonatal intensive care units (NICUs). This initiative employed advanced technologies to detect a wider range of genetic conditions, ensuring early diagnosis and timely intervention. To date, ENSP has screened approximately 50 million newborns, significantly reducing the burden of inherited metabolic disorders. Additionally, Recent genomic projects, including the Egyptian Reference Genome, further enhance precision medicine and support the identification of population-specific genetic variants. Future plans involve the complete digitalization of the screening program to facilitate continuous case follow-up and expand the screening panel to include common disorders in NICU-admitted infants. These initiatives highlight Egypt’s commitment to enhancing newborn screening and reinforcing public health strategies for future generations.

## 4. Poster Presentations

### 4.1. Biotidinidase Deficiency

P01. **Optimizing Newborn Screening (NBS) Services: Establishing Population-Specific Diagnostic Cutoffs for Biotinidase Deficiency (BTD)**
Hafsa Majid, Khairunnisa Mukhtiar, Muhammad Raza, Lena Jafri, Azeema Jamil, Nasir Ali Khan and Aysha Habib Khan
Aga Khan University, Dept. of Pathology and Lab Medicine, Karachi, Pakistan, Aga Khan University, Pediatrics and child health, Karachi, Pakistan

Introduction: To establish population-specific diagnostic cutoffs for dried blood spot (DBS) and serum biotinidase (BT) activity assays, and develop a clinical pathway for NBS, following ACMG guidelines. Methods: In this cross-sectional study, DBS and serum samples were collected of 120 healthy neonates (28 days) after parental consent. DBS and serum BT activities were measured by the fluorometric and colorimetric methods, respectively. Cutoffs for BT were established, and a diagnostic algorithm for NBS was developed. Results: DBS-BT Activity: Total 128 neonates were tested, with mean (SD) age 7.6 days (8.4), 57.8% (*n* = 74) male. A patient with BT activity lower than the manufacturer’s recommended cutoff (60 nmol/min/dL) was excluded. Mean DBS-BT activity of 127 neonates was 169.4 nmol/min/dL (74.7). As per ACMG, cutoff to report a positive screen sample based on 30% of the mean BT activity was 50.8 nmol/min/dL.Serum-BT Activity: Total 138 neonates were tested; of these 17 neonates with BT activity lower than the manufacturer’s recommended cutoff (3.5 U/L) were excluded from the study. Final analysis was done on 121 neonates, 61.9% (*n* = 75) were male; mean age and BT were 12.9 days (7.3) and 8.6 U/L (3.8), respectively. As per ACMG cutoff to report absolute (10% of mean serum BT activity) and partial deficiency (30% of mean serum BT activity) were 0.86 U/L and 2.58 U/L respectively. Diagnostic algorithm of BTD for NBS: All newborns will be tested for DBS-BT activity; those with BT levels 50.8 nmol/min/dL will be tested for serum BT activity. BTD will be diagnosed if serum BT levels are 2.58 U/L. Conclusion: The population-based cutoffs for BTD were significantly lower than the manufacturer-recommended cutoffs.

### 4.2. Congenital Adrenal Hyperplasia

P02. **21-deoxycortisone: A Novel Newborn Screening Marker of CAH due to 21-hydroxylase Deficiency**
Mark de Hora, Natasha Heather, Dianne Webster, Benjamin Albert and Paul Hofman
University of Auckland, Liggins Institute, Auckland, New Zealand, Auckland City Hospital, Newborn Screening, Auckland, New Zealand

Introduction. 21-deoxycortisol (21DF) is an accurate marker in newborn screening (NBS) for congenital adrenal hyperplasia (CAH). In CAH, accumulating 11-hydroxylated steroids are readily converted to 11-ketosteroids by 11β-hydroxysteroid dehydrogenase (11βHSD). We therefore postulated that 21-deoxycortisone (21DE), the 11βHSD metabolite of 21DF, may be a useful bloodspot marker of CAH.MethodsMeasurement of 21DE by LCMSMS was carried on 42 residual NBS specimens with a true positive (TP) result at screening, 11 with a false negative result (FN) and 441 specimens with a false positive result (FP). In the study, the test was considered positive if 21DE was detected. The sensitivity and specificity of 21DE as a marker for classical CAH was calculated and compared to 21DF screening and from a retrospective New Zealand study. ResultsThe method was linear to 1000 nmol/L and precision was 7.3–10.3%. The lower limit of quantification (LOQ) was 2 nmol/L and recovery was 99%. 21DE was ≥2 nmol/L in all 42 TP samples (range 20–601 nmol/L) and in 10 TN samples (0–54 nmol/L) and was not detected in the FP group. The sensitivity of 21DE to detect CAH was 98.1%. Specificity was 100%. A review of screening data from the same specimens from babies with CAH revealed that 21DF was ≥2 nmol/L in 41 TP specimens and in 6 FN samples (sensitivity 88.7%) and ≥2 nmol/L in 4 out of 1906 s tier tests performed between 2018–2021 (specificity 99.8%). Conclusions 21DE appears to be a sensitive marker for CAH in the newborn period. Incorporating 21DE into a second-tier test and adjustment of primary screening protocols could improve the sensitivity of NBS and eliminate FP screening results. Longer term studies on the performance of 21DE as a NBS marker are warranted.

P03. **Impact of Second-Tier Steroid Profiling on Newborn Screening for Congenital Adrenal Hyperplasia**
Patricia Hall, Dimitar Gavrilov, Devin Oglesbee, Matthew Schultz, Silvia Tortorelli and Dietrich Matern
Mayo Clinic, Laboratory Medicine and Pathology, Rochester, United States

Newborn screening (NBS) for Congenital Adrenal Hyperplasia (CAH) relies on measurement of 17-hydroxyprogesterone (17OHP). Screening for CAH can be confounded by falsely elevated 17OHP due to assay performance, prematurity and other physiological factors. To improve performance, second-tier testing (2TT) by liquid chromatography-tandem mass spectrometry (LC-MS/MS) can be used. The analytes vary by laboratory but includes at a minimum, 17OHP, androstenedione and cortisol, to directly evaluate the analytes surrounding the enzyme (21-hydroxylase) deficient in classic CAH.Our laboratory provides 2nd tier CAH testing for several NBS programs in the United States. From 1 January 2020–30 June 2024, we performed 2TT for CAH on 8791 specimens initially screened positive by the public health laboratory. The samples were collected at a median age of 42 h. Median birth weight and gestational age of the cohort were 2639 g and 35 weeks, respectively. 7779 (88.4%) were reported as negative based on 2TT results. 2TT results were positive in 1011 cases, of which 78 were confirmed by the referring site to have CAH. False positive results were confirmed in 798, 13 had expired before an outcome could be determined, 3 were lost to follow-up and for 119 cases outcome information was not provided. The minimum positive predictive value of the 2TT is only 7.7%. For confirmed cases, 21 were reported to have the salt-wasting form of CAH, 4 were simple virilizing and for 53 no information about subtype was provided. Different strategies to improve the performance of 2TT have been evaluated however there is considerable overlap between positive and negative steroid profiles which may be due to the early collection of NBS samples in the United States.

P04. **Newborn Screening for Congenital Adrenal Hyperplasia (CAH) in Slovenia: A Pilot Study**
Sara Colja, Daša Perko, Eva Kozjek, Domen Trampuž, Neža Založnik, Urh Grošelj, Ana Drole Torkar, Matej Mlinarič and Barbka Repič Lampret
University Medical Centre Ljubljana, University Children’s Hospital, Clinical Institute of Special Laboratory Diagnostics, Ljubljana, Slovenia, University Children’s Hospital, Department of Endocrinology, Diabetes and Metabolism, Ljubljana, Slovenia

Introduction: Congenital adrenal hyperplasia (CAH) refers to a group of genetic disorders affecting the adrenal glands. In CAH, enzyme deficiencies lead to abnormal hormone production, with the most severe form posing a life-threatening risk during the first weeks of life. Newborn screening (NBS) enables early detection, thereby allowing timely intervention and minimizing potential complications. This study aimed to determine the cut-off values for 17α-OH-progesterone (17α-OHP) and evaluate the proposed two-tier screening protocol for CAH in Slovenia. Methods: The pilot protocol follows a 2-tier strategy: 17α-OHP measurement in dried blood spots (DBS) as a 1st tier (GSP^®^ Neonatal 17α-OHP kit, Revvity) and analysis of steroid profiles using liquid chromatography tandem mass spectrometry (NeoMass CAH kit, Labsystems Diagnostics) as a 2nd tier, which is out of the scope of this abstract. Results: The pilot study began in 2024 and included 5069 DBS samples. Newborns were categorized into three gestational age (GA) groups: 4797 samples with GA 37 weeks, 179 samples with GA 35–36 weeks, and 93 samples with GA 35 weeks. Cut-off values for 17α-OHP were established for each group. For GA 37 weeks, the cut-off was set at the 99th percentile (13.9 nmol/L blood). For GA 35–36 weeks, the cut-off was set at the 95th percentile (21.3 nmol/L blood), and for GA 35 weeks, the 95th percentile was chosen as well (84.3 nmol/L blood). Using the established cut-off values, we identified 67 out of 5069 samples that require second-tier testing. Conclusions: This pilot study effectively established the cut-off values for the 1st tier of newborn screening (NBS), enabling us to accurately identify samples that require 2nd tier analysis.

P05. **digitalMLPA as a Potential Confirmatory Test for Congenital Adrenal Hyperplasia (CAH), Glucose-6-Phosphate Dehydrogenase (G6PD) Deficiency and Hemoglobinopathies in Newborn Screening**
Terence Diane Fabella, Lidewij Henneman, Jan Schouten, Eva Maria Cutiongco-de la Paz, Carmencita Padilla, Gerard Pals and Erik Sistermans
Amsterdam UMC locatie VU, Human Genetics, Amsterdam, Netherlands, MRC Holland, Research, Amsterdam, Netherlands, University of the Philippines Manila, IHG, National Institutes of Health, Manila, Philippines

Newborn screening (NBS) facilitates early diagnosis of newborn conditions wherein timely interventions result in improved long-term outcomes. The goal of NBS is to identify newborns’ risk for a certain treatable disease by using a test that is highly sensitive. Upon a positive result, a confirmatory test with high specificity should be used. This project aims to evaluate the use of SALSA^®^ digitalMLPA™ assays as confirmatory tests for the three most screened newborn conditions in the Philippines: Hemoglobinopathies, Glucose-6-Phosphate Dehydrogenase (G6PD) Deficiency, and Congenital Adrenal Hyperplasia (CAH).digitalMLPA is a multiplex PCR-based technique that uses Illumina platforms to simultaneously detect around 1000 target DNA sequences in one reaction. digitalMLPA can detect different types of genetic aberrations such as CNVs, methylation changes and selected SNVs. Besides purified DNA, this method allows the use of crude DNA extracts from a single 3.0 mm DBS punch. Preliminary results indicated a 100% detection rate of cell-line derived positive samples (Coriell). The digitalMLPA assays also detected 63.1% of Hemoglobinopathy, 66.7% of G6PD Deficiency and 15.0% of CAH-screened Filipino newborns (*n* = 161), as well as 31.5%, 26.7% and 5.0% of carriers of these disorders, respectively. The results also showed co-inheritance between Alpha- and Beta-Thalassemia 3.6% and G6PD Deficiency 2.7%. Future plans include analysis of more DBS samples as well as Sanger sequencing analysis to confirm digitalMLPA results of carriers and samples without mutations to determine the assays’ specificity, and positive and negative predictive values. These preliminary results show the potential of digitalMLPA to be used as a confirmatory test for NBS in the future.

P06. **Dried Blood Spot Steroids Profiling for Using Revvity QSight^®^ 225MD**
Tsun Au Yeung, Victoria Simonian, Joe Trometer, Hanna Polari and Juuso Huhtala
Revvity, Reproductive Health, Hopkinton, United States, Revvity, Reproductive Health, Turku, Finland

Congenital adrenal hyperplasia (CAH) is a group of disorders caused by inherited defects in steroid biosynthesis, most commonly, 21-hydroxylase deficiency (approximately 95% of cases) and 11- β hydroxylase (11β-H) deficiency (approximately 5% of cases). The overall incidence of CAH due to 21-hydroxylase (21-H) deficiency is approximately 1 in 15,000 live births. Impaired activity of this enzyme results in increased 17-hydroxyprogesterone (17-OHP), defective synthesis of cortisol (CORT) and mineralocorticoids, increased synthesis of androstenedione (ADIONE), and decreased central feedback (increased production of adrenocorticotropic hormone) resulting in adrenal hyperplasia, virilization, and salt-wasting crisis, which can be lethal. Hormone replacement therapy, when initiated early, results in a significant reduction in morbidity and mortality. Newborn screening for CAH is typically based on measurement of increased 17-OHP concentration in blood by immunoassays. However, these immunoassays are associated with a high number of false-positive results due to cross-reactivity of the antibodies with other steroids, particularly 17-hydroxypregnenolone. We report here a method utilizing a gradient UHPLC-MS/MS on the Revvity QSight^®^ 225MD- in dried blood spot (DBS). It allows simultaneous specific determination of 17-OHP, ADIONE, CORT, 21-deoxycortisol and 11-deoxycortisol. For Research Use Only. Not for use in diagnostic procedures

P07. **Optimizing Newborn Screening for Congenital Adrenal Hyperplasia: The Role of Second-Tier Testing in Preterm Newborns**
Javier Laguna, Ana Argudo-Ramírez, Abraham J. Paredes-Fuentes, José Manuel González de Aledo-Castillo, José Eduardo Flores-Jiménez, Carmen Martínez-Carreira, Tatiana Collado-Buzón, Anna Muñoz-Ramírez, Judit García-Villoria and Rosa Mª López-Galera
Hospital Clínic, Department of Biochemistry and Molecular Genetics, CDB, Barcelona, Spain

Introduction: Congenital adrenal hyperplasia (CAH) is a critical condition identified through newborn screening (NBS), and early diagnosis prevents severe complications. Premature newborns (PN) often require a second sample due to a higher risk of false positives (FP). This second sample generates family concerns and diagnostic delays. Second-tier tests (2TT) have proven beneficial in reducing FP in detection of many conditions, and it could also be beneficial for CAH.Objective: To assess the effectiveness of 2TT in PN, ensuring reliable CAH exclusion without a second sample. Material and Methods: In July 2024, Catalonia (Spain) began NBS for CAH, measuring 17-hydroxyprogesterone (17OHP) in dried blood spots (DBS) using fluoroimmunoassay (GSP^®^, Revvity), with gestational weeks (GW)-specific cut-offs (p97.5). Between July and September, for PN (34 GW), 17OHP, androstenedione (A), 21-deoxycortisol (21DOC), 11-deoxycortisol (11DOC), and cortisol (C) levels (Steroid Panel DBS, Revvity) were analyzed using ultra-performance liquid chromatography-tandem mass spectrometry (Xevo TQ-XS, Waters) on the first sample. A second DBS for 17OHP was requested after 15 days of life. Results: Analysis of 192 PN samples (23–33 GW; 122 males and 70 females) showed 17OHP values in the first DBS from 0.9 to 56.1 μg/L. p97.5 thresholds for 2TT were: 22.3 for 17OHP, 4.1 for A, 1.8 for 21DOC, 8.7 for 11DOC, and 76.8 μg/L for C. Of 189 s DBS received (three PN were exitus), 188 showed normal 17OHP values. Only one required reanalysis of 2TT in the second DBS, which were normal. To date, no false negatives have been reported. Conclusions: Our results show that the inclusion of 2TT in CAH newborn screening eliminates the need for a second sample in PN

P08. **Implementation of Congenital Adrenal Hyperplasia in the Newborn Screening Program in Catalonia with Second-Tier Test**
Tomas Honzik, Jiri Zach, Richard Plavka, Truong An Nguyen, Jan Janota, Katarina Ticha, Zbynek Stranak, Klara Berkova, Jitka Sokolova, Jakub Krijt, Petr Chrastina, Kristyna Nelicova, Josef Bartl, Samuel Stanovsky and Viktor Kozich
HOSPITAL CLINIC, Division of Inborn Errors of Metabolism-IBC, Department of Biochemistry and Molecular Genetics. FRCB-IDIBAPS. CIBERER, Barcelona, Spain, Hospital Sant Joan De Deu, Department of Pediatric Endocrinology. IRSJD, Esplugues De Llobregat, Spain, Hospital Vall D’Hebron, Department of Pediatric Endocrinology. CIBERER, Barcelona, Spain, Hospital Clinic, Division of Hormones, Oncobiology and Cytokines, Department of Biochemistry and Molecular Genetics. FRCB-IDIBAPS. CIBEREHD, Barcelona, Spain, Generalitat De Catalunya, Maternal and Child Health Service, Public Health Agency of Catalonia (APSCAT), Department of Health, Barcelona, Spain

Introduction: 17-hydroxyprogesterone (17OHP) is the marker for detecting congenital adrenal hyperplasia (CAH) in Newborn Screening Programs (NSP), but it has a high false positive rate. NSPs use strategies with cut-offs by gestational week (GW) and/or sex and request second samples from premature newborns (PN) to reduce this rate; some also use second-tier tests (2TT) to improve positive predictive value (PPV). Objective: To evaluate the implementation of CAH in our program and the impact of including 2TT on detection efficiency. Material and Methods: From July to December 2024, 17OHP dried blood samples (DBS) were analyzed by fluoroimmunoassay (GSP, Revvity). 2TT (17OHP, androstenedione [A], 21-deoxycortisol, 11-deoxycortisol and cortisol (Steroid panel DBS, Revvity) was applied in all samples ≥ cut-off by GW and in all PN (34 GW) by UPLC-MS/MS (Xevo TQ-XS, Waters). This strategy was validated in a cohort of PN (*n* = 192) by requesting a second DBS at 15 days. Positive cases were confirmed by analyzing 17OHP and A in plasma by LC-MS/MS and genetic study in the initial DBSResults: Cut-offs (p99) were calculated using 4474 DBS across different GW ranges (24–30;31–33;34–35;36;37;38;39 and ≥40). 27,747 DBS were analyzed: 398 PN and 27,376 (≥34 GW). 2TT (p97.5) were performed in 375 DBS: 178 (≥34 GW) and 197 PN. 29 s DBS were requested (18 for ≥34 GW and 11 for PN), with 17OHP normalizing in all DBS. Four positive cases were detected in the first DBS (1 male, 3 females), incidence1/6936. Diagnoses were confirmed in plasma between 4–7 days and by genetic studies Conclusion: The combined strategy of GW-based cut-offs, 2TT and mutation analysis in DBS improves the effectiveness (VPP = 100%), and eliminates the request for second samples in PN

P09. **The Effects of Neonatal Nutrition on 17-α-Hydroxyprogesterone Levels in Classic Congenital Adrenal Hyperplasia Screening: A Retrospective Study**
Maria Lucia Tommolini, Maria Concetta Cufaro, Silvia Valentinuzzi, Mirco Zucchelli, Ilaria Cicalini, Alberto Frisco, Gessica Di Carlo, Sara Verrocchio, Daniela Semeraro, Ines Bucci, Vincenzo De Laurenzi, Luca Federici, Damiana Pieragostino and Claudia Rossi
“G. d’Annunzio” University of Chieti-Pescara, Italy, Laboratory of Analytical Biochemistry, Proteomics and Endocrinology, Center for Advanced Studies and Technologies (C.A.S.T.), Chieti, Italy.

Background: The newborn screening (NBS) on dried blood spot (DBS) is mandatory for Italian law (n.167/2016). From 2022, Abruzzo region also provides screening tests for classical congenital adrenal hyperplasia (CAH), some immunodeficiencies, three lysosomal storage diseases and spinal muscular atrophy. In our laboratory, we screened 16,323 newborns for CAH. Following the positivity, 445 (2.73%) second tier tests (2-TT) were performed, 7 (0.04%) newborns required further attention and 4 (0.02%) positive cases were found. It is known that CAH screening is influenced by factors such as gestational age or weight, however we wondered whether different types of nutrition could also alter these results. Thus, we retrospectively evaluated the CAH screening performed, for samples collected between 0 and 120 h (colostrum production time) after birth, comparing those who consumed formula milk, colostrum or mixed nutrition. Methods: First level test for 17-α-hydroxyprogesterone (17-OHP) determination was executed by immunofluorimetric assays. 2-TTs were performed by ultra-performance liquid chromatography/tandem mass spectrometry (UPLC-MS/MS) evaluating a characteristic steroid profile. Samples were compared through univariate statistical analysis carrying out a *t*-test analysis. Results: In our experience, we analyzed 17-OHP levels of full-term infants who were fed with breast, formula milk or mixed nutrition in the first 5 days of life finding significant differences in term of higher 17-OHP concentration in formula-fed infants. Discussion: These results lay the foundation for developing new cut-offs. Different kind of nutrition, likely based on different type of milk composition, in the first 5 days of the infants could represent a determining factor in CAH screening.

P10. **Neonatal screening for CAH in Sweden and the Effects of Second Tier**
Karin Engström, Rolf H. Zetterström, Anna Wedell and Anna Nordenström
Karolinska University Hospital, Centre for Inherited Metabolic Diseases, Stockholm, Sweden, Karolinska University Hospital, Centre for Inherited Metabolic Diseases, Stockholm, Sweden.

Congenital adrenal hyperplasia due to 21-hydroxylase deficiency affects about 1/10,000–1/20,000 newborn babies worldwide, and 1/10,000 in Sweden. Newborn screening (NBS) for CAH, using 17-hydroxyprogesterone (17OHP) as a marker started in the 1970’s with the aim to prevent salt crisis and death in the neonatal period. The Swedish NBS program started in 1986. The positive predictive value (PPV) for CAH has been one of lowest among the NBS diseases, due to the presence of 17OHP and related steroids in stressed babies and poor specificity of the 17OHP immunoassays. A second-tier steroid hormone analysis measuring cortisol, 21-deoxycortisol, 11-deoxycortisol, 17-OHP and androstendione (A) by LC-MS/MS was implemented in 2021. Cut-off levels for term and preterm infants were determined in a retrospective study using true positive and false positive CAH NBS samples from our biobank. Results of the first three years using this two-tiered CAH-screening algorithm shows a prominent increase of the PPV. False positive screening cases were almost eliminated among term babies, and in total false positive screening cases decreased by 86%. The total PPV increased from below 7% to 35%. Moreover, 21-deoxycortisol is a useful marker that is highly specific to disease. The steroid ratio (17-OHP + A)/cortisol that has been widely used is not so good in finding CAH cases unless combined with 21-deoxycortisol. Our new two-tiered CAH screening algorithm works very well and has greatly improved our national CAH screening.

### 4.3. Cystic Fibrosis

P11. **Newborn Screening for Cystic Fibrosis in Slovenia: A Pilot Study Using a 3-Tier Strategy**
Daša Perko, Neža Založnik, Domen Trampuž, Sara Colja, Polona Lindič, Eva Kozjek, Tamara Obreza and Barbka Repič Lampret
University Medical Center Ljubljana, University Children’s Hospital, Institute for Special Laboratory Diagnostics, Ljubljana, Slovenia

Cystic fibrosis (CF) is a hereditary disorder affecting multiple organs. Early detection through newborn screening (NBS) enables timely intervention and reduces complications. This study aimed to determine cut-off values for immunoreactive trypsinogen (IRT) and evaluate the proposed 3-tier IRT/PAP/DNA protocol for CF screening in Slovenia. The NBS protocol involved measuring IRT as a 1st tier (GSP^®^ Neonatal IRT kit, Revvity), pancreatitis-associated protein (PAP) as a 2nd tier (MucoPAP-F, Dynabio), and DNA analysis for the 50 most common mutations in the CFTR gene as a 3rd tier test (Yourgene^®^ Cystic Fibrosis Base). The latter test was also validated on known Slovenian CF patients. The pilot began in 2024 and included 6076 samples. The cut-off and ultra-high cut-off values for IRT were set at the 98th and 99.9th percentiles, corresponding to 45 ng/mL and 76 ng/mL of blood. Of the samples tested, 115 exceeded the cut-off and underwent PAP analysis. Eight samples exceeded the ultra-high cut-off and were analysed for CFTR mutations. Out of 115 samples, 25 exceeded the threshold of 135 ng²/mL² for the IRT*PAP product and underwent mutational analysis. Among the 33 analysed samples, three had a heterozygous F508del mutation in theCFTRgene and were classified as NBS positives. Validation of the DNA panel using samples from 34 known Slovenian CF patients confirmed that all had at least one of the 50 CFTR mutations included in the test. Further confirmation tests, such as sweat tests and full CFTR gene analysis, are still required to finalise CF diagnoses. To fully validate the NBS protocol, testing on an additional 10,000 samples will further assess its accuracy in terms of positive predictive value (PPV) and identifying false negatives.

P12. **Cystic fibrosis Neonatal Screening in Hungary. A Single Center Evaluation of the First Two Years**
Andrea Xue, Judit Kincs and Ildikó Szatmári
Semmelweis University, Pediatric Center, Budapest, Hungary, Semmelweis University, Pediatric Center, Budapest, Hungary, Semmelweis University, Pediatric Center, Budapest, Hungary

Background: This presentation evaluates the strategy of the cystic fibrosis newborn screening (CFNBS) programme in Hungary based on the results of the first two years of screening. At the Hungarian NBS Centre located at Semmelweis University, Budapest, 92,063 neonates were screened for cystic fibrosis between February 2022 and December 2023. Methods: A combined CFNBS protocol with immunoreactive trypsinogen (IRT) and pancreatitis-associated protein (PAP) measurements were applied IRT/IRT×PAP/IRT with an IRT-dependent safety net (SN). Results: Out of 92,063 newborns, 303 were tested screen-positive. 14 cystic fibrosis (CF) and 3 cystic fibrosis-positive inconclusive diagnosis (CFSPID) cases were confirmed from the screen-positive cases, and 3 false-negative cases were diagnosed later. Based on the obtained results, a sensitivity of 82% and a positive predictive value (PPV) of 4.6% was calculated. Conclusion: Following the recognition of false-negative cases, the calculation method of the age-dependent cut-off was changed during the first year of screening. In purely biochemical CFNBS protocols, a small protocol change, even after a short period, can have a significant positive impact on the performance. CFNBS should be monitored continuously in order to fine-tune the screening strategy and define the best local practices.

P13. **Cystic Fibrosis Neonatal Screening in Luxembourg**
Anna-Maria Charatsi, Meriem Mastouri, Caroline Eisele, Patricia Borde, Marizela Kulisic, Abdelkader Heddar and Isabel De la Fuente Garcia
Centre Hospitalier de Luxembourg, National Centre of Paediatrics, Luxembourg, Luxembourg, National Centre of Paediatrics, Luxembourg, Luxembourg, Laboratoire National de Santé, Medical Biology, Luxembourg

Introduction: Cystic fibrosis (CF) neonatal screening was introduced on 1 January 2018 in Luxembourg. The implementation of the protocol was based on the characteristics of the Luxembourgish population which is multi-ethnic, with 50% of habitants being non-autochthones. Objectives: To describe the protocol for neonatal screening for CF in Luxembourg and evaluate its performance compared to recommendations for standards of care. Results: The Luxembourgish protocol for neonatal screening for CF relies on Immunoreactive Trypsinogen (IRT) on day 3 of life (D3) as the primary test. DNA analysis for CFTR gene variants is performed in case of high IRT (60 ng/mL), if the specific informed consent is signed by legal authorised representatives. The genetic testing is performed with the CF-EU2v1 panel (Yourgene^®^ Cystic Fibrosis Base) which includes 50 pathogenic variants and 4 polymorphisms of the CFTR gene. A safety net based on IRT on day 21 was introduced in June 2022. Patients screened positive are referred to the National Centre of Paediatrics where sweat test is performed. Genetic counselling is provided to families of all infants with pathologic genetic tests. Conclusion: CF NBS was introduced in Luxembourg in 2018. Further assessment is required in order to validate the diagnostic protocol.

P14. **Increasing Specificity, Sensitivity and PPV Using Next Generation CFTR Sequencing in Newborn Screening for Cystic Fibrosis**
Asbjorg Stray-Pedersen, Emma Lundman, Janne Maren Strand, Mari Eknes Ytre-Arne, Alexander Dominic Rowe, Anita Christine Senstad Wathne, Mette Engan and Egil Bakkeheim
Oslo University Hospital, Norwegian National Newborn Screening, Oslo, Norway, Oslo University Hospital, Norwegian National Newborn Screening, Oslo, Norway, Oslo University hospital, Norwegian Resource Centre for Cystic Fibrosis, Oslo, Norway, Haukeland university hospital, Department of Paediatrics and Adolescent Medicine, Bergen, Norway

Background: Next generation sequencing (NGS) was introduced in the newborn screening (NBS) for cystic fibrosis (CF) in Norway in 2015, as NGS provided more comprehensive CFTR variant coverage and increased second tier sample testing. The evaluation of this NGS workflow is presented here. Methods: NBS samples with immunoreactive trypsinogen (IRT) levels above the 96thpercentile (38–40 ng/mL) underwent targeted NGS using Illumina MiSeqDx CFTR 139variants assay and bioinformatics analysis of 13 additional variants. CF carriers and samples with IRT 120 ng/mL were selected for whole gene NGS with IonAmpliSeqCFTR on dried blood spot DNA. Only samples biallelic for likely pathogenic CFTR variants were considered screening positive and reported for diagnostic follow-up. Results: Out of 341,461 samples received during 2015–2021, 14,817 (4.3%) underwent variant-targeted NGS which identified 46 screening positive infants. Whole gene CFTR sequencing of 1046 samples added 22 screening positive. Among the 68 infants with positive screening, 50 versus 11 were diagnosed with CF versus CFSPID, a ratio of 1:0.22 in favour of CF with a sensitivity of 96.2% and a specificity 99%. The positive predictive value (PPV) increased from 0.43 to 0.74 with the NGS workflow. Two false negative screening results were identified, one with a novel missense variant and one with a copy number variant (CNV). Conclusions: NGS in CF screening improved sensitivity by analysing more samples for known pathogenic CFTR variants. Whole gene sequencing increased specificity and identified additional CFTR variants. Continuous variant re-evaluation further improved the PPV.

P15. **Ethnic Variation in the Incidence of Cystic Fibrosis in North Macedonia**
Violeta Anastasovska, Milica Pesevska, Angela Anastasovska, David Simonovski and Stojka Naceva Fustik
University Clinic for Pediatrics, Ss.Cyril and Methodius University in Skopje, Faculty of Medicine, Department for neonatal screening, Skopje, North Macedonia, Ss.Cyril and Methodius University in Skopje, Faculty of Medicine, Department for cystic fibrosis, Skopje, North Macedonia

Cystic fibrosis (CF) is a common monogenic disease with reported disparities in incidence corresponding to carrier rates among different ethnic groups. The newborn screening for CF in North Macedonia started as a pilot study in 2018, and become mandatory in April 2019. Immunoreactive trypsinogen (IRT) was measured from dry blood spots collected 48–72 h after birth on filter paper using the DELFIA time-resolved fluorescence immunoassay (referent value: 70 ng/mL). A second IRT test was preformed on 21st day after birth (referent value: 45 ng/mL). After a positive second IRT test, a sweat test was conducted. The final diagnosis was done through molecular testing of the CFTR gene. From 2018 to 2024 total of 112,265 newborns were screened, and a CF incidence of 1 in 3742 was estimated. During this period, 606 newborns were called for a second IRT test, resulting in a recall rate of 0.54%. A normal second IRT screening test was observed in 452 of these cases, and a sweat test was performed on 154 newborns. False positives were 124 cases (0.11%) while cystic fibrosis was detected in 30 newborns (0.027%) following positive sweat tests and molecular CFTR analysis. Nineteen of the CF cases were ethnic Albanians (63.3%), with an estimated incidence of 1 in 2358, while 11 were ethnic Macedonians (36.7%), with an incidence of 1 in 5973. The elevated CF incidence among Albanian newborns may be attributed to more frequent consanguineous marriages in this ethnic group over the centuries, leading to a higher percentage of CF carriers. CF is common in our country. We observed different ethnic distribution of the CF incidence in North Macedonia. Estimating CFTR mutation carrier rates may help explain the high CF incidence among the Albanian population.

### 4.4. Congenital Hypothyroidism

P16. **Regional Disparities in the Incidence of Congenital Hypothyroidism in North Macedonia**
Violeta Anastasovska, Milica Pesevska, Angela Anastasovska, David Simonovski, Nikolina Zdraveska, Mirjana Kocova, Elizabeta Taseva, Marija Jakovcevska and Marija Ciriviri
University Clinic for Pediatrics, Ss. Cyril and Methodius University in Skopje, Faculty of Medicine, Department for neonatal screening, Skopje, North Macedonia, Ss. Cyril and Methodius University in Skopje, Faculty of Medicine, Department for neonatal screening, Skopje, North Macedonia, University Clinic of Gynecology and Obstetrics, Ss. Cyril and Methodius University in Skopje, Faculty of Medicine, Department of infertility and assisted reproduction, Skopje, North Macedonia

Congenital hypothyroidism (CH) is one of the most prevalent preventable causes of mental retardation and intellectual disability in newborns. Genetic and environmental factors have been associated with an increasing incidence in various regions worldwide. A total of 422,218 newborns from all eight regions of the country were screened for CH, between 2002 and 2024 by measuring thyroid-stimulating hormone (TSH) levels from blood spots sampled 48–72 h after birth, using the DELFIA time-resolved fluorescence immunoassay. An overall CH incidence of 1/1696 was estimated in the country. The highest incidence was observed in the Vardar region (1/839), while the Eastern region had the lowest incidence (1/3425). In the other regions, the following CH incidence was detected: Northeastern 1/1589, Pelagonia 1/1200, Polog 1/1474, Skopje 1/1925, Southwestern 1/2297, and Southeastern 1/2087. Interestingly, in the Vardar region, 4.25% of screened newborns had a TSH concentration 5 mIU/L, as an indicator of regional iodine deficiency, compared to 1.57% in the Eastern region. Additionally, the presence of a mine deposit and two smelter plants in the Vardar region, out of a total of four smelter plants in the country, might be associated with the highest CH incidence in this region. The elevated CH incidence in some regions may be attributed to increased exposure to environmental toxic agents and/or deficient iodine intake. Further research into the potential environmental determinants of increased CH risk is warranted.

P17. **Newborn Screening for Congenital Hypothyroidism in Slovenia: Comparing Two Testing Systems and Establishing Cut-Off Values**
Domen Trampuž, Eva Kozjek, Sara Colja, Daša Perko, Neža Založnik, Ana Drole Torkar, Matej Mlinarič, Urh Grošelj and Barbka Repič Lampret
University Medical Centre Ljubljana, University Children’s Hospital, Clinical Institute of Special Laboratory Diagnostics, Ljubljana, Slovenia

Congenital hypothyroidism (CH) results from a shortage of thyroid hormones. Left untreated, it can lead to intellectual disability and slow growth, making timely detection crucial. Thyroid stimulating hormone (TSH) is measured in newborn screening (NBS) to detect potential CH. In Slovenia, NBS for CH is conducted by a different laboratory at the University Medical Centre Ljubljana (UMCL) than the rest of the NBS testing, which is done in the Newborn Screening Laboratory (NBS Laboratory) within the Clinical Institute for Special Laboratory Diagnostics (CISLD), UMCL. With the latest program extension, NBS testing from dried blood spot (DBS) samples will be consolidated and run by the NBS Laboratory. This pilot study aimed to compare results from both laboratories, to calculate and validate cut-off values (COV) for the new method. A total of 4846 fresh DBS samples were measured for TSH using GSP^®^ Neonatal TSH kit on the GSP^®^ analyzer (both Revvity, USA). These results were compared to those from the current screening laboratory, which uses the DELFIA Neonatal hTSH kit with Victor2TM D analyzer (both Revvity, USA). Bland-Altman analysis showed a bias of −4.35% (0.103 μU/mL) with an SD of −40.64%, indicating lower TSH measurements with GSP^®^ compared to Victor2TM D. The new COV was set at 6 µU/mL (99th percentile), as opposed to the current 8 µU/mL. To evaluate the COV, 161 samples including eight confirmed cases that, the previous year, exceeded the COV (≥8 µU/mL) on the Victor2TM D, were analyzed using the GSP^®^. Among these, 31 exceeded the COV (6 µU/mL) of which seven were confirmed CH cases. One sample would be classified as a false negative, likely due to TSH degradation. With this study we managed to successfully transfer the test to the NBS Laboratory.

P18. **Study of the Utility of Genetic Analyses in Congenital Hypothyroidism Neonatal TSH Screening**
Asbjørg Stray-Pedersen, Julie Hellem Aaby, Silje Hogner, Janne Strand, Cassandra Trier, William Tourniaire, Ingjerd Sæves, Trine Tangeraas, Mari Ytre-Arne, Siv M. Løvoll, Mona Christine Berge, Linda Karlsen Sørgjerd, Jens Jørgensen and Alexander Rowe
Oslo University Hospital, Norwegian National Newborn Screening, Oslo, Norway

Congenital hypothyroidism (CH) was included in the Norwegian newborn screening (NBS) program in 1979. Thyroid Stimulating Hormone (TSH) immunoassays (GSP, Revvity) are performed on all NBS dried blood spot samples. Genetic analyses have, since 2012, been used as a confirmatory test for most screening disorders, but not yet for CH. As part of improving the laboratory service, 42 genes associated with inherited CH were tested in samples with elevated TSH using Illumina DNA prep and bioinformatically filtered whole-genome sequencing. After nearly 260,000 analyzed samples (2018–2022), a total of 356 infants were reported with elevated TSH and were subjected to genome sequencing. Among the 350/356 sequenced infants, 22 individuals have a confirmed molecular cause of CH, and 57 are carriers of one pathogenic variant related to autosomal recessive CH,6 in compound heterozygozity with a variant of uncertain significance (VUS). In 80 individuals, one or more VUS was found, that might explain their increased TSH value. In some of these individuals, VUSs were found in several different genes associated with CH with a similar probability of pathogenicity. Sixteen individuals had Trisomy 21, with TSH values between 10 and 20 µU/mL. One individual had del22q11. In the remaining samples, no variants of interest were found. Among the individuals with a likely confirmed molecular cause of CH, the TSH levels were all above 20, with exceptions (DOUX2). The results of the project will be used for evaluation of whether NGS-based panel testing for determining the molecular causes of congenital hypothyroidism is useful in a NBS setting.

### 4.5. G6PD Deficiency

P19. **G6PD Deficiency Screening in Mexico, 12 Year Experience**
David Cervantes Barragan, Viridiana Arevalo Fragoso, Juana Navarrete and Rodrigo Reyna De Jesus
Pemex, Genetics, Mexico City, Mexico, Issste, Medical Director’s Office, Mexico City, Mexico, Ensayos Y Tamizajes De Mexico, Director’s Office, Mexico City, Mexico

Since the past decade, the public screening programs in Mexico have undergone a rapid expansion, introducing in panels disorders without previous extended description in Mexican population. The ISSSTE and PEMEX screening programs, introduced during 2019 and 2012 respectively, the routine screening for G6PD deficiency as mandatory test, defining as part of their guidelines the molecular testing to follow up to positive screening results. This study provides an overview about the outcomes of G6PD screening in about 230,500 newborns during 6 years in both Institutions, presenting the vast spectrum of variants detected in Mexican population, reaffirming as the most common enzyme deficiency in Mexico. Also presenting the challenges in operational controls to ensure the accuracy of tested samples, and the clinical follow up of confirmed cases to construct a robust screening program.

P20. **Prevalence of Glucose-6-Phosphate Dehydrogenase Deficiency in a Newborn Cohort from Romania**
Isabela Tarcomnicu, Tancuta Caju, Valentina Predoi, Catalina Sanda and David Patriche
Cytogenomic Medical Laboratory, Newborn Screening, Bucharest, Romania

Glucose-6-phosphate dehydrogenase (G6PD) deficiency is an X-linked genetic disorder resulting in a risk to develop neonatal hyperbilirubinemia, acute hemolysis or chronic hemolytic anemia, triggered by some medication, viral or bacterial infections, or ingestion of fava beans. It is the most common human enzyme defect (about 400 million people are affected worldwide). There is no universal screening for G6PD, although there are national programs in some countries (Philippines, Malaysia, Hong Kong, Singapore, Vietnam, Macau) and pilot programs in other countries. In 2022, our laboratory included G6PD in the screening panel offered to customers. Blood spot samples were collected on Whatman 903 filter paper. About 3300 newborns were tested for G6PD, upon informed consent of the parents. Analysis was performed using an enzymatic colorimetric kit from Zentech/LaCAR (Belgium). We used a threshold of 3.0 U/g Hb and 7.0 U/g Hb for severe and intermediate G6PD deficiency, based on literature and kit producer’s suggestions (we are in the process of adjusting the reference intervals to the local population). Of the total (3300) number of analyzed samples, 63 have shown a decreased enzyme activity, out of which 26 severe inhibition (0.79%) and 37 intermediate inhibition (1.12%). To our knowledge, this is the first data on G6PD obtained in Romania so far. With 1.9% severe and intermediate inhibition overall, the prevalence seems unexpectedly high for the studied cohort. Considering the increasing migration, the number of cases might rise in the future. Our results indicate that neonatal screening for G6PDD is useful for the early identification and optimal management of the patients who are at risk to develop hemolytic disease or hyperbilirubinemia in severe form.

### 4.6. General

P21. **Impact of Newborn Screening: Insights from La Vida en Una Gota; Documentary Film**
Pedro Lendinez, Maria Luz Couce Pico, Domingo González-Lamuño Leguina, Alvaro Hermida Ameijeiras, Raquel Yahyaoui Macías and Javier Blasco Alonso
Más Visibles Rare Deseases Association, Manager, JAEN, Spain, Complejo Hospitalario Universitario Santiago de Compostela, Neonatología, santiago de compostela, Spain, Hospital Universitario Marqués de Valdecilla, Pediatría, santander, Spain, Complejo Hospitalario Universitario de Santiago de Compostela, Medicina Interna, santiago de compostela, Spain, Hospital Regional de Málaga, Analisis Clínicos, Málaga, Spain, Hospital Regional de Málaga, Pediatría, Málaga, Spain

Newborn Screening (NBS) is a fundamental public health service, essential for the early detection of congenital disorders. The documentary La Vida en Una Gota examines NBS’s profound impact on families and healthcare systems, emphasizing the importance of early diagnosis and timely treatment. The film provides case studies of families whose children were diagnosed with metabolic disorders through NBS, exploring how screening processes vary across regions. It details advancements in screening technologies, policies, and the emotional and practical support extended to families. Interviews with healthcare professionals, policymakers, and parents offer a multi-perspective view of NBS’s effect on health outcomes and quality of life. The documentary reveals that early diagnosis through NBS greatly improves outcomes for children with metabolic disorders. Families expressed relief and gratitude for the prompt detection, which enabled timely interventions and effective management of their children’s conditions. Healthcare providers noted higher survival rates and enhanced quality of life among these children. However, the documentary also highlights disparities in NBS accessibility and implementation, with certain regions lacking the infrastructure and resources necessary to conduct comprehensive screenings. La Vida en Una Gota emphasizes the critical role of NBS in improving health outcomes for newborns with metabolic disorders and advocates for the global expansion and standardization of NBS programs. It calls for increased investment in infrastructure, specialized training for healthcare providers, and comprehensive family support systems to ensure that all newborns benefit equally from early detection and intervention.

P22. **Exploring Thailand’s Newborn Screening Journey: A Museum of Progress, Challenges, and Innovations**
Onanong Jaruan, Rotjanapan Pankanjanato, Pawinee Innark, Kannikar Punnapum, Suphattra Auttarawanit, Wiroj Puangtabtim and Hansa Thaisri
Ministry of Public Health, Medical Sciences, Nonthaburi, Thailand, Ministry of Public Health, Medical Sciences, Nonthaburi, Thailand, Ministry of Public Health, Medical Sciences, Nonthaburi, Thailand, Ministry of Public Health, Medical Sciences, Nonthaburi, Thailand, Ministry of Public Health, Medical Sciences, Nonthaburi, Thailand, Ministry of Public Health, Medical Sciences, Nonthaburi, Thailand, Ministry of Public Health, Medical Sciences, Nonthaburi, Thailand

Thailand’s newborn screening program, which began in 1996, initially focused on congenital hypothyroidism and phenylketonuria under the National Neonatal Screening Operation Centre (NSOC). The program was expanded in 2014 by Siriraj Hospital to include screening for 40 inborn errors of metabolism (IEM) disorders using Tandem Mass Spectrometry. Following advocacy from the Birth Defects Association of Thailand, the expanded newborn screening program (ENBS) was incorporated into the national health package in 2021. After a pilot study conducted by the NSOC, the ENBS was adopted in 13 health regions across the country in 2023. The ENBS program has made significant progress, achieving 96.77% coverage for IEM screening, with an incidence rate of IEM at 1:7433. With 10 ENBS screening units and 7 rare disease care centers across 13 health regions, Thailand has established a robust, nationwide network. A key innovation includes the development of a reliable Express Mail Service system for timely, trackable, and free sample transport, which has improved logistics and operational efficiency. Looking ahead, research is currently underway to introduce Next-Generation Sequencing as a second-tier screening for IEM and hearing loss in Thailand. Additionally, the NSOC has established a biobank in accordance with ISO: 20,387 standards to archive dried blood spot samples, serving as a national resource accessible to professionals and organizations for use in public health initiatives and research. Despite challenges, the ENBS program has made significant progress through collaborations, standardized practices, and continuous improvements. The program’s future focus is on expanding screening capabilities, integrating emerging technologies, and ensuring long-term sustainability.

P23. **Czech Republic: Report of Newborn Screening Results (period i/2010–xii2023)**
Felix Votava, Renata Gaillyová, Veronika Skalická, Milan Macek Jr, Andrea Holubová, Monika Hedelová, Hana Vinohradská, Eva Hlídková, David Friedecký, Tomáš Adam, Karolina Pešková, Viktor Kožich, Petr Chrastina and Iveta Valášková
3rd Faculty of Medicine, Charles University and University Hospital Kralovske Vinohrady, Department of Pediatrics, Prague, Czech Republic, 1st Faculty of Medicine, Charles University and General University Hospital, Department of Pediatrics and Inherited Metabolic Disorders, Prague, Czech Republic, Faculty of Medicine and Dentistry Palacky University and University Hospital, Laboratory for Inherited Metabolic Disorders, Olomouc, Czech Republic, Faculty of Medicine Masaryk University and University Hospital, Department of Clinical Biochemistry, Brno, Czech Republic, 2nd Faculty of Medicine, Charles University and Motol University Hospital, Department of Biology and Medical Genetics, Prague, Czech Republic, Faculty of Medicine Masaryk University and University Hospital, Department of Medical Genetics, Brno, Czech Republic

The newborn screening (NBS) in the Czech Rep. covers the entire newborn population and aims at detecting cong. hypothyroidism (CH), cong. adrenal hyperplasia (CAH), cystic fibrosis (CF), phenylketonuria/hyperphenylalaninemia (PKU/HPA), leucinosis (MSUD), glutaric aciduria type I (GA I), isovaleric aciduria (IVA), medium chain acyl-CoA, long chain 3-hydroxyacyl-CoA and very long chain acyl-CoA dehydrogenase deficiency (MCADD, LCHADD and VLCADD), carnitine palmitoyltransferase I and II deficiency (CPTD I and II) and carnitine-acylcarnitine translocase deficiency (CACTD). Since VI/2016 argininemia (ARG), citrulinemia (CIT), biotinidase deficiency (BTD) and homocystinurias (CBS and MTHFR deficiency) were added. Following a pilot study, the NBS program was expanded to SMA and SCID since I/2024. Methods: A total of 1,529,599 newborns (and a subset of 822,104 after expansion in VI/2016) were screened by use of fluoroimmunoassay for thyreotropin, 17-hydroxyprogesterone and immunoreactive trypsinogen (IRT). The CFTR gene (50 mutations) was analysed in 15,037 (0.98%) blood spots with the highest IRT levels. Amino acids and acylcarnitines were analysed by tandem mass spectrometry, analyte ratios and second tier test for total homocysteine were also used. Biotinidase activity was determined by fluorimetric assay. Results: We detected 1425 patients (cumulative detection rate 1:1022). The prevalence of particular disorders was as follows: CH 1:2870; PKU/HPA 1:4856; CF 1:6218 (CF-SPID not included with a prevalence of 1:18,210); BTD (including partial deficiency) 1:9905; CAH 1:12,335; MCADD 1:23,532; MSUD 1:94,258; LCHADD 1:101,973; GA I 1:152,960; IVA 1:191,200; VLCADD 1:254,933; CIT 1:411,052 and CBS 1:822,104.Supp: Cooperatio PEDI and CZ-DRO-VFN64165

P24. **Newborn Screening Technical assistance and Evaluation Program (NewSTEPs): A Model Resource Center for US Newborn Screening Programs**
Sikha Singh and Jelili Ojodu
Association of Public Health Laboratories, Newborn Screening and Genetics, Bethesda, United States

Measurable Objective: To describe how NewSTEPs, the nationally funded resource center in the United States, ensures that US newborn screening (NBS) programs can use data and implement quality improvement practices to evaluate, analyze, and potentially improve their performance. Methods: Reporting mechanisms for three tiers of data entered by NBS programs are made available to partners. Public state profiles share high level demographic, policy, program, and disorder related information. Results: NewSTEPs offers a voluntary data repository with uniform data elements. State and territorial NBS programs serve newborn populations ranging in size from 6100 births per year to over 500,000 (median 52,200). Regional laboratories serve 12 states, while all states have their own short term follow-up programs. Funding of NBS activities is secured from different sources, however most programs (*n* = 47/51) have an NBS fee (median $71, range $15–157). Storing residual dried blood spots ranges from 1 month to more than 27 years, with a median of 1 year. New disorders are added to state NBS panels via a variety of mechanisms (legislation *n* = 22, commissioner/board of health *n* = 18, other *n* = 2, unknown *n* = 9). Conclusion: NewSTEPs is the central source for data on NBS programs and supports quality improvement practices. The website contains reports, maps and infographics. The NewSTEPs repository and technical assistance resource center serves as a model for collecting, analyzing and sharing data in support quality improvement within a national public health surveillance program. This model can be extrapolated to global programs. NewSTEPs is funded through Cooperative Agreement # U22MC24078 HRSA/MCHB

P25. **Evaluation Criteria to Include Diseases in the French Newborn Screening Program**
Emmanuelle Ripoche, Nadia Naouir, Pascale Zagury and Andrea Lasserre
HAS, evaluation and access to innovation department, Paris, France

Background There is no international consensus on which diseases should be screened, revealing variability in the nature and number of screened diseases across the countries. French health authority (Haute Autorité de Santé-HAS) evaluates and proposes new diseases for inclusion in the national newborn screening (NBS) program. The aim of this study was to define standard criteria as a decision support tool for the NBS program. Method The criteria were defined based on a review of literature, and international practices. The feasibility and use of a multicriteria decision analysis method were successfully tested for the evaluation of 24 inborn errors of metabolism (IEM). Results The benchmark analysis compared the French criteria with those of other countries, revealing significant differences in disease inclusion criteria, despite all being based on the historical standards set by Wilson and Jungner. Six major criteria were selected in France: comprehensive understanding of the disease, symptom onset delay 7 days, disease severity, effective treatment, individual benefit from early intervention, screening test reliability. Non-decisional criteria, like incidence, organizational and economic impacts, are also analyzed. Conclusion The methodological guide on NBS by HAS provides an objective and reproductible framework to evaluate new diseases for inclusion in the national NBS program. By setting clear criteria and analyzing both decisional and non-decisional factors, the guide ensures a clear and transparent decision support tool. This multicriteria method was successfully applied for assessment and inclusion of 8 IEM, severe combined immunodeficiency (SCID) and spinal amyotrophy (SMA) in the French NBS program.

P26. **Newborn Screening in Slovenia—Establishing a Backup System for the GSP^®^ (Revvity) Analyzer**
Barbka Repič Lampret, Matej Mlinarič, Mojca Žerjav Tanšek, Ana Drole Torkar, Žiga Iztok Remec, Sara Colja, Eva Kozjek, Domen Trampuž, Daša Perko, Neža Založnik and Urh Grošelj
Unversity Medical Centre Ljubljana, University Children’s Hospital, Department of Endocrinology, Diabetes, and Metabolic Diseases, Ljubljana, Slovenia, Unyversity Medical Centre Ljubljana, University Children’s Hospital, Clinical Institute for Special Laboratory Diagnostics, Ljubljana, Slovenia, Unyversity Medical Centre Ljubljana, University Children’s Hospital

Early detection of diseases by newborn screening (NBS) is necessary for correct and timely clinical decisions. In 2025, Slovenia’s NBS will incorporate screening for congenital adrenal hyperplasia (CAH) and cystic fibrosis (CF), while congenital hypothyroidism (CH) testing will be relocated from another laboratory. Screening for all three diseases will be primarily performed on the GSP^®^ (Revvity) using proprietary reagent kits. Delays, including equipment faults, reagent shortages, or delivery challenges, might affect timely treatment; thus, implementing backup mechanisms is crucial to ensure fair care for all newborns. This study aimed to develop a back-up NBS system for all three conditions utilising Victor2TM D (Revvity) and proprietary reagent kits, as well as establish cut-off values (COV) for immunoreactive trypsinogen (IRT), thyroid stimulating hormone (TSH), and 17α-OH-progesterone (17α-OH-P). The study included 3000 samples for each method. The COV and ultra-high COV for the IRT were determined at the 99th and 99.9th percentiles, equating to 61.4 ng/mL and 120 ng/mL of blood, respectively. For 17α-OH-P, we established two gestational age (GA) categories: GA ≥ 37 and GA ≤ 36 weeks. The COV at the 99th percentile was 14.7 nmol/L and 55.5 nmol/L, respectively. For TSH, COV at the 99th percentile was 7 µU/mL. Three samples exceeded the ultra-high COV for IRT and were subsequently analysed for the 50 most prevalent CFTR mutations (Yourgene^®^ Cystic Fibrosis Base). One child was identified with a homozygous F508del mutation and classified as a true positive, marking the first infant in Slovenia diagnosed with CF through NBS. We successfully implemented a comprehensive backup screening system, which is crucial for the timely detection of diseases.

P27. **Appraising Advancements of Potential Candidate Diseases for Neonatal Blood Spot Screening Programmes: A Practical Framework**
Suzanne Onstwedder, Connor Hughes, Wendy Rodenburg, Marie-Louise Heijnen, Rinske Thoëne and Rose Maasse
Dutch National Institute for Public Health and Environment, Centre for Health Protection, Bilthoven, Netherlands, Dutch National Institute for Public Health and Environment, Centre for Health Protection, Bilthoven, Netherlands, Dutch National Institute for Public Health and Environment, Centre for Health Protection, Bilthoven, Netherlands, Dutch National Institute for Public Health and Environment, Centre for Population Screening, Bilthoven, Netherlands

Introduction: Fast developments in testing and treatments result in a continuous assessment of the extent to which diseases meet the established criteria for implementation in neonatal blood spot screening (NBS) programmes. We aimed to develop a practical framework with which to assess the advancements of potential candidate diseases for implementation in the context of the Dutch NBS programme. Methods: Based on published literature, a framework was developed. Five potential candidate diseases were assessed using literature and reports from key NBS programmes: Cerebrotendinous xanthomatosis, Duchenne muscular dystrophy, Metachromatic leukodystrophy, Pompe Disease, and Pyridoxine-dependent epilepsy. Results: Seven framework criteria were formulated: prevalence, disease course, screening test, clear path to the healthcare system and diagnosis, accessibility of effective treatment, implementation consequences for the NBS programme, and potential health gains. The criteria were evaluated for each disease to determine if future implementation is: impeded without any foreseeable solutions (x), partially or temporarily impeded with conceivable solutions (+/−), or not impeded (v). The availability of a screening test that demonstrably performs well in the proposed setting (e.g., relevant population, dried blood spots, applicable IVD regulations) is currently the key hurdle for implementation. Discussion: This approach provides a practical means to remain abreast of technological advances and implementation hurdles and stimulate timely research and identification of the strongest candidate diseases systematically. Although context dependent, applying this framework and sharing these insights internationally between NBS programmes may help guide endeavors in programme expansion.

P28. **N-Acetyl Tyrosine in Newborn Screening by Tandem Mass Spectrometry: A Single Center Experience**
Issam Khneisser and Dimitrios Platis
Institute of Child health, Newborn screening Laboratory, Athens, Greece, Saint Jospeh University, NBS-Laboratory CGGM-FM, Beirut, Lebanon

Given to babies, parenteral nutrition (PN) formula presents many challenges in Newborn screening (NBS) analysis and results interpretation. It elevates some amino acids (AAs) in NBS profile. During APHL-ISNS 2023, Austin Pickens presented a multiplexed novel biomarker indicative of PN formula in dried blood spot, the N-acetyl tyrosine (NALT). The ethical committee at Saint Joseph University of Beirut approved a pilot study on detecting the NALT in routine NBS,CEHDF 2613.NALT transition (224,136) using the Tyrosine IS for quantitation were added to the Neobase2 method on Qsight 225 MD. Among 8100 samples analyzed, 103 newborns were found with elevated NALT. NALT was not elevated in the detected MSUD case. A correlation between the elevation of NALT value and elevated branched amino acid (Leucine; valine) was observed but not found with other AAs like arginine, citrulline, etc. A difference in the mean of the two populations with elevated NALT versus normal NALT for leucine and valine values was significant (p 0.001). AAs mix with NALT via PN, increasing the value of branched amino acids. Adding NALT in routine analysis with no extra cost or extra workflow will rule out any human error, not mentioning TPN on of NBS card, inducing a reduction in the recall rate or in the 2nd tier analysis of Allo-Isoleucine. Furthermore, a new lower cutoff for valine and leucine can be taken into consideration with adding NALT analysis, as the latter will give additional information justifying any elevated findings. The only possible limitation of expanding this study is that NALT may not be present in AA mixtures given in NICUs.

P29. **Neonatal Screening Blood Sampling Age in the Neonatal Intensive Care Unit: Time Matters**
Andrii Lipatnikov, Tatiana Ivanova, Nataliia Samonenko, Mariia Haidei, Maryna Patsora, Alexandr Buryak, Volodymyr Olkhovych, Nataliia Mytsyk, Nataliia Olkhovich, Oksana Barvinska and Larisa Nikonova
National Children’s Hospital Okhmatdyt, Laboratory of Medical Genetics, kyiv, Ukraine, National Children’s Hospital Okhmatdyt, Center of Orphan Diseases and Gene Therapy, Kyiv, Ukraine, National Children’s Hospital Okhmatdyt, neonatal intensive care unit, Kyiv, Ukraine, National Children’s Hospital Okhmatdyt, Laboratory of Medical Genetics, kyiv, Ukraine

Background: Nationwide newborn screening (NS) program in Ukraine includes 21 disorders. The application of standard recommendations in blood sampling is significantly complicated in newborns in intensive care units (NICUs) due to the need for measures such as haemotransfusions, parenteral nutrition, drug infusions, etc., which significantly affects the results of NS. On the other hand, early diagnosis can significantly impact the effective correction of the newborn’s condition. Method: Retrospective analysis of the medical records Results: In 2 years, 375 newborns were treated in the NICU of the National Children’s Hospital Okhmatdyt. In the 79 newborns (21%), the blood was not sampled in standard terms (48–72 h) before transfer to the NICU. 26 newborns were preterm (24–31 weeks), and blood was sampled at the median age 32 days. In term newborns (*n* = 53), the blood was sampled at the median age: 20 days. The positive first result of NS was detected in 22 cases: 17OHP—6, TSH—4, IRT—4, C0—3, C4—3, Phe—1, Leu—1. The congenital hypothyroidism (CH) was confirmed in 3 babies. All other changes were interpreted as secondary after retest and 2-tier tests. The time of blood sampling in CH positive newborns was 17, 18 and 20 days. Correction of hypothyroidism significantly improved the children condition and accelerated their discharge from the NICU with improvement. Conclusion: The effectiveness of NS in the newborns in NICU can be improved by implementing double testing—the first blood draw before the start of treatment, even if it is earlier than 48 h, and the second after the child’s condition is stabilized. The first test allowed us to exclude conditions not dependent on nutrition or infusions and improve the child’s treatment.

P30. **Newborn Screening Implementation: Hurdles, Challenges and Way Forward**
Bhavna Dhingra, Shikha Malik, Ranu Tripathi and Sabavath Arun.
Pediatrics, AIIMS, Bhopal, India

Newborn Screening helps identify individuals with life-threatening disorders in presymptomatic period and early therapy helps reduce morbidity. India does not have a Universal newborn Screening Programme. Few public healthcare facilities offer newborn screening. Madhya Pradesh has a Neonatal mortality rate of 35 and Under 5 mortality rate of 55. At AIIMS Bhopal, we established a Newborn Screening Laboratory with support from State Health authorities to screen all inhouse newborns for the recommended core panel of metabolic disorders-

- Congenital Hypothyroidism
- Congenital adrenal hyperplasia
- G6PD Deficiency
- Biotinidase deficiency
- Galactosemia, and
- Hemoglobinopathies- considering high burden of sickle cell disease in Madhya Pradesh.

Procurement delays, sustained availability of consumables, and ensuring adequate load of samples were major hurdles in setting up a functional facility. Trainings were conducted onsite in other Govt. healthcare facilities of Bhopal and adjoining tribal districts for healthcare staff to ensure sample collection which could be transported to AIIMS for testing in a hub and spoke model. Irregular sample collection, poor sample quality, sample transport posed significant challenges probably due to lack of Newborn Screening being a Govt. programme. Online training programmes were conducted to provide ongoing support for collection of appropriate samples. Follow-up of Screen positives and getting a confirmed diagnosis were other major hurdles. Approximately 5000 newborns have been tested and the confirmed positive cases initiated on treatment and genetic counselling being offered to the affected families. G6PD deficiency is the most commonly diagnosed disorder. Increased awareness among community and health care professionals and strong commitment to implement universal NBS will go a long way in overcoming these challenges.

### 4.7. General-DNA Sequencing and Genetic Analysis

P31. **The Incorporation of Next-Generation Sequencing (NGS) in a Multi-Omics Screening Strategy into the NSW Newborn Screening Programme**
Enzo Ranieri, Tiffany Wotton, Won-Tae Kim, Shanti Balasubramaniam, Eric Law, Maija Siitonen and Zeq Ma
Sydney Children’s Hospital at Westmead, Newborn Screening; Biochemical Genetics, Sydney, Australia, Sydney Children’s Hospital at Westmead, Newborn Screening, Sydney, Australia, Sydney Children’s Hospital at Westmead, Metabolic Clinic, Sydney, Australia, REVVITY, Turku, Finland, REVVITY, Pittsburg, United States

The NSW Newborn Screening programme has implemented the use of NGS technologies into a Multi-Omic screening strategy to enhance the performance of the screening for inborn errors of metabolism (IEM). The use of DNA sequencing methods in newborn screening (NBS) has been utilised as 2nd tier testing since the early 1990 for the screening of cystic fibrosis (1). The use of DNA based testing has become mainstay in NBS with NSW introducing the screening of spinal muscular atrophy and severe combined immunodeficiencies using qPCR-based methods in August 2018. Projects world-wide looking into the use of NGS as a 1st tier test (BabySeq Project, USA, Genomics England, GenScan Australia). Whilst considerable progress has been made in technological advances in both sequencing and interpretive software it is limited by the lack of well-curated gene panels, labour intensive workflows as well as the challenges in data analysis and variant interpretation. The NSW NBS programme has overcome some of these challenges by adapting the use of an Element AVITI™ NGS System that amplifies and sequences DNA libraries in combination with a NEXTFlex NeoNGS kit from REVVITY that provides a robust genome coverage to identify variants in 396 genes. Data processing, from FASTQ to alignment and variant calling, was carried out using REVVITY Genomics Analyze software, and the variant interpretation was performed using REVVITY Genomics Interpret software. This was applied to the testing for several IEM that included MMACHC, MMADHC, LMBRD1, and ABCD4. Variants in the vLCAD gene (ACADVL), LCHAD (HADHA) and x-ALD (ABCD1). These IEM successfully identify the respective disease-causing variants in a high-throughput and cost-efficient manner. (1) Ranieri etal BMJ Vol 302 25 May 1991

P32. **AI-Powered, Vertically-Integrated IVD Certified Technology for Accurate, Fast Affordable Screening of Sex Development Disorders**
Lukasz Krych, Gjorgji Madjarov, M. Carmen Garrido Navas, Zoran Velkoski, Marija Chaushevska and Chris Kyriakidis
University of Copenhagen, Food Microbiology and Fermentation, Copenhagen, Denmark, Ss Cyril; Methodius University, Faculty of Computer Science and Engineering, Skopje, North Macedonia, University of Granada, Genetics Department, Granada, Spain, gMendel, Product Development, Copenhagen, Denmark, Ss Cyril; Methodius University, Faculty of Computer Science and Engineering, Skopje, North Macedonia, gMendel, Research; Development, Copenhagen, Denmark

The Problem Studied: Current diagnostic methods for genetic disorders rely on varying technologies based on the specific genetic alteration. Chromosomal abnormalities like Klinefelter syndrome (KS) or Turner syndrome (TS) are identified via karyotyping, FISH, or microarrays, which have limited resolution for small rearrangements and mosaicism. Point mutations, such as those causing cystic fibrosis (CF), are diagnosed using NGS after a sweat chloride test (SCT). Spinal muscular atrophy (SMA), caused by bi-allelic mutations inSMN1, is detected using RT-PCR forSMN1mutations andSMN2copy number. This fragmented approach is inefficient for newborn screening. A unified methodology could improve both efficiency and accuracy in newborn screening (NBS). Methodology: The study analyzed 356 blinded samples, including individuals with KS, TS, other disorders of sex development (DSD), and healthy donors. Using our SOP, we prepared sequencing libraries for ONT sequencing, multiplexing all samples into a single R10.4 flow cell for sequencing on a GridION device. AI-powered analysis on the CE-IVD-certified Phivea platform enabled comprehensive disease classification, without requiring skilled personnel (Bioinformaticians for analysis and Geneticists for interpretation). Results: The method achieved a specificity of 97.8% and sensitivity of 95.4%, reducing costs and accelerating diagnosis, demonstrating its viability for newborn screening. Conclusions: The Phivea platform effectively detects chromosomal aneuploidies, point mutations, and copy number alterations, making it an ideal tool for affordable and accurate mass screening in newborn care. Current experiments are extending its application to the 64 genetic disorders included in NBS across Europe.

### 4.8. General-Fatty Acid Oxidation Disorders

P33. **NBS for FAODs through the Lens of the Wilson and Junger Screening Criteria**
Ana Drole Torkar, Blanka Ulaga, Domen Trampuž, Tine Tesovnik, Daša Perko, Barbka Repič Lampret, Eva Kozjek, Marusa Debeljak, Vanja Cuk, Sara Colja, Žiga Iztok Remec, Tadej Battelino, Ana Klinc, Urh Grošelj, Mojca Žerjav Tanšek, Matej Mlinarič and Jernej Kovac
Children’s Hospital, UMC Ljubljana, Clinical Institute for Special Laboratory Diagnostics, Ljubljana, Slovenia, Children’s Hospital, UMC Ljubljana, Clinical Institute for Special Laboratory Diagnostics, Ljubljana, Slovenia, Children’s Hospital, UMC Ljubljana, Clinical Institute for Special Laboratory Diagnostics, Ljubljana, Slovenia, Children’s Hospital, UMC Ljubljana, Clinical Institute for Special Laboratory Diagnostics, Ljubljana, Slovenia, Children’s Hospital, UMC Ljubljana, Clinical Institute for Special Laboratory Diagnostics, Ljubljana, Slovenia, Children’s Hospital, UMC Ljubljana, Department of Endocrinology, Diabetes and Metabolic Diseases, Ljubljana, Slovenia

Since 2018, the Slovenian NBS program has included a group of FAODs using MS/MS and confirmatory WES testing, with VLCADD (8) and MCADD (8) being the most prevalent. The cumulative incidence of FAODs in the NBS era in Slovenia is 18.7 per 100,000 newborns screened. FAODs are an important group of IEMs, they are estimated to cause 5% of sudden and unexpected deaths in infants. The results of our systematic literature review confirm that a recognizable latent or early symptomatic stage does not exist in some cases, and NBS cannot always prevent the fatal outcome. Numerous MCADD, MTPD/LCHADD and CACTD patients presented with symptoms in the days before the NBS results were available. Adherence to the time frame for collecting, sending, and analyzing the samples is essential. Rapid action is needed for an initial positive NBS result, which should be communicated by a medical professional in charge of treating FAODs. Immediate metabolic advice is crucial for the survival of newborns detected by newborn screening. Rapid confirmatory genetic testing should be available for the FAODs. Clinicians caring for FAOD patients may consider partnership development across clinical and research networks to address the pitfalls of the NBS programs for FAODs, to produce per-country disorder-based overviews to compare screening performance, diagnostic confirmation, crisis interventions, and therapy follow-up for FAODs, and to advocate for advancements and work toward achieving equity in the NBS programs worldwide. As a next step, we would consider surveying centres involved in the NBS for FAOD to obtain further information on challenges and existing best practices.

### 4.9. General-Review

P34. **Newborn Screening for Genetic Metabolic Diseases by Tandem Mass Spectrometry in Selected Areas of Shanghai, China**
Tian Guo-li, Wang Yanmin, Ji Wei, Zhang Xiaofen, Zhou Zhuo and Guo Jing
Shanghai Children`s Hospital, Neonatal Screening Center, Shanghai, China

Genetic metabolic diseases are caused by gene mutations that disrupt metabolic processes, leading to severe health issues if untreated. Early detection via tandem mass spectrometry (MS/MS) enables timely intervention. Between 2010 December and 2024 December, newborn screening in Shanghai using MS/MS identified multiple disorders, providing vital data on disease prevalence and treatment outcomes. Methods: Blood spot collected from 325,719 newborns were analyzed. MS/MS quantified amino acids and acylcarnitines using a non-derivatized multi-amino acid, carnitine, and succinylacetone determination kit (PerkinElmer, USA), identifying abnormalities. Confirmatory tests included biochemical analysis, gene studies, and clinical evaluations. Maternal health factors were also assessed to ensure accurate diagnoses. Results: Among screened newborns, 104 cases of genetic metabolic diseases were confirmed (1 in 3132). The most common conditions were phenylalanine hydroxylase deficiency, hypermethioninemia, methylmalonic acidemia, and primary carnitine deficiency. Early treatment led to normal development in 92.3% of cases, while 4.8% faced severe outcomes, highlighting the importance of prompt diagnosis. Implications: This study demonstrates the critical role of MS/MS in newborn screening. Expanding screening programs and enhancing parental education are essential for better health outcomes. Future efforts should focus on integrating advanced technologies and addressing ethical and logistical challenges. Conclusion: Comprehensive newborn screening has proven effective in reducing the burden of genetic metabolic diseases in Shanghai. Continued innovation and policy support are crucial for sustaining these advancements.

P35. **Selective Newborn Screening for Inherited Metabolic Disorders in North Macedonia—Ten Years Experience**
Violeta Anastasovska, Mirjana Kocova, Angela Anastasovska, David Simonovski and Nikolina Zdraveska
University Clinic for Pediatrics, Ss. Cyril and Methodius University in Skopje, Faculty of Medicine, Department for neonatal screening, Skopje, North Macedonia

The introduction of tandem mass spectrometry in neonatal screening programs has increased the capacity of potentially detect inherited metabolic diseases (IEM). Early detection through newborn screening can potentially reduce morbidity, mortality and disabilities associated with the inherited metabolic disorders. Neonatal metabolic screening was performed by measuring of underivatized 12 amino acids and 13 acylcarnitines (Chromsystems Diagnostics, Germany) using LC-MS/MS method, in dried blood spot collected 48–72 h after births, during the period from 2014 to 2024. A total of 55.538 newborns (25.4% of the total neonatal population in the country during the study period) were screened through selective metabolic screening in North Macedonia. Newborns with prolonged stays in intensive care units and indication for IEM were also included. Twenty-six newborns with metabolic diseases were detected, with an estimated overall of 1/2136. Thirteen of them had MCAD (Medium-chain acyl-CoA dehydrogenasedeficiency), 10 had PKU (Phenylketonuria), and a single newborn had MSUD (Maple syrup urine disease), MET (Hypermethioninemia), and CPT I (Carnitine palmitoyltransferaseI)deficiency, respectively. Additionally, PKU was detected in five more children born in private birth centers which were screened at age between 10 months and 3.5 years, upon request of the Department of Neurology at the University Clinic for Pediatrics. Neonatal metabolic screening is an important diagnostic tool for identifying various types of IEM, providing substantial benefits to patients and their families. Early diagnosis is crucial not only for treatment but also for genetic counseling. Efforts to cover all newborns in North Macedonia are currently underway.

P36. **Pre-Analytical Neonatal Screening Indicators in Bulgaria 1993–2024**
Iskra Modeva, Violeta Kojuharova, Zoya Todorova, Tsvetoslav Petrov and Iva Stoeva
University Pediatric Hospital prof. Ivan Mitev, Neonatal Screening and Functional Endocrine Diagnostics, Sofia, Bulgaria

Quality indicators before the analytical screening part are of utmost importance for the precise diagnosis and timeliness of therapy start. The mass neonatal screening history in Bulgaria goes back to 1978 (Pilotstudy PKU, Bacterial Inhibition Assay), followed by primary congenital hypothyroidism in 1993, congenital adrenal hyperplasia in 2010. In 2025 3 more diseases—cystic fibrosis, spinal muscular atrophy and severe combined immunodeficiency are to be included. Since the beginning of the endocrine screening programs, many modifications and improvements have been implemented to: (a) increase the number of covered newborns (NB), (b) an earlier diagnosis, (c) change of treatment stategy. TSH was determined by DELFIA (cut off at initial screening 15 mU/l whole blood, addition of a second cut-off 8 mU/l whole blood for NB aged over 5 days, quality control DGKL) in 1,954,417 NB (till 31.12.2024), 17-OH progesterone (17-OHP cut off 99.5 centile, ISNS Norms) was determined in 883,886 NB. Data were registered by special software and analyzed by SPSS. The NB coverage increased from 58% in 1993 to over 95% in 2003 remaining permanently above this level, reaching 99–100%. Age structure at blood sampling also changed as follows: 2000: 1–2d—16.44%, 3–5d—78.4%, 5d—10.9% vs. 2024: 1–2 day—50.89%, 3–5 day—45.20%, 5 day—3.90%. Age at registration in the Screening Lab SBALDB improved: 2000: 11d—47% vs. 2024: 11d—3.1%, 9–11d—8.8%, 9d—87.99%. Stable improvement of all of the screening process indicators depends on constant feed back, data sharing and education at every level during the different stages of the NS program between all participants.

P37. **Expanded Neonatal Screening in Mexico, Experience of 12 Years: Spectrum Disorders And Molecular Findings**
Viridiana Arevalo Fragoso, David Cervantes Barragan, Rodrigo Reyna De Jesus and Juana Navarrete
Issste, Medical Director’S Office, Mexico City, Mexico, Pemex, Genetics, Mexico City, Mexico, Ensayos Y Tamizajes De Mexico, Director’S Office, Mexico City, Mexico

The introduction of aminoacids, acylcanitines and haemoglobins variants screening and next-generation sequencing technology as part of the routine protocols of two important public screening programs in Mexico, has revealed the complex genetic background of Mexican population, and has allowed identify the spectrum and frequency of IEM that during some decades had been just described as isolated cases. Our data from 230,500 screened newborns provide evidence about diversity of detected disorders, molecular findings and the clinical outcomes for confirmed cases under treatment. Our study demonstrates the benefits of expanded screening to provide guidelines useful for the construction of a robust expanded screening program for the entire population in Mexico

### 4.10. Hypermethioninemia

P38. **Screening and Genotype Characteristics of 21 Newborns with Benign Hypermethioninemia in China**
Fei Wang, Wei Ji, Yanmin Wang and Guoli Tian
Shanghai Children’s Hospital, School of Medicine, Shanghai Jiao Tong University, Department of Endocrinology, Shanghai, China, Peoples Republic, Shanghai Children’s Hospital, School of Medicine, Shanghai Jiao Tong University, Newborn Screening Center, Shanghai, China, Peoples Republic

Background: Hypermethioninemia (MATD) is a congenital amino acid metabolism disorder, which can be inherited in an autosomal recessive (AR) or dominant manner (AD). Methods: From 2022 to 2024, all patients with MATD were analyzed the levels of methionine (Met) in dried blood samples through newborn screening (NBS), the quantitative detection through secondary screening, and genetic testing through second-generation sequencing. Results: Among 21 NBS patients with high methionine levels, 8 carried MAT1A variants with AD inheritance, 5 carried R264H, and 2 carried A259V. One case carrying I252F is a novel variant. All AD patients have no clinical symptoms. Among the 13 AR genetic cases, a total of 11 reportedMAT1Avariants were detected. Only one AR genetic patient with P357L and M76T compound heterozygous variant had no clinical symptoms and her highest value is 87.97 μmol/L. The remaining 12 cases are AR genetic carriers. In AD patients, the levels of Met were significantly higher than of AR carriers, including the initial Met value of 132.59 (52.16–312.76) vs. 54.94 (30.78–77.69), the highest value of 220.11 (76.65–335.85) vs. 75.56 (50.36–115.05), and the secondary screening Met level of 160.75 (86.72–30.08) vs. 79.31 (58.51–148.78) μmol/L. Conclusions: Although children withMAT1Avariant AD inheritance have significantly higher Met levels than AR carriers, they still have a benign phenotype. Most cases of mild increase in Met levels during newborn screening are carriers. If the Met levels of AR genetic patients is mildly elevated and there are no clinical symptoms, it is still considered a benign disease. Keywords: MAT1A, Hypermethioninemia, Newborn screening

### 4.11. Information Technology in NBS

P39. **Enhancing Laboratory Efficiency: Implementation of Revvity Transcribe AI for Automated Data Entry in Newborn Screening**
Justin Anderle, Ephrem Chin, Andrew Millerschoen, Antti Mikkonen, Chris Murphy, Jacob Decker, Ken Sredzienski, Maria Ralph and Madhuri Hegde
Revvity, Newborn Screening, Waltham, MA, United States, Revvity, Omics, Pittsburgh, United States, Revvity, Digital Products, Turku, Finland, Revvity, Digital Products, Waltham, United States

Introduction: Manual data entry from handwritten documents into informatics systems is a time-consuming process, particularly in Newborn Screening facilities utilizing Dried Blood Spot cards. To address this challenge, we developed Revvity Transcribe AI, an innovative solution leveraging Optical Character Recognition and Machine Learning to convert handwritten text into a digital format. The primary objective of this product was to increase the processing speed of DBS cards and optimize laboratory staff time allocation. Methods: Revvity Transcribe AI was implemented at the Pittsburgh Revvity Omics laboratory. Two key metrics were evaluated: accuracy and processing speed improvement. Accuracy was determined by comparing Transcribe AI output with human-validated data. Processing speed improvement was assessed by measuring the time taken to populate data entry fields using Transcribe AI and subsequent human validation of the text. Results: Over a three-week period, Transcribe AI demonstrated a total field accuracy score of 73.3% over 1913 cards processed. During the first week, processing speed increased by 36%, from 36 cards per hour per person to 50 cards per hour. After two weeks, the processing speed further improved to 57 cards per hour per person, representing a 58% overall improvement in processing speed. Conclusions: Revvity Transcribe AI has proven to be a valuable tool in significantly enhancing the efficiency of laboratory test request form processing. By automating data entry, the system allows for more effective utilization of human resources, enabling staff to focus on other critical tasks. The implementation of Transcribe AI presents opportunities for laboratories to substantially increase their productivity.

P40. **Revvity’s MS/MS Workstation Software: Enabling Advanced MSMS Assay Workflows**
Hanna Polari, Thomas Hartmann, Suvituuli Poikonen, Axel Meierjohann and Tommi Elo
Revvity, Reproductive Health, Turku, Finland

Newborn screening (NBS) laboratories face increasing demands for improved timeliness, reduced number of false positives and expanding test menus creating more data. Revvity’s MS/MS Workstation Software addresses many of these challenges with a focus on supporting the new NeoLSD 7plex assay. The assay requires a unique two-punch, one-injection workflow, processing two punches from the same dried blood spot before combining for MS/MS measurement. The MS/MS Workstation helps to manage these workflows by tracking samples through multi-punch workflows, enabling the import of IS concentrations, QC targets and QC SD from a certificate with a barcode reader; combining data from separate preparation pathways; automated peak detection and algorithm integration, including quality flagging, saving review time; providing lists of samples requiring retesting and allowing for different cut-off values for retests; and providing customizable exporting and reporting tools. The interface allows sample tracking from punching to result reporting, integrating with LIMS for smooth data flow. Algorithms flag potential issues, allowing users to validate samples with minimal interaction enhancing sample turnaround time and quality control. The MS/MS Workstation’s adaptability supports broader trends in NBS, including LC-based assays, expanding panels, and customizable cutoff rules. It enables laboratories to implement cutting-edge screening methods, improving early detection of rare disorders and reducing unnecessary work in a newborn screening laboratory. Products may not be licensed in accordance with the laws in all countries, such as the US and Canada. Please check with your local representative for availability.

P41. **Design of a Custom Laboratory Information and Analysis System in C# in the Context of the Expanded Newborn Screening for Inborn Errors of Metabolism in Greece**
Dimitrios Platis, Panagiotis Girginoudis
Institute of Child Health, Department of Newborn Screening, Athens, Greece
Institute of Child Health, Department of Newborn Screening, Athens, Greece

Newborn screening (NBS) is an extremely important public health initiative aiming at identifying pre-symptomatic newborns that will develop significant disease if left undiagnosed and untreated. In Greece, the National Newborn Screening Program, initiated in 1974, is performed by the Institute of Child Health (ICH). Recently, the existing newborn screening program which up to that point screened for 4 conditions was expanded to include aminoacidopathies, fatty acid oxidation disorders, and organic acid metabolic disorders, as well as, cystic fibrosis. Although the National Newborn Screening Program is supported by a custom-made well-designed Laboratory Information System, additional analysis requires more flexible solutions that can be easily and economically optimized in order to handle the massive amount of data produced. For this reason, a project was initiated to design a flexible SQL-based software to handle the analysis of the data, as well as, provide a variety of functionalities such as Patient Database, Patient File, Appointment Calendar and Data Statistics and Data Input. The software was written in C# (Windows Forms) using Visual Studio 2022 Platform and contains over 60,000 lines of code.

### 4.12. Isovaleric Aciduria

P42. **False Positive Newborn Screening for Isovaleric Aciduria Revealed Three New Cases of 2-Methylbutyrylglycinuria**
Ana Škaričić, Ivana Križić, Korana Lipovac, Darija Šimić, Iva Bilandžija Kuš, Ksenija Fumić, Danijela Petković Ramadža, Tamara Žigman, Silvija Pušeljić and Ivo Barić
University Hospital Centre Zagreb, Department for Laboratory Diagnostics, Zagreb, Croatia, University Hospital Centre Zagreb, Department for Laboratory Diagnostics, Zagreb, Croatia, University Hospital Centre Zagreb; University of Zagreb, Department for Laboratory Diagnostics; The Faculty of Pharmacy and Biochemistry, Zagreb, Croatia, University Hospital Centre Zagreb; University of Zagreb, Department of Paediatrics; School of Medicine, Zagreb, Croatia, University Hospital Centre Osijek; University of Osijek, Department of Paediatrics; The Faculty of Medicine, Zagreb, Croatia, University Hospital Centre Zagreb; University of Zagreb, Department of Paediatrics; School of Medicine, Zagreb, Croatia

Background:2-methylbutyrylglycinuria (2-MBG; OMIM #610006) is an autosomal recessive disorder of isoleucine degradation caused by short/branched-chain acyl-CoA dehydrogenase deficiency (SBCAD). It is most often discovered through newborn screening (NBS) and is usually clinically asymptomatic. However, the clinical relevance of the deficiency remains unclear, because some patients have been reported to have developmental delay and other neurological symptoms. Case study and results: In three infants NBS showed slightly elevated C5 concentrations, 0.714, 0.833, and 0.81 µmol/L; N0.58 µmol/L. Although C5-acylcarnitine consists of a mixture of isomers (isovalerylcarnitine, 2-methylbutyrylcarnitine, and pivaloylcarnitine), isovaleric aciduria (IVA) was suspected, since C5 is the primary screening biomarker for IVA.Plasma acylcarnitine profiles showed moderately elevated C5-acylcarnitine (1.904, 1.14, and 1.592 µmol/L; N0.63 µmol/L), and organic acids revealed elevated 2-methylbutyryilglycine, the hallmark of 2-MBG, in all three patients. The diagnoses were confirmed by the ACADSB gene analysis, identifying heterozygous mutations, c.303+1G A and c.760G C in the first, and c.443C T and c.1159G A in the second patient. The third patient is still waiting for the genetic confirmation. Discussion: NBS for IVA can reveal SBCAD deficiency. Due to its questionable clinical relevance, it represents a challenge both for its position in the positive NBS diagnostic algorithm and further approach to the patient. All our patients are currently asymptomatic. Early recognition and long term follow-up of SBCAD deficiency may be beneficial, but also a burden for the patients and their families

P43. **Diagnosis of Isovaleric Acidemia: False Positive Due to Metamizole Metabolite**
Michela Perrone Donnorso, Giancarlo la Marca, Michela Cassanello, Elvira Sondo, Francesca Nastasia Perri, Maria Cristina Schiaffino and Mohamad Maghnie
IRCCS, Istituto Giannina Gaslini, LABSIEM—Pediatric Clinic, Genova, Italy, Meyer Children’s University Hospital, Newborn Screening, Clinical Chemistry and Pharmacology Lab, Florence, Italy, IRCCS, Istituto Giannina Gaslini, LABSIEM—Pediatric Clinic—DINOGMI UNIGE, Genova, Italy, IRCCS, Istituto Giannina Gaslini, Pediatric Clinic—DINOGMI UNIGE, Genova, Italy

Non-derivatized flow injection tandem mass spectrometry (FIA-MS/MS) is commonly used in newborn screening for the analysis of dried blood spot (DBS) samples. When false positive results are generated by indistinguishable isomeric and isobaric compounds, liquid chromatography (LC)-MS/MS second-tier test (2TT) is crucial for improving the positive predictive value. A C5-acylcarnitine-specific 2TT is routinely used to separate isomers following a C5 elevation in FIA-MS/MS analysis: isovalerylcarnitine and 2-methylbutyrilcarnitine are elevated in isovaleric acidemia and 2-methylbutyrylglycinuria,pilvaloylcarnitine related to pivalic acid-containing drugs/cosmetics and valerylcarnitine. We report the analytical strategy used to identify a new C5 isobaric compound in an 8-year-old child with a clinical picture of epileptic seizures, referred to the Gaslini Children Hospital with a significant hyperammonemia after a J-Peg replacement surgery. A drug therapy including intravenous metamizole was startedbefore requesting analysis ofDBS acylcarnitines, amino acid profile, urinary organic acids and plasmatic amino acids to exclude congenital metabolic diseases. The non-derivatized 2TTsurprisingly revealed the presence of an unknown peak at different retention time with respect to known isomers. It was identified as the inactive metamizole metabolite 4-acetylamidoantipirine (4-AAA) by means of LC-MS/MS and High-Resolution Mass Spectrometry (HRMS) experiments. Our biochemical finding demonstrated that metamizole intake can alter the measurement of C5-acylcarnitine generating false positive results, suggesting that the same interference could be potentially found during the rare off-label use of metamizole in infants and newborns.

### 4.13. Lysosomal Storage Disorders

P44. **Decade of Progress: The Evolution of Newborn Screening for Krabbe Disease in the United States (2013–2023)**
Astrid Pañeda Rodriguez, Maria Escolar and Kelly Bossola
Forge Biologics, Clinical Operations, Ohio, United States, Forge Biologics, Clinical Operations, Ohio, United States, Forge Biologics, Clinical Operations, Ohio, United States

Krabbe disease is a rare, inherited, neurodegenerative disorder with an estimated incidence of 1 in 100,000. It is a disease caused by mutations in the gene galactocerebrosidase (GALC), which is essential for normal catabolism of the important galactolipid component of myelin. Deficiency of GALC activity results in the accumulation of certain galactolipids, which damage myelinating glial cells, thereby causing inflammation, rapid demyelination, and progressive deterioration of the central nervous system and peripheral nervous system. Newborn screening typically uses a two-tiered testing process. Tier 1 involves screening for galactocerebrosidase enzyme activity, typically measured using fluorescence or mass spectrometry techniques, which quantify the GALC enzyme’s functional level. Tier 2 involves measuring psychosine levels in the infant’s blood sample, serving as a confirmatory marker, helping to differentiate onsets of the disease. Newborn screening in USA started in 2006 with New York after the advocacy efforts from Hunters Hope. In 2009 a petition was submitted to the Recommended Uniform Screening Panel (RUSP) to nominate Krabbe. The outcome was insufficient evidence for the Committee to make a recommendation to add the condition to the core panel, due to insufficient evidence of potential net benefit. A total of 7 new publications were able to address RUSP concerns. In August 2022 Krabbe was again nominated to the RUSP. However, on 1 March 2023, RUSP voted against approval for Krabbe NBS. A follow up from patient community to the new concerns presented was able to change the vote. On 22 March 2023, ACHDNC voted 10:3 to recommend adding infantile Krabbe to the RUSP to identify affected newborns and enable access to treatments.

P45. **Genetic Screening Analysis for Neonatal Lysosomal Storage Disorders: A Multicenter Study in China**
**Meng** Sun and Hui Zou
Jinan Maternity And Child Care Hospital Affiliated to Shandong First Medical University, Newborn Screening Center, Shandong, China, Peoples Republic

Objective: In this study, we analyzed the results of neonatal lysosomal storage diseases (LSDs) gene screening combined with enzyme detection, evaluated its application value, and understood the epidemic distribution of different types of LSDs. Methods: 29,601 newborn heel blood filter paper dry blood spots from February 2021 to December 2021 were collected from 8 provincial and municipal screening centers in China. High-throughput sequencing and target capture technology were used to detect the exon regions of 15 LSDs related genes. Sanger sequencing technology was used to verify the positive screening. At the same time, tandem mass spectrometry was used to determine the activity of related enzymes. Results: Among 29,601 newborns, 10 newborns were finally confirmed to be positive, the prevalence rate of 1/2960, and the positive predictive value was 66.67%. Among the confirmed newborns, 5 were Niemann Pick A/B disease, mainly distributed in southern cities. There were 3 Fabry disease and 2 Krabble disease. The diseases with higher carrying rate were Krabbe, Niemann Pick A/B, and Pompe. The total carrier rate of Hainan was the lowest, while that of Guangzhou and Jinan was more than three times that of Hainan Province. High frequency mutation sites were GALC gene C.1901T C, SMPD1 gene C.995C G, GAA gene C.2132_2133delCAinsG, and GLB1 gene c.2002A T. Conclusion: The overall incidence of LSDs in this project is high. Niemann Pick A/B has certain regional differences. Genetic screening and enzymatic tests are complementary to each other. It is suggested that Niemann Pick A/B, Fabry disease, Krabbe disease and Pompe disease should be preferentially included in the genetic screening of neonatal LSDs. Keywords: LSDs; newborn; gene screening; enzyme activity

P46. **Revvity NeoLSD™ MSMS Assay Feasibility Study with SCIEX Triple Quad™4500MD LC-MS/MS System**
Roberto Bozic
Revvity, Diagnostics, Milan, Italy

Lysosomal storage disorders (LSD) are inborn errors of metabolism caused by deficiencies in enzymes responsible for breaking down complex cellular components, resulting in accumulation of toxic species in the lysosomes. There is current expansion of newborn screening (NBS) programs to include lysosomal storage disorders because of the availability of treatments that produce an optimal clinical outcome when started early in life. All worldwide NBS for LSDs are performed as a first-tier test by measurement of lysosomal enzymatic activities in dried blood spots (DBS). The Revvity NeoLSD™ MSMS kit is based on a 6-plex-tandem mass spectrometry (MS/MS) lysosomal enzymatic activity assay for the following lysosomal storage disorders (LSD): Gaucher Disease, Niemann-Pick A/B Disease, Pompe Disease, Krabbe Disease, Fabry Disease, and MPS I Disease. The Revvity NeoLSD™ MSMS kit is an FDA cleared IVD kit launched originally 2018 and has been validated for multiple MSMS systems including QSight™ MD Screening Systems. The purpose of this study was to evaluate the performance of the Revvity NeoLSD assay with SCIEX 4500MD system. Performance evaluation were carried out in Clinical Biochemistry Laboratory of Città della Salute e della Scienza University Hospital, Turin. The MS/MS analysis was performed in positive ionization (ESI+) mode by flow injection analysis (FIA). The precision and accuracy were evaluated. The 6 lysosomal enzyme activities were measured on 6000 de-identified dried blood spot (DBS) punches, and screen positive samples were submitted for DNA sequencing to obtain genotype confirmation of disease risk. The number of screen positive samples, both in newborns and adults, were 23 overall (GAA, GLA, GALC, ASM and ABG) except for IDUA.

P47. **Withdrawn**

P48. Analytical Performance of a New Revvity NeoLSD™ 7plex LC-MSMS kit*
Heidi Appelblom, Tuija Hanses, Anu Kiviniemi, Laura Koskimäki, Tuomas Leppänen, Hanna Polari, Carita Rautanen and Jussi Suvanto
Revvity, Reproductive Health, Turku, Finland, Revvity, Reproductive Health, Turku, Finland

The performance of a new, multiplexed liquid chromatography-tandem mass spectrometry (LC-MSMS) kit* designed to screen for seven lysosomal storage disorders was evaluated. The kit measures the activities of the following enzymes which are deficient in their respective disorders: acid β-glucocerebrosidase (ABG, Gaucher disease), acid sphingomyelinase (ASM, Niemann-Pick A/B disease), β-galactocerebrosidase (GALC, Krabbe disease), α-L-iduronidase (IDUA, MPS I disease), α-galactosidase A (GLA, Fabry disease), acid α-glucosidase (GAA, Pompe disease), and iduronate 2-sulfatase (I2S, MPS II disease). The performance study was conducted on the QSight^®^ 225MD UHPLC Screening System. The evaluation included assessing precision, limit of blank, limit of detection, limit of quantitation, linearity, carry-over, and drift of the kit. Additionally, the performance and measured enzyme activities of the first six enzymes were compared to their respective results obtained using the existing Revvity NeoLSD™ MSMS kit (3093-0020). The results demonstrate that the new Revvity NeoLSD™ 7plex LC-MSMS kit provides sensitive, reliable and robust performance across clinically significant ranges for each measured enzyme. This makes it ideal for high-throughput newborn screening of all included lysosomal storage disorders. Furthermore, the kit shows comparable or improved results compared to the existing Revvity NeoLSD™ MSMS kit (3093-0020) in all studies. Products may not be licensed in accordance with the laws in all countries, such as the United States and Canada. Please check with your local representative for availability. *Product under development

P49. **Utilizing Automated Liquid Handlers for the Revvity NeoLSD™ MSMS Kit**
Martin Woergaard Kjær and Mette Nyegaard
Statens Serum Institut, Congenital Disorders, København S, Denmark, Statens Serum Institut, Congenital Disorders, København S, Denmark

The NeoLSD™ MSMS kit from Revvity is used to screen for 6 different lysosomal storage disorders (LSD). The kit is used to measure the activity of 6 different enzymes, by quantitively measuring the amount of product. The protocol is very labor-intensive, hence we sought out ways to reduce labor time. The protocol of the NeoLSD™ MSMS kit consists of an incubation part, extraction of products and analyzing the samples by mass spectrometry. After the incubation, the samples are quenched and afterwards the product is extracted by a liquid-liquid extraction. Several mixing steps are required, to ensure a robust quenching and extraction. By using the automated liquid handlers such as Biomek robots we are able to automate the whole process from quenching to reconstituted extract ready for analysis. By utilizing a 96-channel head we are able to analyze up to 800 samples a day, with easy capability of upscaling. This has more than doubled the number of samples we can analyze daily and furthermore halved the labor time. To conclude we have shown that utilizing automated liquid handlers for high-throughput neonatal screenings can significantly increase the number of samples that can be analyzed simultaneously reduce labor time.

### 4.14. Methods in NBS-2nd Tier Methods

P50. **LC-MS/MS Measurement of Methylmalonic Acid, 3-Hydroxypropionic acid, 2-Methylcitric acid, and Homocysteine from Dried Blood Spots using the QSightTM 225 MD UHPLC Screening System**
Hanna Polari, Juuso Huhtala, Joe Trometer, Victoria Simonian and Tsun Au Yeung
Revvity, Reproductive Health, Turku, Finland, Revvity, Reproductive Health, Hopkinton, United States

Elevated propionyl carnitine (C3) and its ratio with acetyl carnitine (C3/C2) are used as general markers to indicate possible propionate metabolism related disorders by newborn screening laboratory. Flow injection tandem mass spectrometry analysis (FIA-MS/MS) assays can be prone to false-positives due to isobaric interferences. Such assays can also lack the sensitivity to detect more disease specific organic acid biomarkers. The most common inborn metabolism diseases attributed by the elevated C3 and/or C3/C2 are methylmalonic and propionic acidemia with incidence of around 1:50,000 and 1:150,000 respectively. The most common subtype of methylmalonic acidemia is combined methylmalonic acidemia with homocystinuria where incidence is about 1 in 200,000 newborns worldwide. Herein we describe a liquid chromatography tandem mass spectrometry (LC-MS/MS) method to measure methylmalonic acid (MMA), total homocysteine (tHcy), and 2-methylcitric acid (MCA), which are biomarkers of interest for methylmalonic acidemia, along with 3-hydroxypropionic acid (3-OHPA), a biomarker of interest for propionic acidemia. For research use only. Not for use in diagnostic procedures.

### 4.15. Methods in NBS-DNA Sequencing and Genetic Analysis

P51. **Newborn Screening by DNA-First: Systematic Evaluation of the Eligibility of Inherited Metabolic Disorders Based on Treatability**
Abigail Veldman
University Medical Centre Groningen, University of Groningen,, Pediatrics, division of Metabolic Diseases, Groningen, Netherlands

The biomarker-based Dutch Newborn Screening (NBS) panel (as of 2024) comprises 19 inherited metabolic disorders (IMDs). With the use of next-generation sequencing (NGS) as a first-tier screen, NBS could expand to include IMDs that lack a reliable biochemical footprint in dried blood spots, while also reducing secondary findings. To be eligible for inclusion in NBS, an IMD needs to fulfill the Wilson and Jungner criteria, with treatability being one of the most important criteria. In this study, we aimed to identify IMDs eligible for DNA-first NBS when considering only treatability in the context of NBS as a prerequisite. First, three independent reviewers performed a systematic literature review of the 1459 genotypic IMDs and their causative gene(s), as described in the International Classification of Inherited Metabolic Disorders (dated 1 February 2021), applying 16 criteria to exclude non-treatable disorders. Eligible disorders were then discussed in three online meetings with a project group of clinical laboratory geneticists, medical laboratory specialists specialized in IMD, and pediatricians with expertise in IMDs. Based on treatability, we identified 100 genes, causing 95 IMDs, as eligible for NBS, including 42 causal genes for the IMDs in the current biomarker-based NBS. The other 58 genes are primarily associated with treatable defects in amino acid metabolism and fatty acid oxidation. Other IMDs were excluded, most often because of insufficient literature. As the evaluation of treatability was not straightforward, we recommend the development of standardized treatability scores for the inclusion of IMDs in NBS.

P52. **digitalMLPA: A DNA-Based Technique to Fill the Gap between Single Target Assays and NGS for Newborn Screening**
Joery den Hoed, Terence Fabella and Jan Schouten
MRC Holland, Research, Amsterdam, Netherlands, Amsterdam UMC, locatie Vrije Universiteit, Human Genetics, Amsterdam, Netherlands, MRC Holland, Cytogenetics, Amsterdam, Netherlands

Newborn screening (NBS) is a public health program aiming to detect early-onset conditions that benefit from timely intervention. DNA-based techniques provide opportunities for confirmatory testing and expansion of the NBS panel. However, currently used methods such as Sanger sequencing and qPCR still require a condition-specific workflow. Next generation sequencing (NGS) has the potential to test for a large number of conditions simultaneously. However, there are concerns about data interpretation, privacy and costs. We are developing a low-cost assay that could fill the gap between single-region DNA-based assays and NGS, based on SALSA^®^ digitalMLPA™. This probe-based method uses Illumina platforms for amplicon detection and quantification, allowing the use of around 1000 probes in a single reaction. Our digitalMLPA assay can detect copy number variants (CNVs), selected single nucleotide variants (SNVs), as well as DNA methylation and is compatible with both purified genomic DNA and crude extracts from a single dry blood spot (DBS) punch, allowing high-throughput processing. This digitalMLPA assay contains probes to detect CNVs and SNVs in genes associated with more than 30 genetic conditions, including the complex genes CYP21A2, SMN1 and HBA1/HBA2. Balanced F8 inversions can also be detected. Moreover, the assay can detect DNA methylation status of genomic regions associated with Fragile X, Prader-Willi, Angelman, Beckwith-Wiedemann and Silver-Russell syndromes. Preliminary data on cell line- and DBS-derived DNA demonstrates that our digitalMLPA assay can confidently call CNVs in target regions, and is able to detect the presence or absence of SNVs and changes in DNA methylation. These results highlight the potential of digitalMLPA as valuable DNA-based tool in NBS.

P53. **digitalMLPA: A New, Multiplexable, DNA-Based Method for Screening of Newborn Conditions**
Terence Diane Fabella, Erik Sistermans, Lidewij Henneman, Gerard Pals, Joery den Hoed, Carmencita Padilla, Eva Maria Cutiongco-de la Paz and Jan Schouten
MRC Holland, Research, Amsterdam, Netherlands, Amsterdam UMC locatie VU, Human Genetics, Amsterdam, Netherlands, Amsterdam UMC locatie VU, Human Genetics, Amsterdam, Netherlands, Amsterdam UMC locatie VU, Human Genetics, Amsterdam, Netherlands, MRC Holland, Cytogenetics/Research, Amsterdam, Netherlands, University of the Philippines Manila, IHG, National Institutes of Health, Manila, Philippines

DNA analysis tools are essential to detect genetic alterations for disorders eligible for newborn screening (NBS) for which no biochemical method exists. Melt curve tests and qPCR for SMA and SCID are already applied in many countries, but these methods cannot be easily multiplexed. The implementation of targeted Next Generation Sequencing or whole genome sequencing for NBS is hampered by costs, ethical issues and technical limitations for analysis of complex regions and methylation status. This study investigates SALSA^®^ digitalMLPA™ as a first-tier screening method for newborn conditions and as a complementary test for biochemical methods to increase sensitivity and specificity. digitalMLPA is a multiplex PCR-based technique that detects around 1000 target DNA sequences in a single reaction using Illumina platforms. We designed two digitalMLPA assays that require only a crude DNA extract from a single 3.0 mm punch of a DBS card. These assays can test up to 384 samples in one run. Initial tests on cell line-derived DNA showed that digitalMLPA can successfully detect CNVs, methylation changes, and selected SNVs. Testing of 74 randomly selected DBS cards from Filipino newborns detected one patient with a GJB2 homozygous p.V37I variant. The majority of the carriers had GJB2 variants (28%) and HBA1 and HBA2 deletions (11%) associated with hearing loss and Alpha-thalassemia, respectively. Carriers of G6PD Deficiency, Duchenne Muscular Dystrophy and Batten disease were also detected (7%). As digitalMLPA focuses on selected CNVs, SNVs, small INDELs and methylation changes, this simplifies result interpretation and adaption to national ethical and legal demands, highlighting the potential of this technique for NBS.

P54. **A Streamlined Newborn Sequencing Research Workflow Using a Curated Gene Panel**
Riina Kaukonen, Marika Sjöqvist, Minna Niemelä, Ephrem Chin, Zeq Ma, Lisheng Wang, Kristina Fura, Amber Woodman, Leni Kauko, Supan Dhillon, Cristina da Silva, Maija Siitonen and Madhuri Hedge
Revvity, R&D, Turku, Finland, Revvity Omics, Genomics, Texas, United States, Revvity Omics, Genomics, Georgia, United States, Revvity Omics, Genomics, Pittsburgh, United States, Revvity, Business, Turku, Finland, Revvity Omics, Genomics, California, United States, Revvity Omics, Genomics, Duluth, United States

Next-generation sequencing (NGS) enables high-throughput and cost-effective detection of disease-causing variants. Recently, NGS has been used as a 2nd tier testing strategy in newborn screening (NBS) and growing evidence suggests a broader potential for NGS in NBS. Broader use of NGS has been limited by lack of well-curated gene panels, labour intensive workflows and challenges in data analysis and variant interpretation. We have developed a Neo NGS research workflow, adapting a proven chemistry pipeline with 40,000 samples, covering reagents, instruments and analysis tools. Our workflow covers isolation of high-quality DNA from dried blood spot cards, whole blood and saliva, followed by NGS library preparation, including quality control (QC) and data/variant analysis software. Sequencing libraries were prepared using Revvity’s NEXTFLEXTM Neo NGS RUO Panel 1 kit. DNA was enzymatically fragmented, followed by addition of indexed adapters for sample multiplexing. Libraries were enriched by hybridization using probes targeting 396 pre-curated genes associated with early-onset conditions. The resulting library is suitable for sequencing on various platforms including those of Element Biosciences and Illumina. Data processing from FASTQ to alignment as well as variant calling, was carried out using Revvity Genomics Analyze software. Variant interpretation was performed using Revvity Genomics Interpret software. Automation and the intuitive lab-designed software minimizes errors and facilitates easy sample volume scaling. This automated workflow with an expert-designed panel streamlines testing and analysis, addressing key barriers to NGS adoption in newborn sequencing research. For research use only. Not for use in diagnostic procedures.

P55. **Next-Generation Sequencing and Other Second Tier Tests in Newborn Screening for (X-Linked) Agammaglobulinemia**
Maartje Blom, Annelotte J. Duintjer, Ingrid Pico-Knijnenburg, Sandra Ilmholz, Sahila Balkassmi, Hermine van Duyvenvoorde and Mirjam van der Burg
Leiden University Medical Center (LUMC), Pediatrics, laboratory for Pediatric Immunology, Leiden, Netherlands, RIVM, Centre for Health Protection, Bilthoven, The Netherlands, Leiden University Medical Center (LUMC), Clinical Genetics, Leiden, Netherlands

Purpose Patients with X-linked agammaglobulinemia (XLA) suffer from severe, recurrent infections potentially leading to life-threatening complications. Early diagnosis and timely treatment can prevent infections and secondary complications, emphasizing a role for newborn screening (NBS). NBS for XLA is based on quantification of kappa-deleting recombination excision circles (KRECs). KREC-based screening could result in a large number of false-positive referrals associated with high impact for parents and health care systems, indicating the need for a second-tier test. Methods. KRECs were measured in NBS cards (N = 110,491) with a multiplex TREC/KREC qPCR assay. As second tier test options, an alternative qPCR multiplex assay, epigenetic immune cell counting for relative B-cell quantification and targeted next-generation sequencing with B-cell deficiency gene panel including 73 genes were performed on NBS cards of newborns with low KRECs. Results. In total, 136/110,491 newborns had KRECs below cut-off. With the alternative qPCR multiplex assay, 16/110 of these newborns (14.5%) had KRECs above cut-off and would not have been referred. With epigenetic immune cell counting, 16.5% (17/103) had relative B-cell counts in the range of healthy controls. Targeted NGS showed promising results as 87 out of 103 (84%) newborns with low KRECs did not show any pathogenic/likely pathogenic variants and would not have been referred for follow-up diagnostics. Conclusion: Several second-tier tests can potentially reduce the number of false-positive referrals in NBS for XLA. NGS seems to be the most effective technique in NBS for XLA and other forms of agammaglobulinemia. Our results show promising first steps towards the implementation of NBS for XLA.

### 4.16. Methods—General

P56. **Withdrawn**

P57. **NeoBase™ 2 Non-Derivatized MSMS Assay Validation on SCIEX Triple Quad™ 4500MD System**
Atte Tuominen, Sheikh Talha, Laura Koskimäki, Tuomas Leppänen, Suvituuli Poikonen, Hanna Polari and Jenny-Maria Brozinski
Revvity, Reproductive Health, Turku, Finland

Revvity NeoBase™ 2 Non-derivatized MSMS kit is capable of measuring over 50 analytes in a single assay. The kit is intended for the measurement and evaluation of amino acid, succinylacetone, free carnitine, acylcarnitine, nucleoside and lysophospholipid concentration with a tandem mass spectrometer from newborn heel prick blood specimens dried on filter paper. NeoBase 2 is an FDA-cleared IVD kit originally launched in 2018. The kit has been validated for multiple MSMS systems, and now, its performance characteristics have been studied on the SCIEX 4500MD system. The purpose of the study was to assess the performance of the NeoBase 2 assay run on SCIEX 4500MD system. This was done by studying e.g., precision, linearity, sensitivity, drift, level of carry-over of the NeoBase 2 analytes on SCIEX 4500MD. Instrument comparison study was done between SCIEX 4500MD and QSight 225 MD systems with samples across wide concentration range including leftover newborn specimen. Results demonstrated good overall performance and correlation between both tested systems.

P58. **UHPLC-MS/MS Analysis of Amino Acids in Dried Blood Spot using Waters Kairos™ Amino Acid Kit for Clinical Research**
Heather Brown, Rachel McBrinn, Padhraic Rossiter, Anahi Santoyo Castelazo, Gareth Hammond and Lisa Calton
Waters Corporation, Clinical Business Unit, Wilmslow, United Kingdom

Quantitation of amino acids from dried blood spots (DBS) can involve complex mixtures of buffers and ion-pair reagent and long analytical run times when using optical detection. In this work we describe the utility of the Kairos Amino Acid Kit for the UHPLC-MS/MS analysis of amino acids from a single DBS for clinical research. Kairos Internal Standard Solution was prepared following a modification of the care and use manual. Waters in-house and external Centre of Disease Control (CDC) DBS material were extracted and incubated with internal standard and 10 µL of each extract was transferred to a 96-well collection plate containing borate buffer. 20 µL of AccQ.Tag™ Ultra Derivatizing Reagent was added to each well and the plate was sealed and incubated for 10 min. 2 µL of sample extract was injected and analyzed using the Waters ACQUITY™ ULPC™ I-Class PLUS System and XEVO™ TQ-S micro Mass Spectrometer. Extracted DBS samples were assessed for precision, accuracy, and recovery using the Kairos Calibration Standards. Run time per sample was 10 min. Using the Kairos Amino Acid Kit calibrators, linearity for all analytes achieved an R2 ≥ 0.99. The imprecision of the method was 20% and accuracy of the DBS measurements to assigned values were within 20%. Recovery for all analytes were within 40–140%. Using the AccQ.Tag Ultra “3X” Reagent to derivatize contrived DBS extracts, the preliminary data demonstrates method analytical performance. In 10 min, the Kairos Amino Acid Kit accurately quantitates amino acids in DBS using UHPLC-MS/MS, offering clinical researchers a simple, robust, and rapid analysis without the need for mobile-phase buffers or ion-pair reagents. For Research Use Only. Not for use in diagnostic procedures.

P59. **Comparative Analysis of Derivatized and Underivatized Mass Spectrometry Techniques in Newborn Screening**
Luisa-Gabriela Bogos, Maria-Andreea Soporan, Ioana-Ecaterina Pralea, Radu-Cristian Moldovan, Sheilah Severino Snorrason, Leifur Franzson, Freyr Jóhannsson, Anca-Dana Buzoianu and Cristina-Adela Iuga
Landspitali—The National University Hospital of Iceland, Department of Genetics and Molecular Medicine, Reykjavik, Iceland, Faculty of Medicine, „Iuliu Hațieganu” University of Medicine and Pharmacy, Department of Morphofunctional Sciences, Pharmacology, Toxicology, and Clinical Pharmacology, Cluj-Napoca, Romania, MEDFUTURE—Institute for Biomedical Research Iuliu Hațieganu University of Medicine and Pharmacy, Personalized Medicine and Rare Diseases Department, Cluj-Napoca, Romania

Worldwide, newborn screening laboratories use tandem mass spectrometry (MS/MS) to analyze amino acids (AA) and acylcarnitines (AC). While the derivatized sample preparation method is widely trusted, underivatized assays are gaining interest. This study evaluates interlaboratory reproducibility between two laboratories using a butanol-HCl derivatization method, followed by a comparison of the derivatization method and an underivatized MassChrom^®^ Kit. A total of 276 DBS samples were analyzed by FIA-MS/MS in two labs—Romania and Iceland, using a butanol-HCl derivatization method. Additionally, AA and AC were extracted from 1504 DBS samples using two approaches: the derivatized in-house method and the underivatized MassChrom^®^ Kit from ChromSystems. Flow injection analysis was performed on an ACQUITY UPLC I-Class PLUS/Xevo TQ-XS IVD System. The reproducibility of the in-house method was assessed, yielding consistent analyte values. Moreover, both the derivatized and underivatized methods successfully identified abnormal values, after comparing the results obtained from the 1504 DBS samples, using individual cut-off values for each method. Furthermore, a case of elevated 3-hydroxyisovalerylcarnitine (C5-OH) was detected, highlighting the efficacy of the derivatization method in differentiating isobaric compounds such as methylmalonylcarnitine (C4DC) and C5-OH. Underivatized techniques provide a safer and more straightforward alternative to derivatization as they eliminate the use of hazardous reagents and simplify sample preparation. However, they present certain limitations, particularly in distinguishing isobaric acylcarnitines. Consequently, laboratories must evaluate these methods within their screening protocols, ensuring that the results remain reliable.

### 4.17. Methods—Metachromatic Leukodystrophy and Adrenoleukodystrophy

P.60. **Multiplexing C26:0-LPC and C16 Sulfatides Using One Punch, One Extraction and One Injection**
Min Weng, Joe Trometer, Tsun Au Yeung, Victoria Simonian, Hanna Polari and Juuso Huhtala
Revvity, Reproductive Health, Akron, United States, Revvity, Reproductive Health, Hopkinton, United States, Revvity, Reproductive Health, Turku, Finland

Metachromatic leukodystrophy (MLD) and X-linked adrenoleukodystrophy (X-ALD) are rare, inherited neurodegenerative disorders caused by deficiencies in key metabolic enzymes. MLD results from a deficiency in arylsulfatase A, leading to the accumulation of C16 sulfatides in the brain and other organs, which have recently been validated as effective biomarkers for MLD newborn screening. X-ALD is caused by pathogenic variants in the ABCD1 gene, with C26:0-lysophosphatidylcholine (C26:0-LPC) established as an accurate biomarker for that disorder. An expanding number of screened disorders poses a significant challenge for newborn screening programs, partly due to limited dried blood spot (DBS) sample material, thereby driving interest in options for multiplexing. In addition, multiplexing approaches save staff time and reduce need for additional instrumentation. The goal is to demonstrate the assay performance of the measurement of C26:0-LPC and C16 sulfatides using one punch, one extraction and one injection. We use a straightforward liquid chromatography- tandem mass spectrometry (LC-MS/MS) method capable of measuring three biomarkers, C26:0-lysophosphatidylcholine, C16:0 sulfatide and C16:1-OH sulfatide, from a single 3.2 mm DBS punch on the SCIEX 4500MD MS/MS instrument. The method was evaluated by assessing accuracy, precision, linearity range, and routine newborn DBS samples results with LC-MS/MS in negative ion mode. The overall recovery and precision (CV%) of three analytes were determined. The results demonstrate the applicability of the LC-MS/MS negative mode method for measuring C16:0 sulfatide, C16:1-OH sulfatide and C26:0-LPC concentration from a single DBS. For research use only. Not for use in diagnostic procedures.

### 4.18. Methods—Spinal Muscular Atrophy

P61. **Enhancing SMA Diagnostics: Comparing MLPA and AMPLIDEX for Accurate SMN1 and SMN2 Exon 7 Copy Number Detection**
Jera Pajnič, Karolina Mužina, Polona Lindič, Barbka Repič Lampret and Tinka Hovnik
UMC, Clinical Institute for Special Laboratory Diagnostics, Faculty of Medicine, Institute of Biochemistry and Molecular Genetics, Ljubljana, Slovenia

Spinal muscular atrophy (SMA) is a severe disorder caused by genetic variations in the SMN1 gene, leading to progressive muscle weakness and motor neuron loss. The SMN2, a homolog of SMN1, partially compensates for the loss of SMN1 function, but its copy number can vary, influencing disease severity. Early diagnosis of SMA is crucial for initiating therapy, which can significantly improve disease prognosis, particularly in newborns and infants. Reliable methods for determining the copy number of exon 7 in the SMN1 and SMN2 genes are essential for clinical diagnostics. This study compares two methods for determining the copy number of exon 7 in the SMN1 and SMN2: the SALSA MLPA Probemix P021 SMA (MRC Holland) and the AmplideX PCR/CE SMN1/2 Plus Kit (Asuragen). We tested 42 gDNA of positive and negative samples isolated from peripheral blood, determining the copy number (0, 1, 2, 3, ≥4) for both genes and evaluated the concordance between the results. Regarding SMN1 exon 7 both methods demonstrated 100% concordance across all copy number categories. For SMN2 exon 7, concordance was 100% for 0, 1, and 2 copies, but decreased to 80% for 3 copies and further dropped to 33% for ≥4 copies. The choice of method should consider the clinical context, as each has specific advantages and limitations. Our results confirm the high reliability of the Amplidex method for determining SMN1 and SMN2 copy numbers. This method offers a rapid, cost-effective screening solution, while MLPA remains the gold standard for confirming the SMA diagnosis. Although Amplidex reliably detects SMN1 copy numbers, inconsistencies in SMN2 analysis highlight the need for improved methods. To conclude, early specific confirmatory genetic testing is vital for personalized treatment and SMA management.

### 4.19. Methods—Very Long-Chain Acyl CoA-Dehydrogenase Deficiency

P62. **A New LC-MS/MS Based 2nd Tier Test Approach for Reducing VLCADD False Positive Rate in Newborn Screening**
Michela Perrone Donnorso, Michela Cassanello, Luisella Alberti, Elvira Sondo, Paola Vannini, Andrea Mascagni, Cristina Cereda, Maria Cristina Schiaffino and Mohamad Maghnie
IRCCS, Istituto Giannina Gaslini, LABSIEM—Pediatric Clinic—DINOGMI UNIGE, Genova, Italy, Buzzi Children’s Hospital, Dept. of Pediatrics—Newborn Screening Laboratory—Functional Genomics and Rare diseases, Milan, Italy

Very-long-chain acyl-CoA dehydrogenase (VLCAD) deficiency is an autosomal recessive fatty acid oxidation disorder included in most newborn screening (NBS) panels. The disorder is characterized by the increased of the primary biomarker tetradecenoyl carnitine (C14:1) and other acylcarnitines. These alterations can be also found in different non pathological conditions such as ketosis status, feeding with some specific food, or in some vitamin deficiencies (Riboflavine), and in the heterozygote state. The same abnormal pattern can also occur in decreased activities of FAD-dependent dehydrogenase involved in multiple acyl-CoA dehydrogenase deficiency (MADD) and in riboflavin metabolism. Despite the use of some strategies involving NBS acylcarnitine ratio (C14:1/C12:1 or C14:1/C16) for selecting VLCAD subjects, the improvement of positive predictive value is still a challenge. Here we propose a multiplex liquid chromatography (LC)-MS/MS 2ndtier test (2TT) that includes ketosis and MADD-like specific biomarkers. In addition, we also used the ratio of the two resolved C14:1 isomers (Cis-5 tetradecenoyl carnitine an cis 9-tetradecnoylcarnitine) that in a first instance from our observation is a promising true positives indicator. We applied our 2TT to 30 abnormal samples out of 7800 screened newborns, resulting in effectively identification of 4 VLCAD and 2 MADD deficiency and 24 false positives results including riboflavin deficiency, heterozygous and ketosis status. The preliminary results therefore indicate that the isomers of C14:1 can be used as 2TT in the selection of VLCAD-positive newborns in the NBS flowchart.

### 4.20. Methods—Adrenoleukodystrophy

P63. **Quantitative Analysis of Four Long-Chain Lysophosphatidylcholines in Dried-Blood Spots Using Liquid Chromatography**
Anahi Santoyo Castelazo, Rachel McBrinn, Gareth Hammond and Lisa Calton
Waters, Stamford Avenue, Manchester, United Kingdom, Waters, Clinical Business Unit, Manchester, United Kingdom

Historically, very long chain fatty acids have been quantified using direct transesterification and gas chromatography methods which involve time-consuming and labour-intensive sample preparation and analysis. Consequently, simpler LC-MS/MS methods have been reported for the measurement of C26:0 among other lysophosphatidylcholines (LPCs) in dried blood spot (DBS). Interferences have been reported using positive electrospray ionisation (ESI) and so researchers have developed methodologies utilising negative ESI to circumvent isobaric interferences when there is lack of chromatographic resolution. Here we describe a UHPLC-MS/MS method for the reliable analysis of four long-chain LPCs (C20:0, C22:0, C24:0 and C26:0-LPCs) in DBS for clinical research. The mobile phase composition consisted of a mixture of water, acetonitrile and isopropanol with ammonium acetate as modifier. The DBS sample extraction used a working solution prepared with the deuterated LPC internal standards in methanol. We utilized an ACQUITY™ UPLC™ I-Class PLUS System (Fixed Loop) using an ACQUITY Premier CSH™ C18 Column which provided baseline resolution of endogenous isobaric interferences for the saturated forms of the targeted LPCs. The detector was a Xevo™ TQ-S micro Mass Spectrometer operated in negative electrospray ionization mode. Method performance characterization indicated no significant carryover from a concentration of 2 uM into a consecutive blank injection, linearity was demonstrated over the range 0.1–10 µM, with a precision of ≤15% CV and bias within 20%. This method offers a simple extraction procedure and reliable quantification of C20:0, C22:0, C24:0 and C26:0 LPCs in DBS for clinical research. For Research Use Only. Not for use in diagnostic procedures.

### 4.21. Methyl Malonic Aciduria

P64. **Mutation Profile of the MUT and MMACHC Gene in Center-South Chinese Methylmalonic Aciduria Patients**
Jun He, Huiming Yan and Xia Li
Hunan Provincial Maternal and Child Health Care Hospital, Department of Medical Genetics, changsha, China, Peoples Republic, changsha Hospital for Maternal and Child Health Care,, Department of Genetics and Eugenics, changsha, China, Peoples Republic, changsha

Background: Methylmalonic acidemia (MMA) comprises a series of autosomal recessive inherited disorders of organic acid metabolism, which causes various clinical symptoms including recurrent vomiting, metabolic acidosis, and even developmental delay. In the present study, we evaluated the clinical, biochemical and molecular characteristics of patients with MMA harboring MUT and MMACHC mutations in Hunan province. Methods: During the study, 1,023,166 newborns were screened by tandem mass spectrometry (MS/MS) in the Hunan Provincial Maternal and Child Health Care Hospital. C3, C3/C2 and methionine were measured during the first screening. Homocysteine (HCY) was measured in the second round, blood samples from the infants and/or their family members were used for DNA analysis. The entire coding regions of the MMACHC and MUTgenes associated with MMA were sequenced by next-generation sequencing (NGS). Results: Eight patients with MMACHC mutations and six patients with MUT mutations were identified among the 1,023,166 screened newborns; the estimated total incidence of MMA was 1:73,083 in Hunan. Among the MMA patients, only one died of metabolic crisis after birth. All the patients identified had two mutant alleles except for one individual with early-onset disease. The most common MMACHC mutation was c.609G A (p.W203*). Conclusion: Our study reports the clinical characteristics of MMA and diagnosis through MS/MS and NGS. There is a slightly higher incidence of MMA with homocysteinemia than isolated MMA in Hunan province. Our findings also enlarge the spectrum of mutations in the MUT gene.

### 4.22. Maple Syrup Urine Disease

P65. **MSUD Detected and Missed by Newborn Screening in Norway**
Trine Tangeraas, Olve Moldestad, Asbjørg Stray-Pedersen, Janne Strand, Yngve Thomas Bliksrud, Kiarash Tazmini, Cathrin Lytomt Salvador, Erle Kristensen, Magnus Odin Resløkken Dahlseng, Julie Hellem-Aaaby, Marit Brynjulvsen, Siv Merete Løvoll, Andreas Øberg and Berit Woldseth
Oslo University Hospital, Department of Medical Biochemistry, Oslo, Norway, Oslo University Hospital, Department of Newborn Screening, Oslo, Norway, Oslo University Hospital, Department of Endocrinology, Oslo, Norway, Oslo University Hospital, Newborn screening, dep of Paediatric and adolescent medicine, Oslo, Norway, Oslo University Hospital, Department of Medical Biochemistry, Oslo, Norway

Classical Maple Syrup Urine Disease (cMSUD) is readily detected by newborn screening (NBS). Intermittent MSUD (iMSUD) can be challenging to identify both clinically and by NBS. This retrospective study explored the performance of NBS for MSUD implemented in 2012 and included iMSUD cases ascertained between 1978 and 2024 in Norway. Dried blood spots (DBS) were sampled 48–72 h after birth.1sttier combined analysis of allo-isoleu, leu, isoleu, and OH-proline was designated as XLE. 2nd tier allo-isoleu and DBS molecular analysis of relevant genes were applied in 2016–2017. Of 715,000 screened in 2012–2024, 11 cases of MSUD were ascertained (1:65,000). Five cases were detected by NBS (1:143.000); cMSUD (*n* = 3) and iMSUD (*n* = 2) and *n* = 2 were false positive. Six cases of iMSUD (1:119.000) were missed by NBS; three were diagnosed following a metabolic crisis at age 1.3–8 years, with a fatal outcome in one case. Three asymptomatic siblings were diagnosed aged 0.5–6.7 years. The original NBS DBS result of the six missed iMSUD children revealed median XLE 155 µmol/L (72–179), cut-off 250 µmol/L, valine 131 µmol/L (71–161), cutoff 250 µmol/L and XLE/Ala median 0.52 (0.43–0.62), cut-off 1.5. Ten iMSUD cases were identified prior to NBS. Overall, 18 cases of iMSUD were diagnosed from 1978 to 2024. The most frequent gene variants were c.901 C T (11/36) in DBT and c.263A T (8/36) in BCKDHB. NBS failed to detect 6/8 (75%) of newborns with iMSUD. Alternative biomarkers are needed to improve NBS sensitivity for iMSUD. Alternatively, although more resource-intensive, qPCR multiplexing or molecular analysis of frequent gene variants could be considered as a 1st tier approach.

P66. **Explorative Algorithm Using MS/MS to Identify Intermittent MSUD through Newborn Screening in Norway**
Siv Merete Løvoll, Marit Brynjulvsen, Alexander D. Rowe, Asbjørg Stray-Pedersen, Janne Maren Strand, Ingjerd Sæves, Olve Moldestad and Trine Tangeraas
Oslo University Hospital, Newborn screening, Oslo, Norway

Primary screening of MSUD (1sttier) using Tandem Mass Spectrometry (MS/MS) was implemented in the Norwegian Newborn screening (NBS) program in 2012, and confirmatory testing with molecular and biochemical analysis in 2016 and 2017, respectively. Our 1sttier quantitate XLE isomers include Allo-Isoleucine (Allo-Ile), Isoleucine, Leucine and Hydroxyproline, Valine (VAL) and XLE/Alanine. This method cannot distinguish between isobaric compounds, thus, chromatographic separation to quantitate the XLE is required to reduce false positive MSUD. Our MSUD 2nd tier MS/MS quantitate the branched chain amino acids and Allo-Ile, and when abnormal, the neonate is referred prior to molecular testing. Although current 1st tier readily detects classic MSUD (cMSUD), the algorithm fails to detect most cases of intermittent MSUD (iMSUD), which in Norway is more prevalent than cMSUD. By reevaluating the existing data from MSUD-positive samples (2012–2024) and comparing with 50,473 MSUD-negative controls (2024), we aimed to improve the sensitivity of 1sttier for iMSUD. In total 11 individuals were diagnosed (1:65,000) during 2012–2024. Five MSUD were identified (3 cMSUD and 2 iMSUD (1:143,000)) and the remaining six iMSUD (1:119,000) were false negative and diagnosed clinically (75%). To enhance sensitivity in the 1st tier, we propose an explorative algorithm consisting of following markers XLE, VAL, C5, C5/C3, Methionine/XLE, VAL/Phenylalanine (PHE) and Leucine/PHE. This algorithm identified four out of six false negative iMSUD. In addition, two previous false positive MSUDs were stratified correctly, improving specificity. These promising results support future implementation of the algorithm in 1sttier analysis of MSUD followed by molecular testing.

### 4.23. Overview-Review

P67. **Barriers to Standardisation in Newborn Screening for Rare Diseases in Europe: A Systematic Literature Review of the Evidence**
Debra De Silva-Sun
Lancaster University, Division of Health Research, 4055 Basel, Switzerland

Despite the widespread adoption of NBS initiatives across Europe, significant methodological differences exist, leading to disparities in accessibility and outcomes. These discrepancies arise from variations in the range of disorders included in NBS programs, often shaped by healthcare budgets and the level of evidence required to justify screening (Loeber et al., 2021; Sikonja et al., 2022). While early interventions enabled by NBS have demonstrated positive impacts on clinical outcomes, reductions in health inequalities remain limited (Schlüter et al., 2020). The inclusion of conditions in NBS panels is largely influenced by local epidemiology, resource constraints, and political priorities, rather than systematic, evidence-based criteria (Taylor-Phillips et al., 2018). This lack of standardization hampers data sharing and the adoption of best practices, creating barriers to equitable access and the optimization of NBS programs. This review examines the evidence and identifies barriers to standardizing the number of rare diseases screened in NBS panels across Europe. We hypothesize that initiating the diagnostic journey at birth offers a unique opportunity to promote equity in healthcare. Implementing equitable NBS programs requires robust clinical data, availability of effective therapies, and alignment with healthcare system capabilities, balanced against considerations of societal resource allocation (Cornel et al., 2011; Mooney, 2003).

### 4.24. Pilot—NBS

P68. **Outcome from a Pilot Newborn Screening Program in a Coastal District of South India**
Sudheer Moorkoth, Leslie Lewis and Krishnananda Prabhu
Manipal College of Pharmaceutical Sciences, Pharmaceutical Quality Assurance, Manipal, India, Kasturba medical College, pediatrics, Manipal, India,, Kasturba Medical College, Biochemistry, Manipal, India

A pilot newborn screening (NBS) project was undertaken to study the feasibility and challenges of implementing NBS in the Udupi district of South India. The project was funded under the Grand Challenges Canada scheme. We evaluated the awareness of various stakeholders on NBS and inborn metabolic disorders through questionnaire. We screened six disorders such as congenital hypothyroidism (CH), congenital adrenal hyperplasia (CAH), glucose6-phosphate dehydrogenase deficiency (G-6PDD), biotinidase deficiency (BTD), phenylketonuria (PKU) and galactosemia (GALT) for a period of 18 months covering 8115 babies born during this period. A cost benefit analysis was performed based on the results and the expenses incurred during the study. The awareness level among the participants on NBS and IEM was only 30% at the start of the study. However, the survey conducted after awareness-creation activities showed a significant increase to 98%. The observed incidence rate for these disorders were CH (1: 811), CAH (1:2009), G-6PDD (1:932), BTD (1:1475), GALT (1:1340), PKU (No incidence observed) in our study. The cost benefit analysis revealed that the benefit outweighs the cost as seen by the high benefit to cost ratio for CH (6.91), CAH (17.35), and G-6PDD (26.07). The outcomes of this study was that, upon presenting our data to the local government administration of Udupi district, an NBS programme for CH and CAH was initiated to the government hospitals of Udupi, through a public-private partnership. Also, the private hospitals attached with our university implemented the program with a cost to the parents. This pilot screening model could be initiated for every state of India so that a tailored NBS can be implemented for the very diverse population of India.

P69. **Selective Neonatal Screening for Inherited Metabolic Disorders Using Tandem Mass Spectrometry in Kazakhstan**
Gulnara Svyatova, Alexandra Murtazaliyeva, Anastassiya Ge, Vladislav Sklyarov and Madina Amantayeva
Pribori Oy, Almaty, Kazakhstan, LLP Center of Molecular Medicine, Medical-Genetic Counseling with a Medical Laboratory, Almaty, Kazakhstan, JSC “Scientific Center of Obstetrics, Gynecology and Perinatology”, Republican Medical-Genetic Consultation, Almaty, Kazakhstan, JSC “Scientific Center of Obstetrics, Gynecology and Perinatology”, Republican Medical-Genetic Consultation, Almaty, Kazakhstan, LLP Center of Molecular Medicine.

Introduction. According to the United Nations World Population Outlook 2019 report, hereditary metabolic diseases (IMD) account for at least 33% of deaths among children under the age of five worldwide. Newborn screening is the most effective strategy for reducing childhood disability and mortality through early diagnosis and timely intervention before clinical symptoms appear. Objectives. This study aimed to study the prevalence and structure of IMD among newborns in the Republic of Kazakhstan and establish reference standards for metabolites. Methods. Selective screening was conducted on 4670 children at the Center for Molecular Medicine LLP using QSight Perkin Elmer equipment, the NeoBase™ MSMS kit reagents, and the NeoLSD™ MSMS kit. Data analysis was performed using Simplicity 3Q Analyzer software, while statistical processing of the collected data was conducted using Stata 16. Results. 117 IMD cases were detected, corresponding to a cumulative incidence of 25.1 per 1000 examined. Of these, seven children (6.0%) had died at the time of the analysis. All diagnosed children received an accurate genetic diagnosis and pathogenetic treatment, primarily diet therapy aimed at limiting the consumption of substrates and normalizing metabolism. The screening allowed us to develop our reference standards for metabolites, determine the prevalence and structure of IMDs, and devise a method for providing medical care to patients with IMDs. Conclusion. Early detection of rare IMD plays a key role in reducing infant and child mortality, morbidity, and disability. In addition, it reduces the economic burden associated with prolonged symptomatic treatment, ongoing care, and reduced quality of life for children with these diseases.

P70. **Outcome of 2-year Expanded Newborn Screening Program in Ukraine**
Oksana Barvinska, Nataliia Mytsyk, Yulia Zhyvytsia, Iryna Hrehul, Svetlana Kormoz, Olena Kutsyk, Marina Patsora, Mariia Haidei, Tetiana Shklyarska, Yulia Ostapyshyna, Volodymyr Olkhovych, Alexandr Buryak, Andrii Lipatnikov, Galina Makuch, Nataliia Samonenko, Nataliia Olkhovych, Mykola Veropotvelyan, Olena Grechanina, Tetiana Ivanova and Nataliia Gorovenko
National Children’s Hospital OHMATDYT, Laboratory of Medical Genetics, Kyiv, Ukraine, National Children’s Hospital OHMATDYT, Center of Orphan Diseases and Gene Therapy, Kyiv, Ukraine, National Scientific Centre, Institute of Genetic and Regenerative Medicine, Department of Genetic Diagnostics, Kyiv, Ukraine, National Children’s Hospital OHMATDYT, Neonatal Intensive Care Unit, Kyiv, Ukraine, Lviv Perinatal Center, Lviv Regional Center of Neonatal Screening, Lviv, Ukraine, Interregional Centre for Medical Genetics and Prenatal Diagnostics, Kryvyi Rig, Ukraine, Regional Center of Neonatal Screening, Kryvyi Rig, Ukraine, Kharkiv Specialised Medical and Genetic Centre, Kharkiv Regional Center of Neonatal Screening, Kharkiv, Ukraine

Background: Newborn screening is a public health program for early detection and management of certain inherited diseases. The nationwide expanded newborn screening (ENS) program in Ukraine includes 21 congenital disorders (CD) and is conducted by 4 screening centers (SC). It was implemented in Ukraine in two steps: pilot project started in Lviv and Kyiv SC (October 2022) and then Kharkiv and Kryvyi Rig SC joined to ENS (April 2023). Today, ENS in Ukraine covers all unoccupied regions of Ukraine. Methods: DBS, LC-MS/MS, RT-PCR, IFA, GC-MS, NGS, Sanger sequencing, and MLPA. Results: In 2 years, 354 696 newborns were screened and 359 newborns with CD were confirmed. The coverage of the ENS program in unoccupied regions of Ukraine is 98%. Among the most common screened diseases have been detected congenital hypothyroidism (CH) (86; 24%), phenylketonuria (76; 21.2%), spinal muscular atrophy (51; 14.2%) and cystic fibrosis (47; 13.1%). Of the screened inborn metabolic disorders, the highest prevalence was observed among the following: galactosemia Duarte (21; 5.8%), MCADD (7; 1.9%), glutaric aciduria type I (5; 1.4%), carnitine uptake deficiency (4; 1.1%), galactosemia type 1 (4; 1.1%). The congenital immunodeficiencies were confirmed in 13 patients (3.6%). The total incidence rate of screened CD in Ukraine is 1 in 988. False negative results were reported for 2 CH cases. Conclusion: The implementation of ENS in Ukraine highlights improving orphan patients’ medical care by early diagnosis and treatment.

P71. **Pilot Study of Newborn Screening for Inborn Errors of Metabolism Using Tandem Mass Spectrometry in Romania**
Maria Andreea Soporan, Luisa Gabriela Bogos, Radu Cristian Moldovan, Ioana Ecaterina Pralea, Carmen Costache, Floredana Laura Sular, Florin George Horhat, Iuliana Scrob, Silvia Scrob, Gabriela Corina Zaharie, Diana Miclea, Romana Vulturar, Anca Dana Buzoianu and Cristina Adela Iuga
MEDFUTURE—Institute for Biomedical Research Iuliu Hațieganu University of Medicine and Pharmacy, Personalized Medicine and Rare Diseases Department, Cluj Napoca, Romania, Cluj County Emergency Clinical Hospital, Cluj-Napoca, Romania, Central Laboratory, Regional Screening Program, Cluj Napoca, Romania, Emergency Clinical County Hospital, Târgu Mureș, Romania, Central Laboratory, Regional Screening Program, Targu Mures, Romania, Emergency Hospital for Children Louis Turcanu, Timisoara, Romania, Central Laboratory, Regional Screening Program, Timisoara, Romania, Department of Mother and Child, Neonatology, Cluj Napoca, Romania, Department of Molecular Sciences, Cellular and Molecular Biology, Cluj Napoca, Romania, Faculty of Medicine, „Iuliu Hațieganu” University of Medicine and Pharmacy, Cluj-Napoca, Romania, Department of Morphofunctional Sciences, Pharmacology, Toxicology, and Clinical Pharmacology, Cluj Napoca, Romania

Newborn screening (NBS) using tandem mass spectrometry (MS/MS) is a cutting-edge technique that enables early diagnosis of nearly 50 inherited metabolic diseases from a single dried blood spot (DBS). In Romania, NBS was introduced in the late 1990s with phenylketonuria (PKU) screening using a fluorometric method. Over time, it became part of the National Screening Program, which now includes testing for PKU, congenital hypothyroidism, and more recently, cystic fibrosis. Between November 2023 and April 2024, a total of 20,092 newborns from 11 counties were screened for amino acid, organic acid, and fatty acid oxidation disorders. DBS samples were provided by 3 out of 5 Regional Screening Laboratories and processed using the ChromSystems MassChrom^®^ underivatized kit. The extracted metabolites were analyzed via flow injection on an ACQUITY UPLC I-Class with a Xevo TQ-XS IVD System. Specific cut-off values were determined for each compound. The most common findings included low free carnitine, elevated propionyl-carnitine, and elevated phenylalanine levels. Two cases of PKU were confirmed, and one was identified as a false positive. A total of 275 samples were from known premature infants, for whom a second sample could not be provided. This study represents the first extended NBS pilot project in Romania, highlighting the potential of mass spectrometry to improve early detection of inborn errors of metabolism. However, significant limitations, including the lack of detailed information for each newborn and the inability to collect follow-up samples, particularly from premature infants, impacted the effectiveness of the screening process. Addressing these challenges is critical for the successful implementation of a comprehensive NBS program in Romania.

P72. **Burden of Inherited Metabolic Disorders in the Era of Expanded Newborn Screening: A Study from Pakistan**
Azeema Jamil, Lena Jafri, Sibtain Ahmed, Aysha Habib Khan, Nasir Ali Khan and Hafsa Majid
The Aga Khan University Hospital, Chemical Patholgy Clinical Laboratories, Karachi, Pakistan

In Pakistan, diagnosing rare hereditary metabolic diseases has been challenging due to limited resources and expertise. This study aims to perform newborn screening (NBS) for hereditary metabolic disorders, focusing on 12 genetic metabolic disorders, using LC-MS/MS to assess amino acids and acylcarnitine level. Method: Newborn blood samples were collected between 33–72 h after birth using heel prick on filter paper to create dry blood spots (DBS). Sample analyzed to detect metabolic disorders of Maple Syrup Urine Disease, Citrullinemia, Argininemia, Homocystinuria, Methylmalonic Aciduria, Propionic acidemia, Glutaric Aciduria, Isovaleric Acidaemia, Argininosuccinic aciduria and Cobalamin intracellular defects (Cobalamin-A, B, C, D, F, TC-II). Result: Total 6812 samples analyzed (August 2023–December 2024). 4% had a history of blood transfusion and 34% were on total parenteral nutrition. Gestational ages ranged from 25 to 40 weeks with birth weights between 0.5–4.0 kg. Screening identified 261 high results, 57% (*n* = 150) were male and positives were C3-Carnitine 42% (*n* = 110), C5-Carnitine 17% (*n* = 45), C5DC-Carnitine 6% (*n* = 15), methionine 16% (*n* = 41), leucine 14% (*n* = 36), arginine 3% (*n* = 9) and citrulline 2% (*n* = 6). If results exceed cutoffs physician referred for confirmatory tests of organic acids and amino acid analysis. Two true positive cases, Homocystinuria and citrullinemia were confirmed. Conclusion: The study screened 6812 newborns, identifying metabolic abnormalities in a small percentage. The confirmed incidence of metabolic disorders was 1:6812. The results emphasize the need for establishing independent cut-off for neonatal screening, which would improve diagnostic efficiency for metabolic diseases in Pakistan.

P73. **Expanded Newborn Screening for Inborn Errors of Metabolism in Greece: One Year Experience of the National Newborn Screening Program**
Dimitrios Platis, Vasiliki Gkioni, Maria Gounaropoulou, Anna Gkika, Xrisavgi Finitsi, Vasilia Dremetsika, Aggeliki Louloudaki, Aggeliki Charou and Panagiotis Girginoudis
Institute of Child Health, Department of Newborn Screening, Athens, Greece

Newborn screening (NBS) is an extremely important public health initiative aiming at identifying pre-symptomatic newborns that will develop significant disease if left undiagnosed and untreated. In Greece, the National Newborn Screening Program, initiated in 1974, is performed by the Institute of Child Health (ICH). Recently, the existing newborn screening program which up to that point screened for 4 conditions was expanded to include amino aminoacidopathies, fatty acid oxidation disorders (FAOD), and organic acid metabolic disorders, as well as, cystic fibrosis. Flow injection analysis-electrospray ionization-tandem mass spectrometry (FIA-ESI-MS/MS) was applied for the expanded MS panel of disorders, while for cystic fibrosis immunoreactive trypsinogen (IRT) measurement was applied (time-resolved fluoroimmunoassay). In the period between November 2023 and December 2024, over 90,000 newborns were screened for the normal panel plus cystic fibrosis and over 50,000 for the expanded MS panel. Cutoff values for the measured metabolites were established and the respective algorithms for each disorder optimized. During this period 13 infants were identified with cystic fibrosis, 1 with MCADD, 4 with VLCADD, 1 with HMGD and 8 with phenylketonuria/mild phenylketonuria/hyperphenylalaninemia.

### 4.25. Pilot—Severe Combined Immunodeficiency and Spinal Muscular Atrophy

P74. **Pilot Study: Newborn Screening for SMA, SCID and XLA in Slovenia**
Polona Lindič, Jera Pajnič, Žiga Iztok Remec, Tinka Hovnik, Maja Ficko, Jernej Kovač and Barbka Repič Lampret
University Medical Center Ljubljana, University Children’s Hospital, Clinical Institute of Special Laboratory Diagnostics, Ljubljana, Slovenia

Spinal Muscular Atrophy (SMA) is a genetic neuromuscular disorder causing muscle weakness and paralysis. Severe Combined Immunodeficiency (SCID) and X-linked Agammaglobulinemia (XLA) are genetic conditions that impair the immune system, increasing infection risk. Early diagnosis is crucial, as treatment can slow or stop disease progression. During pilot study in Slovenia 7850 newborns were tested for SMA, XLA, and SCID, using the SPOT-it™ TREC, KREC; SMN1 Screening Kit (ImmunoIVD). Five newborns needed confirmatory diagnostics. One child tested positive for SMA. Result was verified using the AmplideX^®^ PCR/CE SMN1/2 Plus Kit (Asuragen) on the primary sample. Testing with MLPA using the SALSA MLPA Probemix P021 SMA (MRC Holland) on a new blood sample confirmed a homozygous deletion of exon 7 in the SMN1 gene, with two copies of the SMN2 gene. At the time of diagnosis, the child was asymptomatic and received Zolgensma at 23 days old, becoming the youngest child in Slovenia to receive this therapy. No false-positive results for SMA were recorded. Two children had reduced TRECs and two had reduced KRECs values. Both children with reduced TRECs were preterm, one also having Down syndrome and chylothorax. For the children with reduced KRECs, factors such as maternal immunosuppressive therapy and Parvovirus B19 infection during pregnancy were identified as potential influences on the results. During the pilot study, no newborns were confirmed for SCID or XLA. About 3.6% of samples had result determination issues. We found that the issues were related to the EDTA-coated capillaries used in certain maternity hospitals. This was resolved by using capillaries without PCR inhibitors. Due to the success of this study we officially launched the screening program in March 2024.

P75. **Newborn Screening for Spinal Muscular Atrophy and Inborn Errors of Immunity in The Czech Republic: A 3-Year Single Center Experience**
Karolina Peskova, Anna Sediva, Adam Klocperk, Marketa Bloomfield, Petra Hedvicakova, Emilie Vyhnalkova, Jakub Hodik, Petr Chrastina, Lenka Dvorakova and Jana Haberlova
General University Hospital in Prague and First Faculty of Medicine, Charles University, Department of Pediatrics and Inherited Metabolic Disorders, Prague, Czech Republic, 2nd Faculty of Medicine, Charles University and University Hospital in Motol, Institute of Biology and Medical Genetics, Prague, Czech Republic, 2nd Faculty of Medicine, Charles University and University Hospital in Motol, Department of Child Neurology, Prague, Czech Republic

In 1/2022, we launched 2-year pilot program to screen spinal muscular atrophy (SMA) and inborn errors of immunity, including severe combined immunodeficiency (SCID) and primary agammaglobulinemia. More than 90% of newborns were tested. In 1/2024, the pilot program has successfully transitioned into the national newborn screening program. We used multiplex real-time PCR for the identification of homozygous deletions encompassing exon 7 in the SMN1 gene; the quantification of T-cell receptor excision circles (TREC) and Kappa-deleting recombination excision circles (KREC). Positive screening outcomes underwent validation through complementary methods, incl. flow cytometry, MLPA, sequencing, microarray analysis. In the Prague center 168,160 newborns were screened in 2022–2024. 26 patients were diagnosed with SMA; therapy was provided to 21 patients with SMN2 gene copy number 5. Two patients with severe symptoms at birth were not treated and died at 3 months of age. Three patients with 4 SMN2 copies are not treated. Deficient TREC levels in 44 newborns led to the identification of SCID in 3 cases, DiGeorge syndrome in 3 cases, ataxia telangiectasia and CHARGE syndrome in 1 case each, while other instances were attributed to congenital infection (3×) or severe prematurity (14×). Deficient KREC detected in 68 newborns led to diagnosis of X-linked agammaglobulinemia (BTK gene, 5×), agammaglobulinemia associated with IGLL1 or TCF3 (4×) and was secondary to immunosuppressive treatment of the mother (10×). One newborn with deficient TREC and 1 newborn with deficient KREC were not explained. Three years of testing confirmed the predicted incidence in SMA and SCID, the incidence of agammaglobulinemia was surprisingly 6x higher. Funding: MH CZ-DROVFN64165

P76. **Newborn Screening for Spinal Muscular Atrophy in Sweden—Results from the First 18 Months**
Rolf. H Zetterström and Veroniqa Lundbäck
Karolinska University Hospital Box 4027, Centre for Inherited Metabolic Diseases, Solna, Sweden, Department of Molecular Medicine and Surgery, Karolinska Institute, Stockholm, Sweden

The most severe types of spinal muscular atrophy (SMA) was recently added to the Swedish newborn screening (NBS) program. Here, we report results of SMN1 and SMN2 copy number variants in 27 SMA patient NBS samples analyzed in our pre-screening validation study and outcome from the first 18 months of SMA screening. SMA screening is performed using a commercially available real-time PCR multiplexed assay for SMN1, TREC and ACTB detection. The assay identifies newborns with a homozygous SMN1 exon 7 deletion and is used as first tier analysis in our screening algorithm. Abnormal samples are further analyzed with a second tier ddPCR assay to determine SMN2 copy number, to better discriminate between different SMA forms, only identifying newborns eligible for immediate treatment. From 30 August 2023, to 30 January 2024, approximately 150,000 newborns were screened. Seven newborns with a homozygous deletion of SMN1 exon 7 and up to 3 copies of the SMN2 gene were found. Three of the infants had 3 copies of SMN2, and four had 2 copies of SMN2. Our screening algorithm provide a rapid and robust screening assay to pick up the most severe forms of SMA facilitating clinical follow-up and early treatment of these children.

### 4.26. Phenylketonuria

P77. **Neonatal Screening for Phenylketonuria in North Macedonia by Fluorescent Ninhydrin Method**
Milica Pesevska, Violeta Anastasovska, Nikolina Zdraveska and Dijana Plaseska-Karanfilska
Macedonian Academy of Sciences and Arts, Research Center for Genetic Engineering and Biotechnology “Georgi D. Efremov”, Skopje, North Macedonia, University Clinic for Pediatrics, Ss.Cyril and Methodius University in Skopje, Faculty of Medicine, Department for neonatology, Skopje, North Macedonia, University Clinic for Pediatrics, Ss.Cyril and Methodius University in Skopje, Faculty of Medicine,, Department for neonatal screening, Skopje, North Macedonia

Deficiency of hepatic phenylalanine hydroxylase activity increases levels of phenylalanine (Phe) in blood leading to its accumulation in all parts of the body including brain, causing phenylketonuria (PKU). The newborn screening for PKU in North Macedonia started as a pilot study during the period July 2022–March 2024 and became national mandatory screening after that. The phenylalanine level was measured from dry blood spots collected 48–72 h after birth on filter paper using fluorescent ninhydrin method. The Phe values above 182 µmol/L were taken as positive. Final diagnosis was done by molecular testing on PAH (phenylalanine hydroxylase) gene. During the period July 2022–January 2025, a total of 29.627 newborns were screened. Twenty-one newborns were called for second PKU test and recall rate of 0.07 was estimated. Seven of them had positive second screening test, three newborns were diagnosed as classic PKU and the other four as hyperphenylalaninemia by direct exons sequencing of the PAH gene. The most common mutation was c.143T C p.(Leu48Ser), accounting for 5/14 (35.7%) of the alleles, followed by the c.1208C T p.(Ala403Val) on 3/14 (21.4%) and c.898G T p.(Ala300Ser) on 2/14 (14.3%) of the alleles. The following mutations: c.1169A G p.(Glu390Gly), c.842C T p.(Pro281Leu), c.1066-11G A and c.442-5C G were identified on single allele 1/14 (7.1%), respectively. In North Macedonia was estimated the incidence of 1/9876 for classic PKU and 1/7407 for hyperphenylalaninemia. Neonatal screening allows early treatment with Phe-restricted diet therapy preventing intellectual disabilities development and mental retardation giving PKU patients quality life. Key words: Newborn screening, PAH gene, Phenylalanine

P78. **Phenylalanine Range Adjustment: Georgian Experience**
Dodo Agladze, Nino Vardosanidze and Lali Margvelashvili
Medical genetics and laboratory diagnostic center, clinical genetics, Tbilisi, Georgia Republic, Tbilisi State Medical University, General pediatrics, Tbilisi, Georgia Republic

Introduction: Phenylketonuria (PKU) is one of the most frequent autosomal-recessive metabolic disease. If untreated, PKU will cause a mental developmental delay and the seviere neurological symptoms. In Georgia mandatory screening for PKU was implemented in 2003. Method: At the end of 2019 a specialized working group was established, and statistical analysis of neonatal screening was performed. Data of all newborns who participated in PKU screening 2003–2019 was collected. It included screening Phe results, secondary Phe results, diagnosed PKU patients and late diagnosed patient’s data. In 2020, by the decision of the working group, the phenylalanine threshold for screening was adjusted from 3.2mg/dL to 2.5 mg/dL. Data obtained in 2003–2019 were compared with the same data of 2020–2024 y. Results: Between 2003–2019, 847,236 newborns were screened in Georgia. During this period, the Phe normal range was considered 3.2 mg/dL. During above mentioned period 141 cases of PKU were identified, but 22 PKU cases were missed by newborn screening. Based on working group’s decision, the phenylalanine threshold was lowered to 2.5 mg/dL. Between 2020 and 2024, after renewed normal range was introduced, newborn screening identified 82 new PKU cases. Compared to the period from 2015 to 2019 (5 y), the number of diagnosed cases in 2020–2024 (5 y) increased by 47.6%. Conclusion: Lowering the phenylalanine threshold from 3.2 mg/dL to 2.5 mg/dL gave the opportunity to identify nearly twice as many PKU patients, significantly reducing the risk of undiagnosed cases. This data underscores the importance of country specific threshold for Phe. Because the screening process can be affected by many factors, further research is necessary.

P79. **The First PKU Register And Its Value**
Kate Hall
International Society for Neonatal Screening, 3607HP Maarssen, Netherlands

Background Information relating to the diagnosis, IQ and progress of all phenyketonuric patients born in the United Kingdom, UK, on and after 1 January 1964 was developed into the phenyketonuria, PKU, register for the UK commencing in 1965. The register was set up and maintained by Dr Frederick Hudson, a paediatrician at Alder Hey Children’s Hospital, Liverpool. The register was initially designed to assess the efficiency of the screening programme based on urine testing using a modification of the ferric chloride test and whether early treatment of PKU produced the benefits being claimed at that time. Evolution and findings Around 1972, it was ascertained that over 100 names had not been notified to the register. Since then, all newborn screening laboratories in the UK reported names of infants with persistently raised phenylalanine to the register thereby ensuring all known cases were recorded. It became clear that urine screening between 3 and 6 weeks of age missed cases of PKU and probably led to late treatment. This method was gradually replaced by blood testing using the Scriver, Guthrie or McCaman and Robins methods between 6 and 14 days of age. The change was complete by 1971. Cases of late treatment diminished rapidly. In 1976 the files were moved to London. Based on IQ assessments, blood phenylalanine concentrations and behavioural monitoring recorded by the register, in 1993 the British Medical Research Council was able to produce national recommendations on the dietary treatment of PKU, written by Dr Smith. Conclusion The value of disease registers in long term outcome studies is served best by collaboration of newborn screening laboratories and paediatricians to ensure their completeness and utility.

### 4.27. Severe Combined Immunodeficiency

P80. **Current Situation of Universal Newborn Screening Programs for Severe Combined Immunodeficiency in Spain**
Ana Argudo-Ramírez, AECOM’ NBS Working Group—Pere Soler-Palacín, Lola Rausell-Félix, Elena Llorente-Martín, Alba Berzal-Serrano, Raquel Yahyahoui, Beatriz de Felipe Carmen Delgado-Pecellin, Alejandra González-Delgado, Cristobal Colón-Mejeras, Andrea Martín-Nalda, Marta Piedelobo and Judit García-Villoria
Hospital Clínic, Errors Inborn of Metabolism, Center for Biomedical Research Network on Rare Diseases (CIBERER), Biomedical Research Institute, August Pi i Sunyer (IDIBAPS), Barcelona, Spain, AECOM, NBS Working Group in Spain, -, Spain, Hospital Clínic, Errors Inborn of Metabolism, Barcelona, Spain, H. Gregorio Marañon, Laboratory Medicine, Madrid, Spain, Vall d’Hebron Hospital, Pediatric Infectious Diseases and Immunodeficiencies Unit, Barcelona, Spain, Santiago de Compostela University Clinical Hospital, Unit for Diagnosis and Treatment of Congenital Metabolic Disorders, Santiago de Compostela, Spain, Canarias University Hospital Complex, Canarias Newborn Screening Unit, Canary Islands, Spain, Virgen del Rocío University Hospital, Western Andalucía Newborn Screening Laboratory, Sevilla, Spain, Hospital Universitario Virgen del Rocío, Institute of Biomedicine of Seville, Infectious Diseases, Rheumatology and Immunology Unit, Sevilla, Spain, Málaga Regional University Hospital, Eastern Andalucía Newborn Screening Laboratory, Málaga, Spain, Health Research Institut La Fe (IIS La Fe, Molecular, Cell and Genomics Biomedicine Team, Valencia, Spain, La Fe Hospital, Valencian Newborn Screening Laboratory, Valencia, Spain, Gregorio Marañón Hospital, Department of Laboratory Medicine, Madrid, Spain

Several published studies support the cost-effectiveness of neonatal screening (NBS) for severe combined immunodeficiency (SCID). Despite this, it has not yet been incorporated in Spain into the Primary-Panel of Services of the National Health System (NHS). Since each Autonomous Community (CCAA) have the competences in Health, it is possible to incorporate it into their Secondary-Panel. For this reason Catalonia began to screen SCID in 2017, being a pioneer in Spain and Europe. The aim of the present work is to document the current situation of universal SCID NBS programs in Spain, consisting of 17 CCAA and 2 autonomous cities, with 15 NBS laboratories. SCID NBS is officially implemented in Catalonia (2017, *n* = 474,656), Galicia (2023, *n* = 14,397), Canary-Islands (2023, *n* = 19,554) and Castilla-La-Mancha (NA). Pilot studies have been conducted in Western-Andalusia (2013, *n* = 8814; 2017, *n* = 1400 and 2021, *n* = 14,035), Eastern-Andalusia (2022, *n* = 35,000), Valencian-Community (2023, *n* = 17,654) and Community-of-Madrid (2023, *n* = 59,630). All CCAA in Spain are in advanced conversations with Government, some with an expected start date. Since 2017, approximately 645,140 newborns have been screened for SCID in Spain. Eleven newborns were detected through screening (1:58,649) and benefited from early treatment (unfortunately, none through the pilot studies); so far, no cases have been reported as false negative. The current picture of SCID NBS in Spain is heterogeneous, mainly because SCID is not included in the NHS Primary-Panel. However, it is expected to be included during the first semester of 2025, and all CCAA are already lined up to be able to start as soon as it is officially published.

P81. **Combined Screening of SCD XLA and SMA in Neonates by Multiplex qPCR in China**
Rulai Yang, Chi Chen, Jianbin Yang and Chao Zhang
Children’s Hospital Zhejiang University School of Medicine, National Clinical Research Center for Child Health, Genetics &Metabolism Department, Hangzhou, China, Peoples Republic

Objective: To explore the feasibility of screening SCID XLA and SMA in newborns by multiplex qPCR technology, and provide evidence for early screening, diagnosis and treatment of children in China. Methods: From July 2021 to January 2023, 103,240 cases of dried blood spots in newborns were collected for prospective studies, which were delivered to Neonatal Disease Screening Center of Zhejiang by cold chain transportation. The concentration of TREC and KREC, deletion of exon 7 from SMN1 gene in dried blood spots were detected by multiplex qPCR, reference geneRPP30 was used as an internal reference gene to ensure the accuracy of experiment, and positive newborns were further diagnosed by other laboratory tests and gene detection. Children samples from 1 case of SCID, 3 cases of XLA and 2 cases of SMA confirmed in our hospital were used for positive verification. The correlation between detected concentration of TREC/KREC and information in newborns were analyzed. Results: Onecase of SCID, 2 cases of XLA, 9 cases of SMA and 7 cases of other genetic metabolic diseases were identified. The positive predictive values of screening SCID, XLA and SMA in newborns were 2.44%, 2.78% and100% respectively. 1 case of SCID, 3 cases of XLA and 2 cases of SMA diagnosed in clinic were all detected by this method, and the sensitivity was 100%. The detected results of TREC/KREC was correlated with time of blood collection, sex, gestational age, weight and delivery mode of newborns. The r values were 0.162/0.187, 0.066/0.032, 0.045/0.042, −0.015/−0.088 and 0.014/0.068 respectively (all P0.05). Conclusions: It is feasible to screen jointly SCID XLA and SMA in newborns by Zhejiang current system of neonatal screening and applying multiple qPCR technology.

### 4.28. Spinal Muscular Atrophy

P82. **Nationwide SMA Screening in Serbia: Results and Impact After Sixteen Months of the First Genetic Newborn Test**
Nemanja Garai, Nemanja Radovanović, Jovan Pešović, Lana Radenković, Suzana Matijašević, Vanja Obadović, Sanja Madić, Goran Brajušković, Dušanka Savić-Pavićević and Miloš Brkušanin
University of Belgrade-Faculty of Biology, Centre for human molecular genetics, Beograd, Serbia

Introduction: Spinal muscular atrophy (SMA) leads to severe disability or death without early diagnosis and timely treatment. Newborn screening (NBS) for SMA ensures equitable access to life-saving therapies. Serbia stands among the first countries in the Balkans to implement SMA NBS, marking a significant healthcare advancement in the region. Materials and Methods: After a successful 17-month pilot screening of 12,000 newborns, Serbia launched its national SMA NBS program on 15 September 2023. The program involves collecting dried blood spots from 52 public and 6 private maternity hospitals, with samples analyzed at a centralized genetic laboratory. Results: By 15 January 2025, 81,276 newborns have been screened, leading to the identification of 8 SMA-positive infants. Immediate treatment was initiated for all, including 4 with 2 SMN2 copies, 3 with 3 copies, and 1 with 4 copies. This marks the first SMA incidence calculation in Serbia, with a rate of 1 in 10,159 births. Treatment decisions are guided by the Expert SMA Commission, established by the Serbian Republic Healthcare Insurance Fund, and are based on SMN2 copy number and comprehensive clinical data. While a delay in administering onasemnogene abeparvovec therapy remains a challenge, efforts to introduce a bridging approach are ongoing. Notably, all but one SMA-positive infant are currently asymptomatic; one showed symptoms at the time of diagnosis. Conclusion: These results demonstrate the transformative potential of NBS in reducing SMA-related morbidity and mortality. The involvement of the Expert SMA Commission ensures individualized treatment decisions. Addressing delays in gene therapy remains essential to maximize the program’s impact and improve long-term outcomes for all SMA-diagnosed infants.

P83. **Spinal Muscular Atrophy: A Priori Assessment of Extending Newborn Screening to the General Population in France—Recommendation**
Nadia Naour, Emmanuelle Ripoche, Ahcene Zehnati and Andrea Lasserre
HAS, evaluation and access to innovation department, Paris, France

HAS, evaluation and access to innovation department, Paris, France, HAS, evaluation and access to innovation department, Paris, France, HAS, evaluation and access to innovation department, Paris, France Introduction Spinal muscular atrophy (SMA) is a severe genetic disorder causing progressive muscle weakness and atrophy due to motor neurons loss. Early detection through newborn screening (NBS) is decisive for timely and improved clinical care. This study assesses the feasibility and benefits of extending SMA screening to the general newborn population in France. Method A systematic literature review and insights from a multidisciplinary working group were analyzed. Data were also gathered from the SMA France national register and the French DEPISMA pilot study. The assessment was based on six major criteria retained by French National Authority for Health (HAS), such as disease severity, treatment efficacy, screening test performance, time to screening results. The potential impact on public health was also considered. Results The estimated incidence of SMA is around 1/12,000 births in France. qPCR-based screening tests showed high sensitivity (≥95%) and specificity (≥99.9%). Screening results were obtained early enough to start treatment before one month of age (average 23 days [20, 27]). Pre-symptomatic treatment, enabled by early detection of SMA, significantly improves clinical outcomes, including motor functions, with follow-ups up to five years. The study also considered the economical and organizational aspects of nationwide screening, emphasizing the efficiency of NBS for SMA in high-income countries, like France, and the need for training of health professionals. Conclusion: Extending NBS with SMA is recommended in France, as it meets all HAS criteria for new candidate diseases in the NBS program. Nevertheless, further effort is needed to optimize screening strategies, address logistical challenges, and measure treatment effectiveness over time.

P84. **Health Technology Assessment of Newborn Screening for Spinal Muscular Atrophy in Ireland**
Karen Jordan, Mairin Ryan, Conor Teljeur, Michelle O’Neill, Arielle Maher, Patricia Harrington, Éanán Finnegan, Sarah Dillon, Laura Comber, Helen O’Donnell and Susan Spillane
Health Information and Quality Authority, Health Technology Assessment, Dublin, Ireland, University of Galway, Evidence Synthesis Ireland/Cochrane Ireland, Dublin, Ireland

Background: Spinal muscular atrophy (SMA) is a rare genetic disorder caused by pathogenic variation in the survival motor neuron 1 (SMN1) gene, resulting in symptoms ranging from mild to severe muscle weakness. While there is no known cure for SMA, disease-modifying treatments can alter the clinical course. Methods: A health technology assessment (HTA) was undertaken to inform decision making regarding the potential addition of SMA to the national newborn bloodspot screening programme (NNBSP) in Ireland. An advisory group was convened to provide expert input. Results: Screening was found to accurately detect homozygous deletion of SMN1, with low instances of false positives. Earlier intervention could yield considerable clinical benefits by preventing or reducing irreversible motor neuron loss and disease progression. Screening would result in changes in treatment pathways for patients identified through screening relative to existing practice in Ireland, which, based on the reimbursement criteria for available disease-modifying drugs at the time of analysis, would have significant resource implications. The 5-year incremental budget impact of screening (*n*~58,000 infants per year) was estimated at ~€17.7 million (95% CI: €5.1 to €40.5 m); cost effectiveness and affordability were highly dependent on treatment costs. Evaluation of organisational aspects identified critical enablers including the use of multiplex assays and the expansion of laboratory capacity. Assessment against an ethics framework identified several important issues, such as those relating to SMA classification. Policy impact: The HTA findings informed a recommendation by the National Screening Advisory Committee in Ireland to the Minister for Health to add SMA to the NNBSP.

### 4.29. Tyrosinaemia

P85. **Starting Afresh: Newborn Screening for Tyrosinaemia Type 1—The New Zealand Experience**
Mark de Hora, Detlef Knoll, Divanisova Sandra, Shonal Krishna, Emma Glamuzina, Ryder Bryony, Natasha Heather, Dianne Webster and Wilson Callum
LabPlus, Auckland City Hospital, Newborn Screening, Auckland, New Zealand, Starship Children’s Hospital, Clinical Metabolic Service, Auckland, New Zealand, Starship Children’s Hospital, Clinical Metabolic Service, Auckland, New Zealand

Introduction Newborn screening (NBS) for tyrosinaemia type 1 (TYR1), using tyrosine measurements, was introduced to New Zealand in 2006. Between 2006 and 2017, 2 confirmed cases of TYR1 were missed by screening. Additionally, 50 false positive (TP) tests were encountered each year. Screening was suspended whilst a more precise methodology using succinylacetone (SUAC) analysis was established. Methods SUAC measurement was incorporated into screening that included amino acids and acylcarnitines. Precision, linearity, accuracy, recovery, and sample stability were evaluated. Screening thresholds were established using 1079 NBS specimens and reference data from the Collaborative Laboratory Integrated Reports (CLIR). Since screening was re-introduced, 135,062 NBS samples from 126,393 babies had screening for TYR1. The number of false positives (FP), true positives (TP), screening specificity and positive predictive value (PPV) were calculated. Results The method was linear to 125 µmol/L. The between batch variation was 12.4–13.6%. The method compared well with other laboratories supplying data to a quality assurance scheme. Recovery was 88–112% but lower at higher concentration ranges. Measurements declined by 20% after 10 days. The median value was 2.2 µmol/L (CLIR = 2.04). The screening threshold was set at 5.5 µmol/L but changed to 2.5 when new instrumentation was implemented. Screening data revealed 135,061 negative NBS results, and no FP screens were encountered. There was 1 TP positive NBS result (SUAC = 21.7 µmol/L) and no missed cases reported. Specificity and PPV were 100%. Conclusions: Screening for TYR1 appears to be sufficiently sensitive and specific. Evaluation of screening over a much longer timeframe is warranted.

### 4.30. Miscellaneous

P86. **Clinical Characteristics and Novel ZEB2 Gene Mutation Analysis of Three Chinese Patients with Mowat-Wilson Syndrome**
Bingjuan Han
Jinan maternity and child care hospital, newborn screening center, Jinan, China, People’s Republic

Mowat-Wilson syndrome (MWS) is an autosomal dominant disease caused by a pathogenic variant of the ZEB2 gene. The main clinical manifestations include special facial features, Hirschsprung disease (HSCR), global developmental delay and other congenital malformations. Here, we summarize the clinical characteristics and genetic mutation analysis of three Chinese patients with MWS. Patients and Methods: The clinical characteristics of the patients were monitored and the treatment effect was followed up. DNA was extracted from peripheral blood and analyzed by sequencing. Whole exome sequencing was then performed. Results: Three novel ZEB2 gene mutations were identified in 3 patients c.1147_1150dupGAAC, p.Q384Rfs*7, c.1137_1146del TAGTATGTCT, p.S380Nfs *13 and c.2718delT, p.A907Lfs*23). They all had special facial features, intellectual disability, develop mental delay, microcephaly, structural brain abnormalities and other symptoms. After long-term regular rehabilitation treatment, the development quotient of each functional area of the patient was slightly improved. Conclusion: Our study expanded the mutation spectrum of ZEB2 and enriched our understanding of the clinical features of MWS. It also shows that long-term standardized treatment is of great significance for the prognosis of patients.

### 4.31. Adrenoleukodystrophy

P87. **Universal Newborn Screening for X-Linked Adrenoleukodystrophy: A Pioneering Initiative in Southern Spain**
María Isabel Cabrera-González, Marta García de Burgos, José Miguel Ramos-Fernández, Rocío Calvo-Medina, Yolanda de Diego-Otero, Javier Blasco-Alonso and Raquel Yahyaoui
Hospital Regional Universitario de Málaga, Newborn Screening Laboratory, Málaga, Spain

Background: X-linked adrenoleukodystrophy (XALD) is the most prevalent leukodystrophy and peroxisomal disorder, with an estimated prevalence of 1 in 10,000 live births. Early detection is crucial for timely treatment of adrenal insufficiency, and in cases of cerebral XALD, hematopoietic stem cell transplantation can be curative if performed in the early stages of the disease. In July 2022, we launched the first universal prospective pilot study for XALD newborn screening (NBS) in Europe. Methods: Between July 2022 and March 2024, C24:0-LPC and C26:0-LPC levels were measured in dried blood spots (DBS) from 75,000 newborns using an in-house LC-MS/MS method. For newborns with positive results, a second DBS sample was collected, followed by biochemical and genetic analysis. Results: Among 75,000 newborns screened, 16 tested positive. Two with severe neonatal symptoms caused by pathogenic variants in HSD17B4 and one with PEX6 variants died before six months of age. The remaining newborns are asymptomatic; eight were confirmed to carry variants in the ABCD1 gene, and five are still under investigation. So far, two relatives of XALD-affected newborns have also been diagnosed. Conclusion: This study represents the first universal NBS for XALD in Europe. Preliminary findings suggest a higher prevalence of peroxisomal disorders in our population than previously estimated (detection rate: 1 in 4687). Besides XALD, the screening identifies other peroxisomal diseases characterized by elevated C26:0-LPC levels. Universal NBS for XALD enables early intervention, genetic counseling, carrier detection, and the diagnosis of other at-risk family members, underscoring its clinical value.

