# Correction: Hall et al. Oral and Poster Abstracts of the 13th ISNS European Regional Meeting. *Int. J. Neonatal Screen.* 2025, *11*, 21

**DOI:** 10.3390/ijns11030049

**Published:** 2025-06-24

**Authors:** Kate Hall, Peter C. J. I. Schielen, Dimitris Platis

**Affiliations:** 1 International Society for Neonatal Screening, Reigerskamp 273, 3607 HP Maarssen, The Netherlands; kate@pdsoft.co.uk; 2 Department of Newborn Screening, Institute of Child Health, 11527 Athens, Greece; dplatis@ich.gr

The authors wish to make the following correction to their paper published in the *International Journal of Neonatal Screening* [1]:

1. In the Introduction, the authors would like to update “P64 Metyl Malonic Aciduria” to “P64 Methyl Malonic Aciduria”.

2. In I24, the authors would like to update affiliation 1 to “Laboratory of Newborn screening and Diagnostics of IEM, Centre for Medical Genetics, Vilnius University Hospital Santaros Klinikos, Vilnius, Lithuania”.

3. In I24, the authors would like to update the following sentence: “Since 1975, when the NBS was launched, a total of 1,894,509 newborns have been tested and 445 diagnoses have been confirmed.” The authors would like to update the number 445 to 452.

4. In O05, the authors would like to update the author order to “Michela Perrone Donnorso, Michela Cassanello, Luisella Alberti, Maria Cristina Schiaffino, Andrea Mascagni, Paola Vannini, Elvira Sondo, Cristina Cereda and Mohamad Maghnie”.

5. In P01, the authors would like to update the author order to “Hafsa Majid, Khairunnisa Mukhtiar, Muhammad Raza, Lena Jafri, Azeema Jamil, Nasir Ali Khan and Aysha Habib Khan”.

6. In P02, the authors would like to update the title to “21-deoxycortisone: A Novel Newborn Screening Marker of CAH due to 21-hydroxylase Deficiency”.

7. In P02, the authors would like to update the author order to “Mark de Hora, Natasha Heather, Dianne Webster, Benjamin Albert and Paul Hofman”.

8. In P04, the authors would like to update the author order to “Sara Colja, Daša Perko, Eva Kozjek, Domen Trampuž, Neža Založnik, Urh Grošelj, Ana Drole Torkar, Matej Mlinarič and Barbka Repič Lampret”.

9. In P08, the authors would like to update the author order to “Tomas Honzik, Jiri Zach, Richard Plavka, Truong An Nguyen, Jan Janota, Katarina Ticha, Zbynek Stranak, Klara Berkova, Jitka Sokolova, Jakub Krijt, Petr Chrastina, Kristyna Nelicova, Josef Bartl, Samuel Stanovsky and Viktor Kozich”.

10. In P09, the authors would like to update the author order to “Maria Lucia Tommolini, Maria Concetta Cufaro, Silvia Valentinuzzi, Mirco Zucchelli, Ilaria Cicalini, Alberto Frisco, Gessica Di Carlo, Sara Verrocchio, Daniela Semeraro, Ines Bucci, Vincenzo De Laurenzi, Luca Federici, Damiana Pieragostino and Claudia Rossi”.

11. In P13, the authors would like to update the author order to “Anna-Maria Charatsi, Meriem Mastouri, Caroline Eisele, Patricia Borde, Marizela Kulisic, Abdelkader Heddar and Isabel De la Fuente Garcia”.

12. In P17, the authors would like to update the author order to “Domen Trampuž, Eva Kozjek, Sara Colja, Daša Perko, Neža Založnik, Ana Drole Torkar, Matej Mlinarič, Urh Grošelj and Barbka Repič Lampret”.

13. In P22, the authors would like to update the author order to “Onanong Jaruan, Rotjanapan Pankanjanato, Pawinee Innark, Kannikar Punnapum, Suphattra Auttarawanit, Wiroj Puangtabtim and Hansa Thaisri”.

14. In P23, the authors would like to update the author order to “Felix Votava, Renata Gaillyová, Veronika Skalická, Milan Macek Jr, Andrea Holubová, Monika Hedelová, Hana Vinohradská, Eva Hlídková, David Friedecký, Tomáš Adam, Karolina Pešková, Viktor Kožich, Petr Chrastina and Iveta Valášková”.

15. In P25, the authors would like to update the author order to “Emmanuelle Ripoche, Nadia Naouir, Pascale Zagury and Andrea Lasserre”.

16. In P26, the authors would like to update the author order to “Barbka Repič Lampret, Matej Mlinarič, Mojca Žerjav Tanšek, Ana Drole Torkar, Žiga Iztok Remec, Sara Colja, Eva Kozjek, Domen Trampuž, Daša Perko, Neža Založnik and Urh Grošelj”.

17. In P28, the authors would like to update the author order to “Issam Khneisser and Dimitrios Platis”.

18. In P29, the authors would like to update the author order to “Andrii Lipatnikov, Tatiana Ivanova, Nataliia Samonenko, Mariia Haidei, Maryna Patsora, Alexandr Buryak, Volodymyr Olkhovych, Nataliia Mytsyk, Nataliia Olkhovich, Oksana Barvinska and Larisa Nikonova”.

19. In P32, the authors would like to update the author order to “Lukasz Krych, Gjorgji Madjarov, M. Carmen Garrido Navas, Zoran Velkoski, Marija Chaushevska and Chris Kyriakidis”.

20. In P33, the authors would like to update the author order to “Ana Drole Torkar, Blanka Ulaga, Domen Trampuž, Tine Tesovnik, Daša Perko, Barbka Repič Lampret, Eva Kozjek, Marusa Debeljak, Vanja Cuk, Sara Colja, Žiga Iztok Remec, Tadej Battelino, Ana Klinc, Urh Grošelj, Mojca Žerjav Tanšek, Matej Mlinarič and Jernej Kovac”.

21. In P43, the authors would like to update the author order to “Michela Perrone Donnorso, Giancarlo la Marca, Michela Cassanello, Elvira Sondo, Francesca Nastasia Perri, Maria Cristina Schiaffino and Mohamad Maghnie”.

22. In P47, the authors would like to remove the abstract and mark the poster as withdrawn.

23. In P52, the authors would like to update the author order to “Joery den Hoed, Terence Fabella and Jan Schouten”.

24. In P53, the authors would like to update the author order to “Terence Diane Fabella, Erik Sistermans, Lidewij Henneman, Gerard Pals, Joery den Hoed, Carmencita Padilla, Eva Maria Cutiongco-de la Paz and Jan Schouten”.

25. In P56, the authors would like to remove the abstract and mark the poster as withdrawn.

26. In P59, the authors would like to update the author order to “Luisa-Gabriela Bogos, Maria-Andreea Soporan, Ioana-Ecaterina Pralea, Radu-Cristian Moldovan, Sheilah Severino Snorrason, Leifur Franzson, Freyr Jóhannsson, Anca-Dana Buzoianu and Cristina-Adela Iuga”.

27. In Section 4.21, the authors would like to update the section title to “Methyl Malonic Aciduria”.

28. In P64, the authors would like to update the author order to “Jun He, Huiming Yan and Xia Li”.

29. In P65, the authors would like to update the author order to “Trine Tangeraas, Olve Moldestad, Asbjørg Stray-Pedersen, Janne Strand, Yngve Thomas Bliksrud, Kiarash Tazmini, Cathrin Lytomt Salvador, Erle Kristensen, Magnus Odin Resløkken Dahlseng, Julie Hellem-Aaaby, Marit Brynjulvsen, Siv Merete Løvoll, Andreas Øberg and Berit Woldseth”.

30. In P69, the authors would like to update the author order to “Gulnara Svyatova, Alexandra Murtazaliyeva, Anastassiya Ge, Vladislav Sklyarov and Madina Amantayeva”.

31. In P70, the authors would like to update the title to “Outcome of 2-year Expanded Newborn Screening Program in Ukraine”.

32. In P75, the authors would like to update the author order to “Karolina Peskova, Anna Sediva, Adam Klocperk, Marketa Bloomfield, Petra Hedvicakova, Emilie Vyhnalkova, Jakub Hodik, Petr Chrastina, Lenka Dvorakova and Jana Haberlova”.

33. In P77, the authors would like to update the author order to “Milica Pesevska, Violeta Anastasovska, Nikolina Zdraveska and Dijana Plaseska-Karanfilska”.

34. In P80, the authors would like to update the author order to “Ana Argudo-Ramírez, AECOM’ NBS Working Group—Pere Soler-Palacín, Lola Rausell-Félix, Elena Llorente-Martín, Alba Berzal-Serrano, Raquel Yahyahoui, Beatriz de Felipe Carmen Delgado-Pecellin, Alejandra González-Delgado, Cristobal Colón-Mejeras, Andrea Martín-Nalda, Marta Piedelobo and Judit García-Villoria”.

35. In P81, the authors would like to update the author order to “Rulai Yang, Chi Chen, Jianbin Yang and Chao Zhang”.

36. In P82, the authors would like to update the author order to “Nemanja Garai, Nemanja Radovanović, Jovan Pešović, Lana Radenković, Suzana Matijašević, Vanja Obadović, Sanja Madić, Goran Brajušković, Dušanka Savić-Pavićević and Miloš Brkušanin”.

37. In P84, the authors would like to update the author order to “Karen Jordan, Mairin Ryan, Conor Teljeur, Michelle O’Neill, Arielle Maher, Patricia Harrington, Éanán Finnegan, Sarah Dillon, Laura Comber, Helen O’Donnell and Susan Spillane”.

38. In P87, the authors would like to update the author order to “María Isabel Cabrera-González, Marta García de Burgos, José Miguel Ramos-Fernández, Rocío Calvo-Medina, Yolanda de Diego-Otero, Javier Blasco-Alonso and Raquel Yahyaoui”.

We would like to apologize for any inconvenience caused to the readers by these changes. The changes do not affect the scientific results. This correction was approved by the Academic Editor. The original publication has also been updated.
